# GEO-CEOS stage 4 validation of the Satellite Image Automatic Mapper lightweight computer program for ESA Earth observation level 2 product generation – Part 2: Validation

**DOI:** 10.1080/23312041.2018.1467254

**Published:** 2018-06-11

**Authors:** Andrea Baraldi, Michael Laurence Humber, Dirk Tiede, Stefan Lang

**Affiliations:** 1 Department of Agricultural Sciences, University of Naples Federico II, Portici, Italy; 2 Department of Geographical Sciences, University of Maryland, College Park, MD, USA; 3 Department of Geoinformatics – Z_GIS, University of Salzburg, Salzburg, Austria; 4 Italian Space Agency (ASI), Rome, Italy; 5 University of Melbourne, Australia

**Keywords:** Artificial intelligence, binary relationship, Cartesian product, cognitive science, color naming, connected-component multi-level image labeling, deductive inference, earth observation, land cover taxonomy, high-level (attentive) and low-level (pre-attentional) vision, hybrid inference, image classification, image segmentation, inductive inference, machine learning-from-data, outcome and process quality indicators, radiometric calibration, remote sensing, surface reflectance, thematic map comparison, top-of-atmosphere reflectance, two-way contingency table, unsupervised data discretization/vector quantization, validation

## Abstract

ESA defines as Earth Observation (EO) Level 2 information product a multi-spectral (MS) image corrected for atmospheric, adjacency, and topographic effects, stacked with its data-derived scene classification map (SCM), whose legend includes quality layers cloud and cloud-shadow. No ESA EO Level 2 product has ever been systematically generated at the ground segment. To fill the information gap from EO big data to ESA EO Level 2 product in compliance with the GEO-CEOS stage 4 validation (*Val*) guidelines, an off-the-shelf Satellite Image Automatic Mapper (SIAM) lightweight computer program was selected to be validated by independent means on an annual 30 m resolution Web-Enabled Landsat Data (WELD) image composite time-series of the conterminous U.S. (CONUS) for the years 2006 to 2009. The SIAM core is a prior knowledge-based decision tree for MS reflectance space hyperpolyhedralization into static (non-adaptive to data) color names. For the sake of readability, this paper was split into two. The present Part 2—Validation—accomplishes a GEO-CEOS stage 4 *Val* of the test SIAM-WELD annual map time-series in comparison with a reference 30 m resolution 16-class USGS National Land Cover Data (NLCD) 2006 map. These test and reference map pairs feature the same spatial resolution and spatial extent, but their legends differ and must be harmonized, in agreement with the previous Part 1 - Theory. Conclusions are that SIAM systematically delivers an ESA EO Level 2 SCM product instantiation whose legend complies with the standard 2-level 4-class FAO Land Cover Classification System (LCCS) Dichotomous Phase (DP) taxonomy.

## Public Interest Statement

Synonym of scene-from-image reconstruction and understanding, vision is an inherently ill-posed cognitive task; hence, it is difficult to solve and requires a priori knowledge in addition to sensory data to become better posed for numerical solution. In the inherently ill-posed cognitive domain of computer vision, this research was undertaken to validate by independent means a lightweight computer program for prior knowledge-based multi-spectral color naming, called Satellite Image Automatic Mapper (SIAM), eligible for automated near-real-time transformation of large-scale Earth observation (EO) image datasets into European Space Agency (ESA) EO Level 2 information product, never accomplished to date at the ground segment. An original protocol for wall-to-wall thematic map quality assessment without sampling, where legends of the test and reference map pair differ and must be harmonized, was adopted. Conclusions are that SIAM is suitable for systematic ESA EO Level 2 product generation, regarded as necessary not sufficient pre-condition to transform EO big data into timely, comprehensive, and operational EO value-adding information products and services.

## 1. Introduction

Jointly proposed by the intergovernmental Group on Earth Observations (GEO) and the Committee on Earth Observation Satellites (CEOS), the implementation plan for years 2005–2015 of the Global Earth Observation System of Systems (GEOSS) aimed at systematic transformation of multi-source Earth observation (EO) *big data* (IBM, 2016; Yang, Huang, Li, Liu, & Hu, 2017) into timely, comprehensive, and operational EO value-adding products and services (GEO, 2005), submitted to the GEO-CEOS Quality Assurance Framework for Earth Observation (QA4EO) calibration/validation (*Cal/Val*) requirements (Group on Earth Observation/Committee on Earth Observation Satellites (GEO/CEOS), 2010). The visionary goal of GEOSS cannot be considered fulfilled by the remote sensing (RS) community to date. In the terminology of philosophical hermeneutics, the problem is not a lack of sensory data, but our lack of knowledge in transforming big sensory data into quantitative/unequivocal *information-as-thing* and qualitative/equivocal *information-as-data-interpretation* (Capurro & Hjørland, 2003). Such a lack of knowledge causes the so-called data-rich, information-poor (DRIP) syndrome (Bernus & Noran, 2017), supported by undisputable observations (true-facts). For example, past and present EO image understanding systems (EO-IUSs) have been typically outpaced by the rate of collection of EO sensory data, whose quality and quantity are ever-increasing at an apparently exponential rate related to the Moore law of productivity (National Aeronautics and Space Administration (NASA), 2016).

To contribute toward the visionary goal of GEOSS, this interdisciplinary work aimed at filling an analytic and pragmatic information gap from EO big sensory data to systematic European Space Agency (ESA) EO Level 2 information product generation (CNES, 2015; Deutsches Zentrum fürLuft- und Raumfahrt e.V. (DLR) and VEGA Technologies, 2011; European Space Agency (ESA), 2015), never accomplished at the ground segment by any EO data provider to date (DLR & VEGA, 2011; ESA, 2015). ESA defines as EO Level 2 information product: (i) a single-date multi-spectral (MS) image, radiometrically calibrated into surface reflectance (SURF) values corrected for atmospheric, adjacency, and topographic effects, in compliance with the GEO-CEOS QA4EO *Cal* requirements (GEO-CEOS, 2010), stacked with (ii) its data-derived Scene Classification Map (SCM), whose legend includes quality layers cloud and cloud-shadow (ESA, 2015; DLR & VEGA, 2011; CNES, 2015). In practice, ESA EO Level 2 product generation is a *chicken-and-egg* dilemma, synonym of inherently ill-posed problem in the Hadamard sense (Hadamard, 1902); therefore, it is very difficult to solve. On the one hand, no effective and efficient understanding (mapping) of a sub-symbolic EO image into a symbolic SCM is possible if DNs (pixels) are affected by low radiometric quality (GEO-CEOS, 2010). On the other hand, no effective and efficient *Cal* of digital numbers (DNs) into SURF values corrected for atmospheric, topographic and adjacency effects is possible without an SCM, available a priori in addition to sensory data to enforce a statistic stratification (layered) principle, synonym of class-conditional data analytics (Baraldi, 2017; Baraldi et al., 2010b; Baraldi & Humber, 2015; Baraldi, Humber, & Boschetti, 2013; Bishop & Colby, 2002; Bishop, Shroder, & Colby, 2003; DLR & VEGA, 2011; Dorigo, Richter, Baret, Bamler, & Wagner, 2009; Lück & Van Niekerk, 2016; Riano, Chuvieco, Salas, & Aguado, 2003, Richter & Schläpfer, 2012a, 2012b; Vermote & Saleous, 2007). Well known in statistics, the principle of statistic stratification guarantees that “stratification will always achieve greater precision provided that the strata have been chosen so that members of the same stratum are as similar as possible in respect of the characteristic of interest” (Hunt & Tyrrell, 2012).

For the sake of readability, this paper is split into two. The preliminary Part 1 - Theory postulated as working hypothesis a necessary not sufficient pre-condition for a yet-unfulfilled GEOSS development (GEO-CEOS, 2005). The proposed working hypothesis is “ESA EO Level 2 product ⊂ EO image understanding (EO-IU) in operating mode ⊂ computer vision (CV) → GEOSS,” where relationship *subset-of*, denoted with symbol “⊃,” means specialization with inheritance from the superset to the subset, while dependence relationship *part-of* is denoted with symbol “→,” pointing from the supplier to the client in agreement with the standard Unified Modeling Language (UML) for graphical modeling of object-oriented software (Fowler, 2016). This working hypothesis postulates that no GEOSS can exist if the necessary not sufficient pre-condition of systematic ESA EO Level 2 product generation is not accomplished in advance at the ground segment. Hence, systematic ESA EO Level 2 product generation is considered a mandatory early stage in a hierarchical EO-IUS workflow, capable of scene-from-image reconstruction and understanding in operating mode to cope with the five Vs of big data, specifically, volume, variety, velocity, veracity, and value (IBM, 2016; Yang et al., 2017).

**Figure 1.**
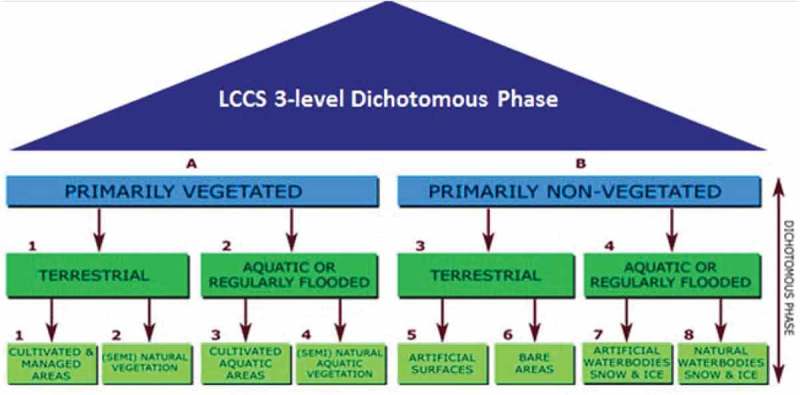
The fully nested 3-level 8-class FAO Land Cover Classification System (LCCS) Dichotomous Phase (DP) layers are: (i) vegetation versus non-vegetation, (ii) terrestrial versus aquatic, and (iii) managed versus natural or semi-natural. They deliver as output the following 3-level 8-class FAO LCCS-DP taxonomy. (A11) Cultivated and Managed Terrestrial (non-aquatic) Vegetated Areas. (A12) Natural and Semi-Natural Terrestrial Vegetation. (A23) Cultivated Aquatic or Regularly Flooded Vegetated Areas. (A24) Natural and Semi-Natural Aquatic or Regularly Flooded Vegetation. (B35) Artificial Surfaces and Associated Areas. (B36) Bare Areas. (B47) Artificial Waterbodies, Snow and Ice. (B48) Natural Waterbodies, Snow and Ice. The general-purpose, user- and application-independent 3-level 8-class FAO LCCS-DP taxonomy is preliminary to a user- and application-specific FAO LCCS Modular Hierarchical Phase (MHP) taxonomy of one-class classifiers (Di Gregorio & Jansen, 2000), refer to Figure 3 in the Part 1 of this paper.

In the words of Marr, “vision goes symbolic almost immediately, right at the level of zero-crossing (first-stage primal sketch), without loss of information” (Marr, 1982). In agreement with Marr’s intuition, our instantiation of an ESA EO Level 2 product generation is constrained as follows. (I) A single-date MS image is radiometrically corrected for atmospheric, adjacency, and topographic effects, automatically (without human-machine interaction) and in near real time. (II) It is stacked with its data-derived SCM, generated automatically and in near real time. The SCM legend must be general purpose and user and application independent. Unlike the non-standard SCM legend adopted by the Sentinel 2 imaging sensor-specific (atmospheric) Correction Prototype Processor (SEN2COR) developed by ESA to be run on user side (ESA, 2015; DLR & VEGA, 2011), the proposed SCM legend is selected equal to an “augmented” 3-level 9-class Dichotomous Phase (DP) taxonomy of the Food and Agriculture Organization of the United Nations (FAO)—Land Cover Classification System (LCCS) (Di Gregorio & Jansen, 2000). Such an “augmented” land cover (LC) class taxonomy in the 4D spatio-temporal scene-domain encompasses the standard fully nested 3-level 8-class FAO LCCS-DP legend in addition to a thematic layer “other” or “rest of the world,” which includes quality layers cloud and cloud-shadow; see Figure 1. (III) A GEO-CEOS stage 4 *Val* of the ESA EO Level 2 outcome and process is considered mandatory to comply with the GEO-CEOS QA4EO *Cal*/*Val* requirements (GEO-CEOS, 2010). By definition, a GEO-CEOS Stage 3 *Val* requires that “spatial and temporal consistency of the product with similar products are evaluated by independent means over multiple locations and time periods representing global conditions. In Stage 4 *Val*, results for Stage 3 are systematically updated when new product versions are released and as the time-series expands” (GEO-CEOS - Working Group on Calibration and Validation (WGCV), 2015).

**Figure 2.**
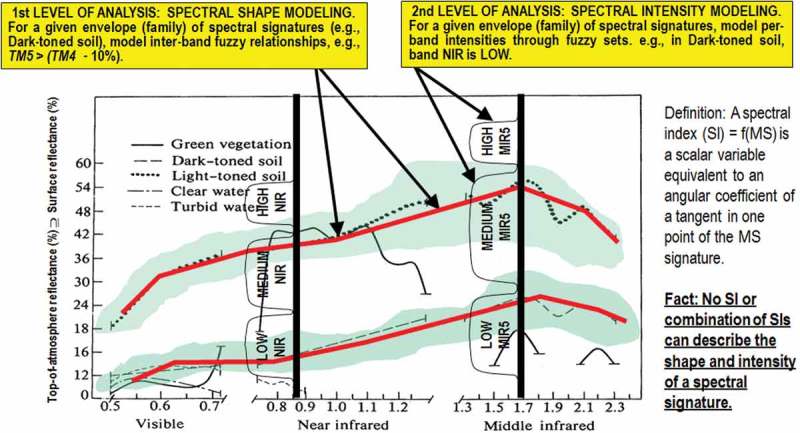
(same as Figure 5 in the Part 1 of this paper, duplicated for the sake of readability of the present Part 2). Examples of land cover (LC) class-specific families of spectral signatures in top-of-atmosphere reflectance (TOARF) values that include surface reflectance (SURF) values as a special case in clear sky and flat terrain conditions. A within-class family of spectral signatures (e.g., dark-toned soil) in TOARF values forms a buffer zone (hyperpolyhedron, envelope, manifold). The SIAM decision tree models each target family of spectral signatures in terms of multivariate shape and multivariate intensity information components as a viable alternative to multivariate analysis of spectral indexes. A typical spectral index is a scalar band ratio equivalent to an angular coefficient of a tangent in one point of the spectral signature. Infinite functions can feature the same tangent value in one point. In practice, no spectral index or combination of spectral indexes can reconstruct the multivariate shape and multivariate intensity information components of a spectral signature.

To contribute toward filling an analytic and pragmatic information gap from multi-source EO big imagery to systematic generation of ESA EO Level 2 product as constrained above, the primary goal of this interdisciplinary study was to undertake an original (to the best of these authors’ knowledge, the first) outcome and process GEO-CEOS stage 4 *Val* (GEO-CEOS WGCV, 2015) of an off-the-shelf Satellite Image Automatic Mapper™ (SIAM™) lightweight computer program for top-down (deductive) MS reflectance space hyperpolyhedralization into MS color names, superpixel detection, and vector quantization (VQ) quality assessment. Implemented in operating mode in the C/C++ programming language, the SIAM software toolbox is “lightweight” because it runs automatically (without human-machine interaction), in near real time (it is non-iterative and its computational complexity increases linearly with image size) and in tile streaming mode (it requires a fixed runtime memory occupation) (Baraldi, 2015, 2017; Baraldi, Puzzolo, Blonda, Bruzzone, & Tarantino, 2006; Baraldi et al., 2010a, 2010b; Baraldi, Gironda, & Simonetti, 2010c; Baraldi, 2011; Baraldi & Boschetti, 2012a, 2012b; Baraldi et al., 2013; Baraldi, Tiede, Sudmanns, Belgiu, & Lang, 2016, 2017; Baraldi & Humber, 2015). In addition to running on either laptop or desktop computers, the SIAM lightweight computer program is eligible for use in mobile application software or web services. Eventually provided with a mobile user interface, a mobile application software is a lightweight computer program specifically designed to run directly on mobile devices, such as tablet computers and smartphones. The core of the non-iterative SIAM software pipeline is a one-pass prior knowledge-based decision tree (expert system) for MS reflectance space hyperpolyhedralization (quantization, partitioning) into static (non-adaptive-to-data) color names, see Figure 2 and refer to Chapter 2 and Chapter 3 in the Part 1. Presented in the RS literature where enough information was provided for the implementation to be reproduced (Baraldi et al., 2006), the SIAM expert system for MS color naming is followed by a well-posed two-pass superpixel detector in the multi-level color map-domain (Dillencourt, Samet, & Tamminen, 1992; Sonka, Hlavac, & Boyle, 1994) and a per-pixel VQ error assessment for VQ quality assurance, in agreement with the GEO-CEOS QA4EO *Val* guidelines, refer to Figure 4 in the Part 1 of this paper.

**Figure 3.**
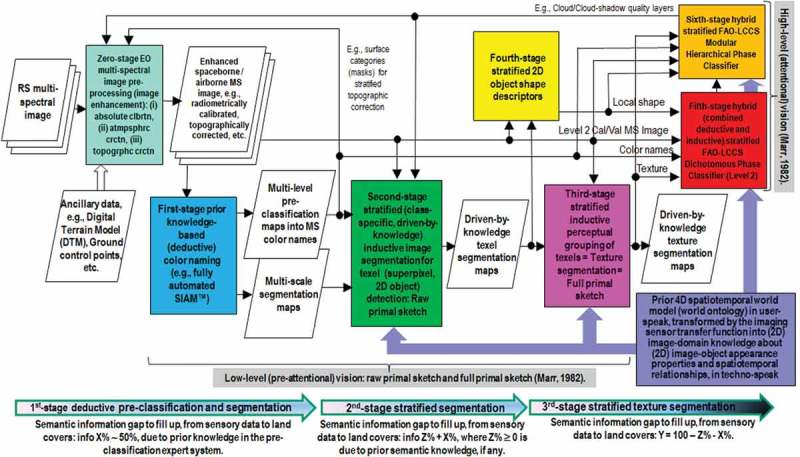
Six-stage hybrid (combined deductive and inductive) feedback EO image understanding system (EO-IUS) design, based on a convergence-of-evidence approach consistent with Bayesian naïve classification (Baraldi, 2017). Alternative to inductive feedforward EO-IUS architectures adopted by the RS mainstream, it supports a hierarchical approach to low-level (preliminary, general-purpose, sensor-, application- and user independent) EO image understanding followed by high-level (sensor-, application- and user-specific) EO image understanding (classification), consistent with the standard fully nested Land Cover Classification System (LCCS) taxonomy promoted by the Food and Agriculture Organization (FAO) of the United Nations (Di Gregorio & Jansen, 2000). For the sake of visualization each of the six data processing stages plus stage-zero for EO data pre-processing is depicted as a rectangle with a different color fill. Visual evidence stems from multiple information sources, specifically, numeric color values quantized into categorical color names, local shape, texture and inter-object spatial relationships, either topological or non-topological. An example of preliminary (low-level) general-purpose, user- and application-independent EO image classification taxonomy required by an ESA EO Level 2 Scene Classification Map (SCM) product is the 3-level 8-class FAO LCCS Dichotomous Phase (DP) legend, in addition to quality layers such as cloud and cloud-shadow. High-level EO image classification is user- and application-specific, where a thematic map product of Level 3 or superior is provided with a legend consistent with the FAO LCCS Modular Hierarchical Phase (MHP) taxonomy (Di Gregorio & Jansen, 2000); refer to Figure 3 in the Part 1 of this paper. Acronym SIAM stays for Satellite Image Automatic Mapper (SIAM), a lightweight computer program for MS reflectance space hyperpolyhedralization into a static vocabulary of MS color names, superpixel detection and vector quantization (VQ) quality assessment (Baraldi, 2017; Baraldi et al., 2006, 2010a, 2010b, 2010c; Baraldi, 2011; Baraldi & Boschetti, 2012a, 2012b; Baraldi et al., 2013; 2016; Baraldi & Humber, 2015).

**Figure 4.**
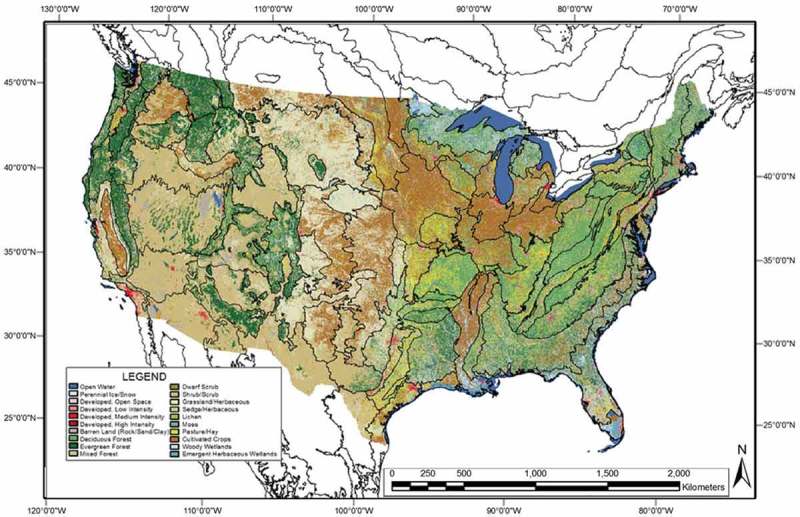
Reference USGS NLCD 2006 map of the CONUS. It is shown in the same scale and projection of the WELD 2006 composite depicted in Figure 6. Black lines across the USGS NLCD 2006 map represent the boundaries of the 86 EPA Level III ecoregions of the CONUS. The USGS NLCD 2006 map legend is shown on the left bottom side, also refer to Table 1.

There is a long history of prior knowledge-based MS reflectance space partitioners for static color naming developed, but never validated by space agencies, public organizations, and private companies for use in hybrid (combined deductive and inductive) EO-IUSs in operating mode, refer to Chapter 3 in the Part 1 of this paper. EO value-adding products and services delivered by existing hybrid EO-IUSs whose input is a MS image class-conditioned (masked) by static color names encompass a large variety of low-level EO image enhancement tasks, ranging from MS image compositing to atmospheric and topographic correction of top-of-atmosphere reflectance (TOARF) into SURF values (Ackerman et al., 1998; Baraldi et al., 2010c; Baraldi & Humber, 2015; Baraldi et al., 2013; Despini, Teggi, & Baraldi, 2014; DLR and VEGA, 2011; Dorigo et al., 2009; Lück & Van Niekerk, 2016; Luo, Trishchenko, & Khlopenkov, 2008; Richter & Schläpfer, 2012a, 2012b; Vermote & Saleous, 2007) and high-level EO image understanding applications, including EO image time-series classification, ranging from cloud/cloud-shadow detection to burned area recognition (Arvor, Madiela, & Corpetti, 2016; Baraldi, 2015; Baraldi et al., 2010a, 2010b; Boschetti, Roy, Justice, & Humber, 2015; DLR & VEGA, 2011; Lück & Van Niekerk, 2016; Muirhead & Malkawi, 1989; Simonetti, Simonetti, Szantoi, Lupi, & Eva, 2015; GeoTerraImage, 2015). To the best of these authors’ knowledge, none of these prior knowledge-based MS reflectance space partitioners presented in the RS literature has ever been submitted to a GEO-CEOS stage 4 *Val* process (GEO-CEOS WGCV, 2015), in compliance with the GEO-CEOS QA4EO *Val* requirements (GEO-CEOS, 2010). To fill this analytic and pragmatic lack, the proposed GEO-CEOS stage 4 *Val* outcome and process of an off-the-shelf SIAM lightweight computer program for prior knowledge-based MS reflectance space hyperpolyhedralization would be the first of its kind. Hence, the potential impact of the present study on the research and development (R&D), testing and validation of present or future hybrid EO-IUSs in operating mode, where static color naming is employed for MS image stratification purposes according to a convergence-of-evidence approach in agreement with a Bayesian naïve classification formulation (Baraldi, 2017; Matsuyama & Hwang, 1990), refer to Equation (3) in the Part 1 of this paper and see Figure 3, is expected to be relevant.

To comply with the GEO-CEOS stage 4 *Val* requirements (GEO-CEOS WGCV, 2015), outcome and process of an off-the-shelf SIAM computer program had to be validated by independent means on a radiometrically calibrated EO image time-series at large spatial extent. The open-access U.S. Geological Survey (USGS) 30 m resolution annual Web Enabled Landsat Data (WELD) image composite of the conterminous U.S. (CONUS) for the years 2006 to 2009, radiometrically calibrated into TOARF values (Homer, Huang, Yang, Wylie, & Coan, 2004; Roy et al., 2010; WELD, 2016), was identified as a viable input dataset. The 30 m resolution 16-class U.S. National Land Cover Data (NLCD) 2006 map, delivered in 2011 by the USGS Earth Resources Observation Systems (EROS) Data Center (EDC) (Environmental Protection Agency (EPA), 2007; Vogelmann et al., 2001; Vogelmann, Sohl, Campbell, & Shaw, 1998; Wickham, Stehman, Fry, Smith, & Homer, 2010; Wickham et al., 2013; Xian & Homer, 2010), was selected as the reference thematic map at the CONUS spatial extent. The 16-class NLCD map legend is described in Table 1. To account for typical non-stationary geospatial statistics, the USGS NLCD 2006 thematic map was partitioned into 86 Level III ecoregions of North America collected from the Environmental Protection Agency (EPA) (Environmental Protection Agency (EPA), 2013; Griffith & Omernik, 2009).

**Table 1.** Definition of the USGS NLCD 2001/2006/2011 classification taxonomy, Level II.^2^Alaska only, consisting of 16 land cover (LC) class names. For further details, refer to the “National Land Cover Database 2006 (NLCD 2006),” Multi-Resolution Land Characteristics Consortium (MRLC), 2013. The right column instantiates a possible binary relationship R: A ⇒ B ⊆ A × B from set A = 16-class NLCD legend to set B = 2-level 4-class Dichotomous Phase (DP) taxonomy of the Food and Agriculture Organization of the United Nations (FAO)—Land Cover Classification System (LCCS) (Di Gregorio & Jansen, 2000), refer to Figure 1

NLCD 2001/2006/2011 classification scheme (legend), level II				LCCS-DP, level 1: A = Veg, B = Non-veg, and level 2: 1 = Terrestrial, 2 = Aquatic
Code	ID	Name	Land cover (LC) class definition	ID
11	OW	Open water	OW: Areas of open water, generally with less than 25% cover of vegetation or soil	B4—Non-vegetated aquatic
12	PIS	Perennial Ice/Snow	PIS: Areas characterized by a perennial cover of ice and/or snow, generally greater than 25% of total cover.	B4
21 22 23 24	DOS DLI DMI DHI	Developed, Open Space Developed, Low Intensity Developed, Medium Intensity Developed, High Intensity	DOS: Includes areas with a mixture of some constructed materials, but mostly vegetation in the form of lawn grasses. Impervious surfaces account for less than 20 percent of total cover. These areas most commonly include large-lot single-family housing units, parks, golf courses, and vegetation planted in developed settings for recreation, erosion control, or aesthetic purposes. DLI, DMI, DHI: refer to the “National Land Cover Database 2006 (NLCD 2006),” Multi-Resolution Land Characteristics Consortium (MRLC), 2013.	B3—Non-vegetated terrestrial/A1—Vegetated terrestrial
31	BL	Barren Land (Rock/Sand/Clay)	BL: Barren areas of bedrock, desert pavement, scarps, talus, slides, volcanic material, glacial debris, sand dunes, strip mines, gravel pits, and other accumulations of earthen material. Generally, vegetation accounts for less than 15% of total cover. As a consequence of this constraint, class BL covers only 1.21% of the CONUS total surface.	B3
41 42 43	DF EF MF	Deciduous Forest Evergreen Forest Mixed Forest	DF: Areas dominated by trees generally greater than 5 m tall, and greater than 20% of total vegetation cover. More than 75 percent of the tree species shed foliage simultaneously in response to seasonal change. EF: Areas dominated by trees generally greater than 5 m tall, and greater than 20% of total vegetation cover. More than 75 percent of the tree species maintain their leaves all year. Canopy is never without green foliage. MF: Mixed Forest—Areas dominated by trees generally greater than 5 m tall, and greater than 20% of total vegetation cover. Neither deciduous nor evergreen species are greater than 75 percent of total tree cover.	A1
51 52	-SS	Dwarf Scrub ^2^ Scrub/Shrub	SS: Areas dominated by shrubs; less than 5 m tall with shrub canopy typically greater than 20% of total vegetation. This class includes true shrubs, young trees in an early successional stage or trees stunted from environmental conditions. The aforementioned definition of class BL means that class SS may feature a vegetated cover which accounts for 15% of total cover or more.	A1/B3
71 72 73 74	GH-	Grassland/Herbaceous Sedge Herbaceous ^2^ Lichens ^2^ Moss ^2^	GH: Areas dominated by grammanoid or herbaceous vegetation, generally greater than 80% of total vegetation. These areas are not subject to intensive management such as tilling, but can be utilized for grazing. The aforementioned definition of class BL means that class GH may feature a vegetated cover that accounts for 15% of total cover or more.	A1/B3
81 82	PH CC	Pasture/Hay Cultivated Crops	PH: Areas of grasses, legumes, or grass-legume mixtures planted for livestock grazing or the production of seed or hay crops, typically on a perennial cycle. Pasture/hay vegetation accounts for greater than 20 percent of total vegetation. CC: Areas used for the production of annual crops, such as corn, soybeans, vegetables, tobacco, and cotton, and also perennial woody crops such as orchards and vineyards. Crop vegetation accounts for greater than 20% of total vegetation. This class also includes all land being actively tilled.	A1
90 95	WW EHW	Woody Wetlands Emergent Herbaceous Wetland	WW: Areas where forest or shrubland vegetation accounts for greater than 20 percent of vegetative cover and the soil or substrate is periodically saturated with or covered with water. EHW: Areas where perennial herbaceous vegetation accounts for greater than 80% of vegetative cover and the soil or substrate is periodically saturated with or covered with water.	A2—Vegetated aquatic

In the proposed experimental framework, the test SIAM-WELD map time-series and the reference USGS NLCD 2006 map share the same spatial extent and spatial resolution, but their map legends are not the same, differing in both semantics and cardinality. These working hypotheses are neither trivial nor conventional in the RS literature, where thematic map quality assessments typically adopt a sampling strategy, either probabilistic (random) or non-random (Baraldi et al., 2013), and assume that the test and reference thematic map dictionaries coincide (Stehman & Czaplewski, 1998). Starting from a stratified random sampling protocol presented in literature (Baraldi et al., 2013), the present Part 2 - Validation proposes an original protocol for wall-to-wall comparison without sampling of two thematic maps featuring the same spatial extent and spatial resolution, but whose legends can differ. This novel protocol incorporates two original contributions of the Part 1 where, first, a hybrid (combined deductive and inductive) eight-step guideline was proposed to streamline a human decision maker in the identification of a binary relationship, R: A ⇒ B ⊆ A × B, from categorical variable A to categorical variable B estimated from the same population, where A ≠ B in general and A × B is the 2-fold Cartesian product (product set). This is an inherently ill-posed (equivocal, subjective) *information-as-data-interpretation* process (Capurro & Hjørland, 2003) belonging to the multi-disciplinary domain of cognitive science, refer to Figure 13 in the Part 1. The proposed hybrid eight-step guideline is of practical use because identification of a binary relationship, R: A ⇒ B, is mandatory to guide the interpretation process of a bivariate frequency table, BIVRFTAB = FrequencyCount(A × B) ≠ R: A ⇒ B ⊆ A × B, where A ≠ B in general. Only if A = B then BIVRFTAB becomes equal to the well-known square and sorted confusion matrix (CMTRX), where the main diagonal guides the interpretation process. Second, version 2 of a categorical variable-pair index of association (harmonization, matching) in a binary relationship, R: A ⇒ B, where A ≠ B in general, CVPAI2(R: A ⇒ B) ∈ [0, 1], was proposed to cope with the entity-relationship conceptual model shown in Figure 18 of the Part 1.

**Figure 5.**
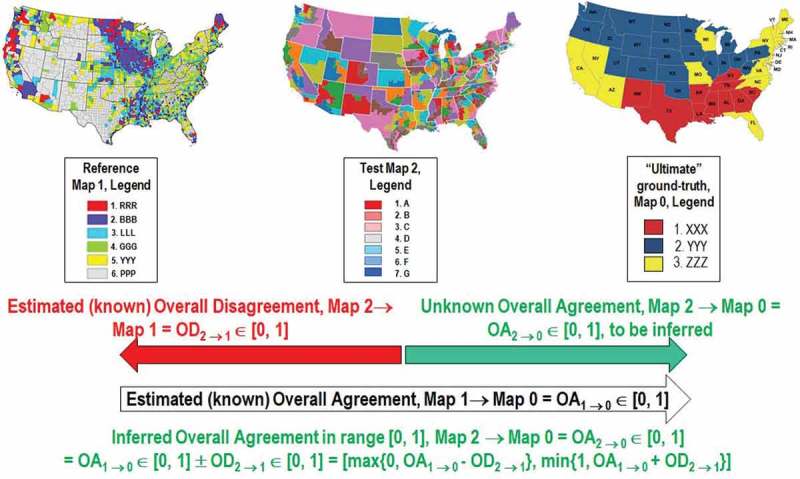
Superposition principle in a sequence of thematic map accuracy estimates.

**Figure 6.**
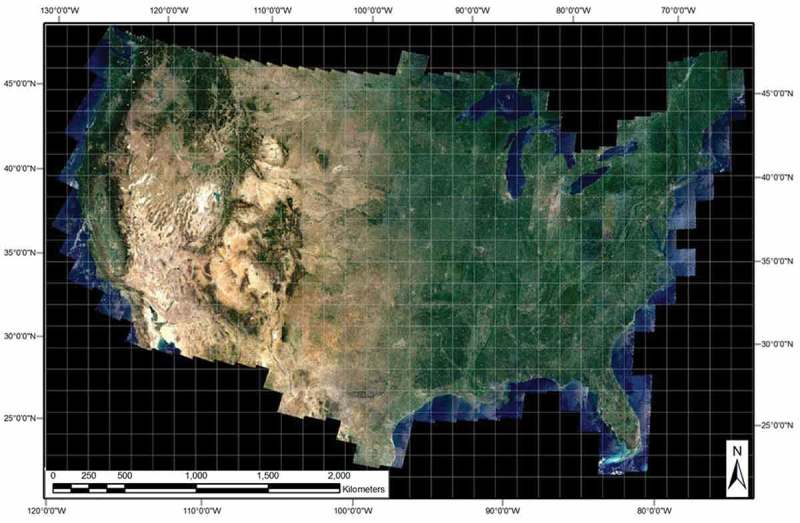
30 m resolution annual Web-Enabled Landsat Data (WELD) image composite for the year 2006 (December 2005 to November 2006) of the conterminous U.S. (CONUS), radiometrically calibrated into top-of-atmosphere reflectance (TOARF) values. Depicted in true colors (red: Band 3, 0.63–0.69 μm; green: Band 2, 0.53–0.61 μm, and blue: Band 1, 0.45–0.52 μm), linearly stretched for visualization purposes. The white grid shows locations of the 501 WELD tiles of the CONUS. Each tile is 5000 × 5000 pixels in size, covering a surface area of 150 × 150 km. Pixels are geographically projected in the Albers Equal Area projection.

**Figure 7.**
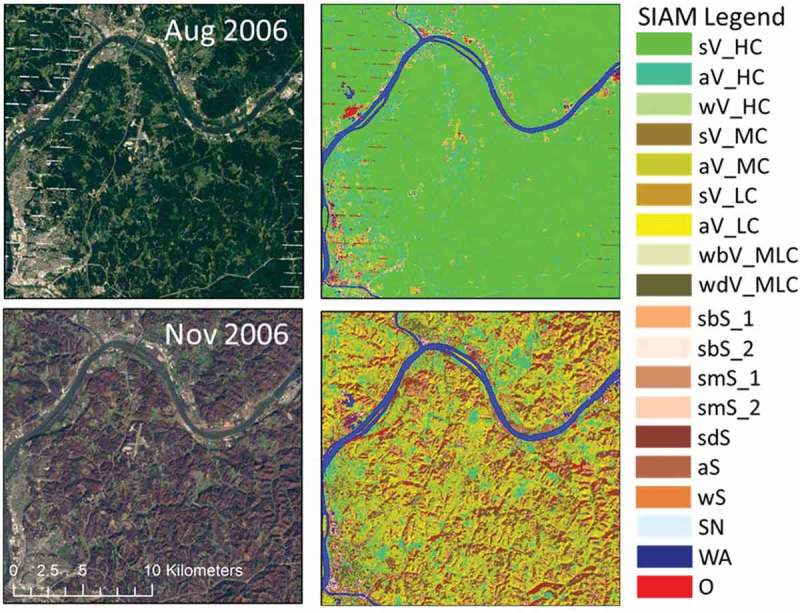
Changes through time of the 19-class SIAM spectral macro-category labels due to vegetation phenology affecting the monthly WELD composite. *Left side*: 30 m resolution monthly WELD composites, radiometrically calibrated into top-of-atmosphere reflectance (TOARF) values, for August and November 2006, showing an area predominantly covered by broadleaf forest in the Mid-Western United States (Ohio). Depicted in true colors (red: Band 3, 0.63–0.69 μm; green: Band 2, 0.53–0.61 μm, and blue: Band 1, 0.45–0.52 μm). To allow inter-image comparison, the two images are displayed with an identical contrast stretch. *Right side*: SIAM-WELD color maps generated from the two WELD images shown on the left side. The SIAM map legend, consisting of 19 spectral macro-categories, is shown on the right side, also refer to Table 2.

**Figure 8.**
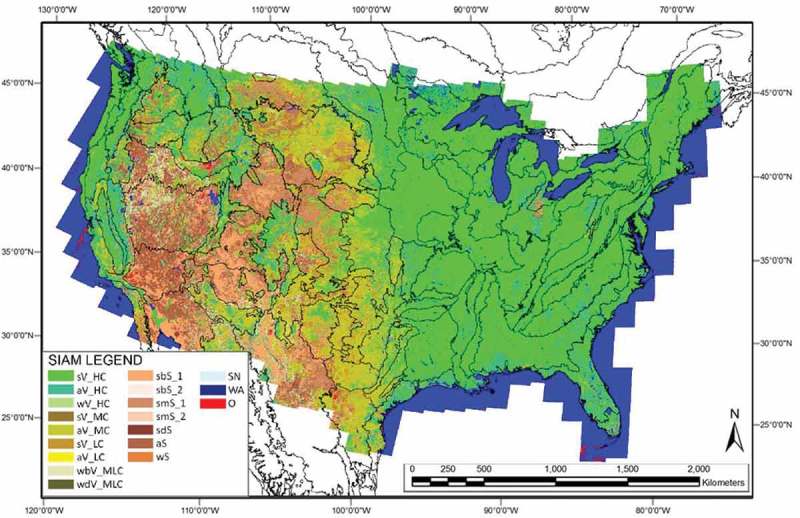
Automatically generated SIAM-WELD 2006 color map depicted at an intermediate discretization level of 48 color names, reassembled into 19 spectral macro-categories by an independent human expert. Black lines across the SIAM-WELD 2006 map represent the boundaries of the 86 EPA Level III ecoregions of the CONUS. The reassembled 19-class SIAM map legend is depicted at bottom left, also refer to Table 2.

**Figure 9.**
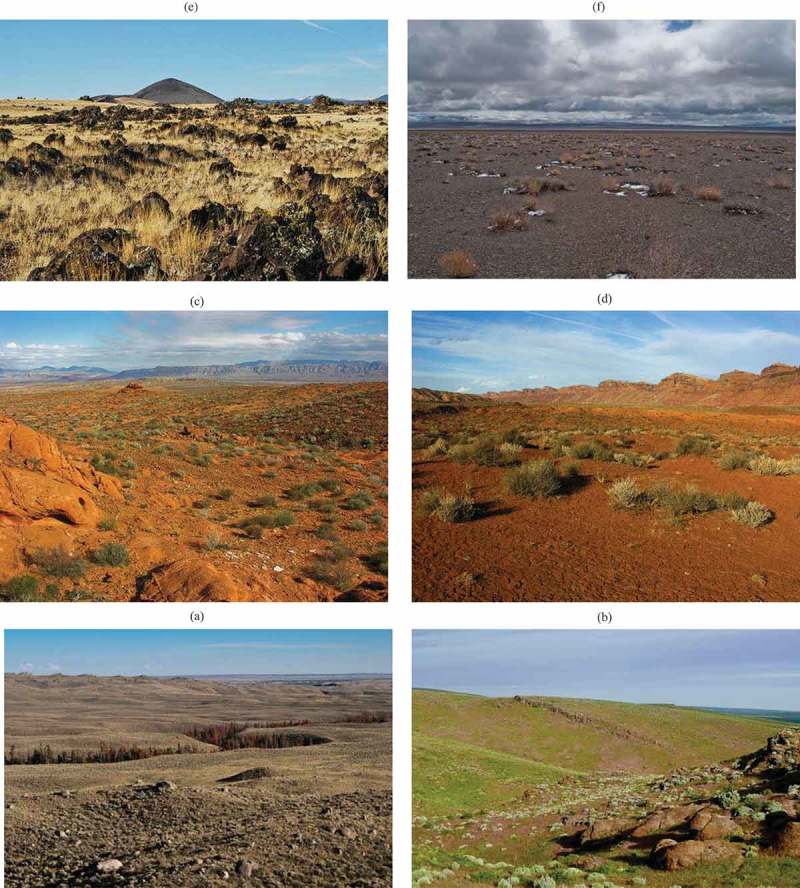
Examples of geographic locations mapped as vegetation classes “*Scrub/Shrub*” (SS) or “*Grassland/Herbaceous*” (GH) in the USGS NLCD 2006 reference map (refer to Table 1) and predominantly as bare soil spectral categories (sbS_1, SmS_1, aS) in the SIAM-WELD 2006 test map (refer to Table 2), as pointed out in Table 8. The SIAM’s color names sbS_1, SmS_1, and aS mean that, from space, with a pixel size of 30 m × 30 m = 900 m^2^, the contribution of sparse vegetation, rangeland, cheatgrass, dry long grass or short grass as foreground, mixed with a background of sand, clay, or rocks, like those shown in these pictures, becomes extremely difficult to detect, especially if a hard (crisp, defuzzified) label rather than a set of fuzzy class labels must be provided as the output product. (a) Sublette, WY, Rangeland, 42° 51ʹ 37” N, 109° 43ʹ 7” W. Copyright Ralph Maughan, Idaho State Univ. Reproduced with permission of the author. Acquisition date: 6/16/2011. [Online]. Available: http://www.panoramio.com (accessed on 24 February 2013). (b) Twin Falls, ID, Ripening cheatgrass infestation, 42° 23ʹ 52” N, 114° 21ʹ 9” W. Copyright Ralph Maughan, Idaho State Univ. Reproduced with permission of the author. Acquisition date: April 2010? [Online]. Available: http://www.panoramio.com (accessed on 24 February 2013). (c) Overton, NV, 36° 25ʹ 42” N, 114° 27ʹ 21” W. Copyright Ralph Maughan, Idaho State Univ. Reproduced with permission of the author. Acquisition date: 2/11/2009. [Online]. Available: http://www.panoramio.com (accessed on 24 February 2013). (d) San Juan, UT, 37° 16ʹ 43” N, 109° 40ʹ 27” W. Copyright Ralph Maughan, Idaho State Univ. Reproduced with permission of the author. Acquisition date: 3/4/2009. [Online]. Available: http://www.panoramio.com (accessed on 24 February 2013). (e) Springerville, AZ, Volcanic Field, 34° 15ʹ 6” N, 109° 21ʹ 9” W. Copyright Ralph Maughan, Idaho State Univ. Reproduced with permission of the author. Acquisition date: 3/3/2009. [Online]. Available: http://www.panoramio.com (accessed on 24 February 2013). (f) Esmeralda, NV, 38° 1ʹ 40” N, 117° 43ʹ 21” W. Copyright Ralph Maughan, Idaho State Univ. Reproduced with permission of the author. Acquisition date: 4/22/2010. [Online]. Available: http://www.panoramio.com (accessed on 24 February 2013).

**Figure 10.**
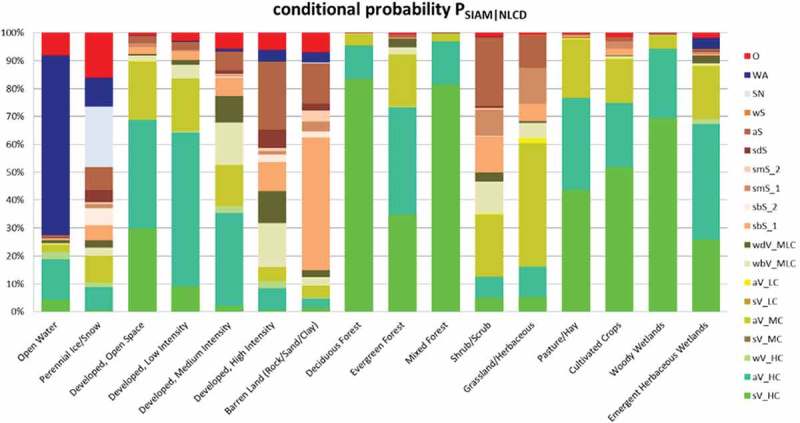
Histogram of the conditional probabilities of the 19 SIAM-WELD 2006 spectral macro-categories (shown as the right column of acronyms, refer to Table 2) at the SIAM intermediate color discretization level, conditioned by one-of-16 NLCD 2006 classes, listed along the horizontal axis. These class-conditional probabilities are derived from Table 4 by normalizing each cell of Table 4 by its column-sum. The same class-conditional probabilities are summarized in text form in Table 6. In this histogram, pseudo-colors associated with the SIAM color types make the interpretation of the histogram columns more intuitive. Green pseudo-colors are associated with the SIAM vegetation-related spectral categories (identified by acronyms of type xV_y on the right column of labels), brown pseudo-colors are selected for the SIAM bare soil-related spectral categories (identified by acronyms of type xS_y on the right column of labels), the pseudo-color blue is chosen for the SIAM spectral category named “*Water or Shadow*” (WA), the light blue pseudo-color is linked to the SIAM spectral category named snow (SN), etc. As a consequence, the column of the USGS NLCD class “*Open Water*” is expected to look blue, columns of the USGS NLCD vegetation-related classes are expected to look green, etc.

**Figure 11.**
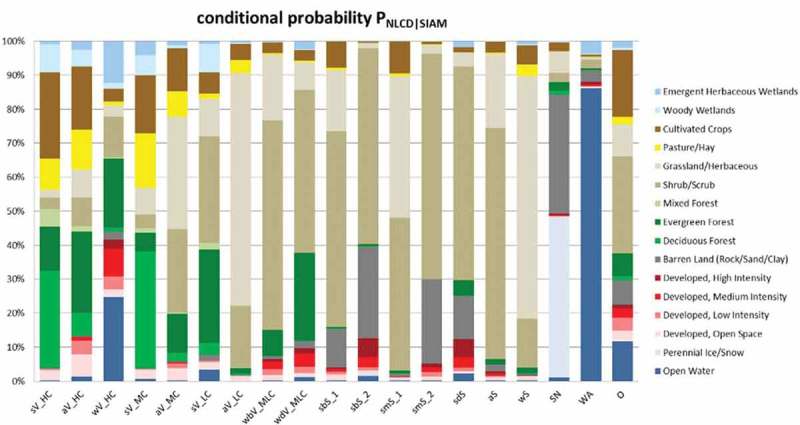
Histogram of the conditional probabilities of the USGS NLCD 2006 map’s 16 LC classes (shown as the right column of class names) conditioned by one-of-19 SIAM-WELD 2006 spectral macro-categories, listed along the horizontal axis as acronyms, refer to Table 2, at the SIAM intermediate color discretization level. This histogram is derived from Table 4 by normalizing each cell of Table 4 by its row-sum. The same class-conditional probabilities are summarized in text form in Table 7. In this histogram, pseudo-colors associated with the USGS NLCD classes make the interpretation of the histogram columns more intuitive. Green pseudo-colors are associated with vegetation NLCD classes, brown pseudo-colors are selected for bare soil NLCD classes, the pseudo-color blue is chosen for the USGS NLCD class “*Open Water,*” the light blue pseudo-color is linked to the USGS NLCD class “*Perennial Ice/Snow,*” etc. As a consequence, the column corresponding to the SIAM spectral category “*Water or Shadow*” (WA) is expected to look blue, column corresponding to the SIAM vegetation-related spectral categories, identified by acronyms of type xV_y located along the horizontal axis, are expected to look green, etc.

**Figure 12.**
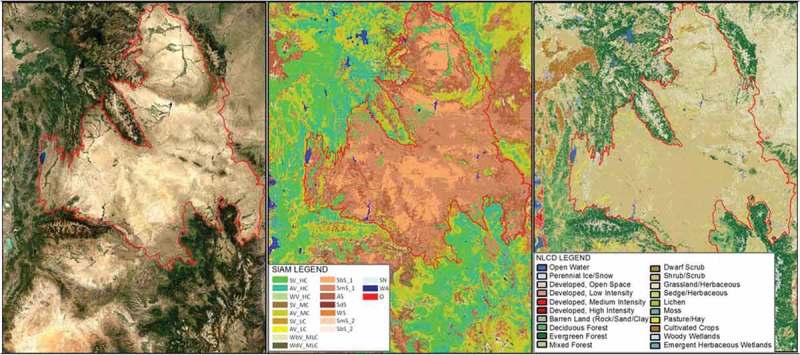
Wyoming Basin ecoregion, as part of the “North American deserts” level 1 ecoregion 10.1.4. *Left*: WELD 2006 tile (true color). *Middle*: SIAM test map of the WELD 2006 tile shown at left, with 19 spectral macro-categories at the intermediate color discretization level. *Right*: NLCD 2006 reference map, featuring 16 LC classes. In these three images, the boundary of the Wyoming Basin ecoregion is overlaid in red. The desertic Wyoming Basin ecoregion is classified as predominantly “*Scrub/Shrub*” (SS) and “*Grassland/Herbaceous*” (GH) in the USGS NLCD 2006 reference map (refer to Table 1), and predominantly as bare soil (sbS_1, SmS_1, aS) in the SIAM-WELD 2006 test map (refer to Table 2). This phenomenon of comprehensive “semantic mismatch” between the USGS NLCD 2006 and SIAM-WELD 2006 thematic maps is explained thoroughly in Chapter 4.3.

**Figure 13.**
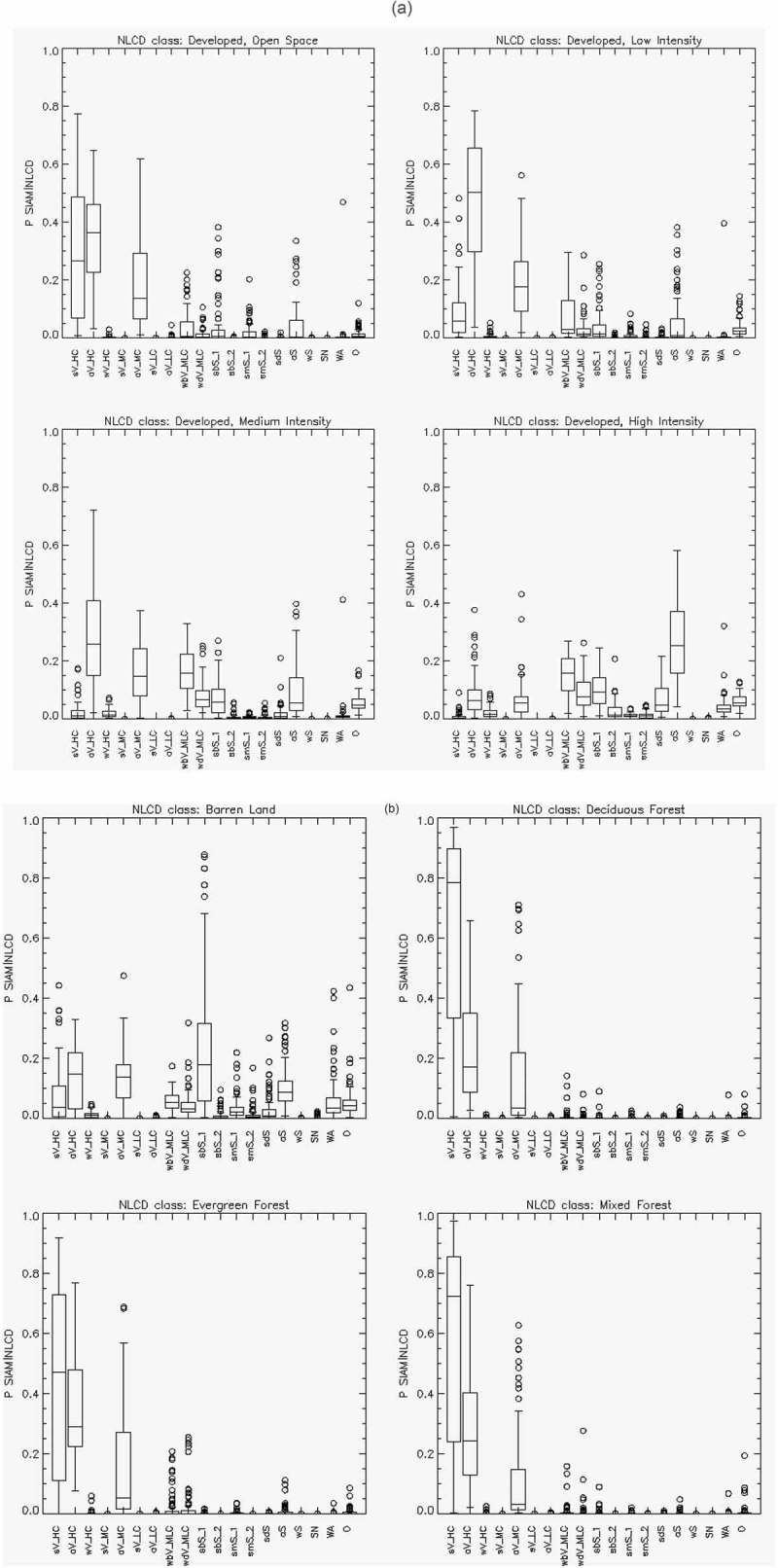
(a)—(d). Reference USGS NLCD class-specific box-and-whisker diagrams, identified by index *r* = 1, …, *RC* = 16, of the USGS NLCD class-conditional probabilities *p*(SIAM-WELD*_er, t_ |* NLCD*_er, r_*), with *t* = 1, …, *TC *= 19, collected across ecoregions *er = *1, …, *ER* = 86. The 19 spectral categories of the SIAM-WELD test map, identified by their acronyms (refer to Table 2), are distributed along the x axis of each NLCD class-specific diagram. Each of the 19 boxes in a box-and-whisker diagram extends from the 25th to the 75^th^ percentile, with a horizontal line to represent the median (50^th^ percentile) of the distribution. The whiskers extend to the maximum or minimum value of the data set, or to 1.5 times the interquantile range, whichever comes first. If there is data beyond this range, it is represented by open circles.

**Figure 13.**
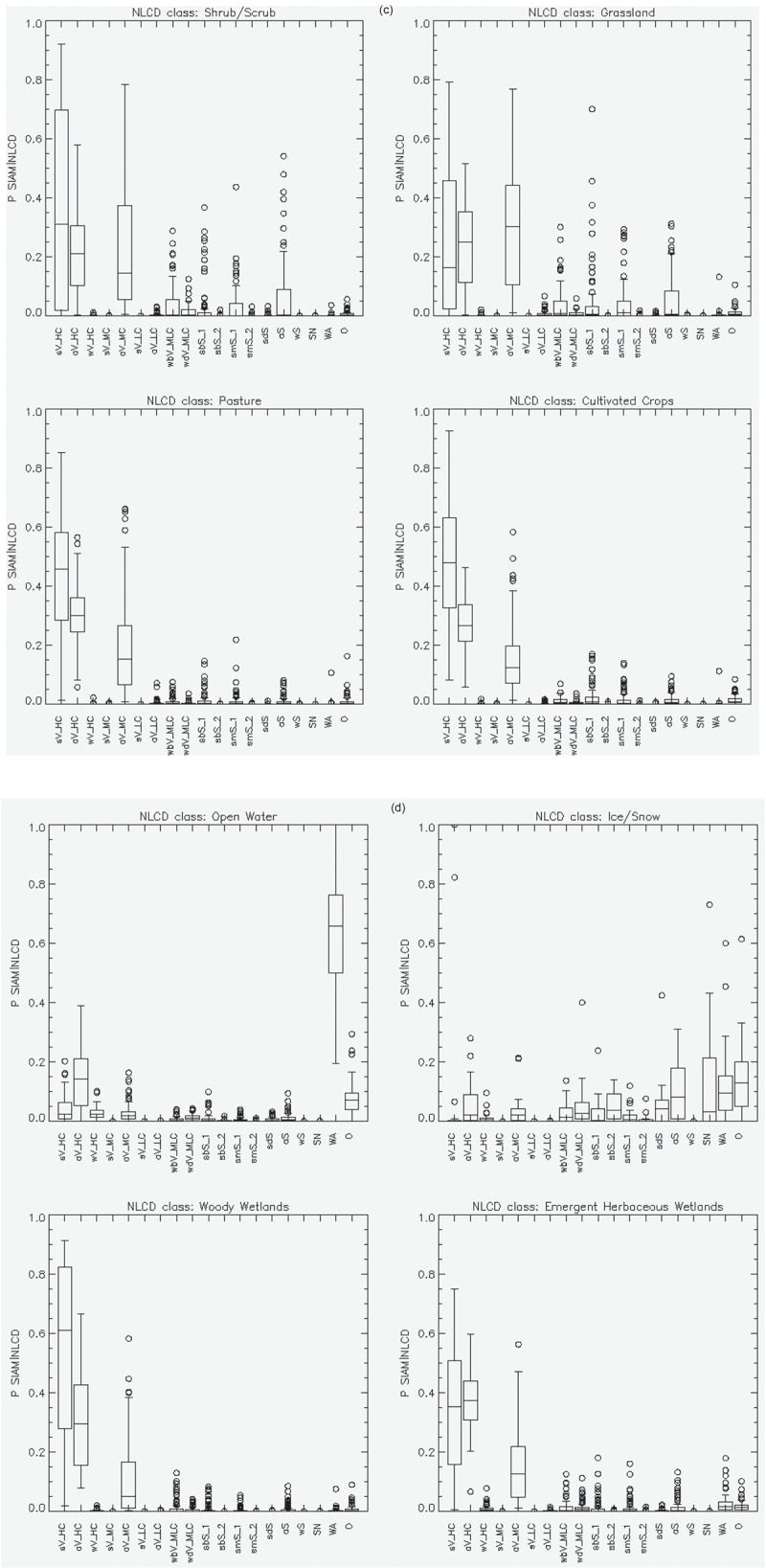
Continued.

The rest of the present Part 2 is organized as follows. Chapter 2 describes materials including the SIAM computer program, the time-series of annual WELD image composites, the reference USGS NLCD 2006 map and the EPA Level III ecoregion map of North America. Methods, specifically, an original protocol to compare without sampling the test SIAM-WELD map and the reference USGS NLCD 2006 map of the CONUS, whose map legends do not coincide and must be harmonized (reconciled, associated, translated) (Ahlqvist, 2005), is proposed in Chapter 3. Experimental results are presented in Chapter 4 and discussed in Chapter 5. Conclusions are reported in Chapter 6.

## 2. Materials

Presented in the RS literature, four alternative implementations of a prior knowledge-based decision tree for static MS reflectance space hyperpolyhedralization into static color names were compared for model selection. (i) The year 2006 SIAM decision tree presented in Baraldi et al. (2006). (ii) The static decision tree for Spectral Classification of surface reflectance signatures (SPECL) proposed by Dorigo et al. (2009), see Table 4 in the Part 1 of this paper, and implemented by the Atmospheric/Topographic Correction for Satellite Imagery (ATCOR) commercial software product (Richter & Schläpfer, 2012a, 2012b). (iii) The static decision tree for Single-Date Classification (SDC), proposed by Simonetti et al. (2015). (iv) The Canada Centre for Remote Sensing (CCRS) spectral decision tree is shown in Figure 17 of the Part 1 (Luo et al., 2008). Whereas the SDC, SPECL, and CCRS decision trees declare their applicability to Landsat images exclusively, SIAM claims its scalability to MS imaging sensors featuring different spectral resolution specifications; see Table 1 in the Part 1. Moreover, the SIAM decision tree outperforms its counterparts in terms of spectral quantization capability, parameterized by the total number of detected color names, equal to 96 for the 7-band Landsat-like SIAM (L-SIAM) subsystem, see Table 1 in the Part 1, versus 13, 19, and 7 color names detected in Landsat images by the SDC, SPECL (see Tables 4 in the Part 1) and CCRS (see Figure 17 in the Part 1) decision trees, respectively. To explain their broad differences in terms of number of detected color names and scalability to MS imaging sensors whose spectral and spatial resolution specifications can vary, the four static spectral decision trees of interest were compared at the level of understanding of spectral information/knowledge representation (Marr, 1982), irrespective of the implementation of the decision rule set (structural knowledge in the decision tree) and of the order of presentation of decision rules (procedural knowledge in the decision tree).

To investigate the scalability of an a priori knowledge-based spectral decision tree to varying MS imaging sensor specifications, we started observing that, given a partition of a MS color space, ℜ^MS^, into a discrete and finite vocabulary (codebook) of hyperpolyhedra equivalent to color names (codewords), identified as {1, ColorVocabularyCardinality}, for any spatial unit x, either (0D) pixel, (1D) line or (2D) polygon defined according to the Open Geospatial Consortium (OGC) nomenclature (OGC, 2015) and featuring a numeric ColorValue(x) ∈ ℜ^MS^, the photometric attribute of spatial unit x can be assigned with a categorical ColorName* ∈ {1, ColorVocabularyCardinality}, such that membership m(ColorValue(x)| ColorName*) = 1, see Equation (3) in the Part 1 of this paper. In practice, when spatial unit x is (0D) pixel, then any prior knowledge-based spectral decision tree for color naming can work at the sensor spatial resolution whatever it is, that is, it can work pixel-based irrespective of the spatial resolution of the imaging sensor.

Because they are independent of the spatial resolution of the imaging sensor, static decision trees for color naming depend on spectral resolution specifications exclusively. Inter-sensor differences in spectral resolution can vary from minor differences in a band-specific sensitivity curve to the major lack of a whole spectral channel. To gain robustness to changes in spectral resolution specifications, the necessary not sufficient pre-condition for spectral rules is to infer “strong” (robust and reliable) conjectures based on the redundant convergence of multiple independent sources of spectral evidence, each of which is individually “weak.” This rationale is alternative to, for example, pruning of redundant processing elements in distributed processing systems such as multi-layer perceptrons (Bishop, 1995; Cherkassky & Mulier, 1998). If this diagnosis holds true, that is, redundancy of spectral evidence is a value-added of spectral rules to scale to varying spectral resolutions, then information redundancy of a spectral if-then rule is expected to increase monotonically with the collection of independent premises.

In a MS reflectance (hyper)cube, any target family of LC class-specific spectral signatures is a multivariate (hyper)polyhedron (envelope, distribution, manifold). Unfortunately, MS reflectance space hyperpolyhedra for color naming are difficult to think of and impossible to visualize when the MS data space dimensionality is superior to three, see Figure 7 in the Part 1 of this paper. Like a vector quantity has two characteristics, a magnitude and a direction, any LC class-specific MS manifold is characterized by a multivariate shape and a multivariate intensity information component; see Figure 2. Hence, spectral information redundancy required to gain robustness to changes in spectral resolution specifications can regard the modelling of both the MS shape and MS intensity information components of a target MS hyperpolyhedron. Among the spectral decision trees being compared, only the SIAM decision tree adopts two different sets of spectral rules to model the MS shape and the MS intensity as two independent spectral information components of a target manifold of MS signatures. On the contrary, in the SDC, SPECL and CCRS decision trees MS shape and MS intensity properties are modeled jointly, which negatively affects principles of modularity, regularity, and hierarchy required by scalable systems (Lipson, 2007). For example, a typical SDC spectral rule applied to a Landsat pixel vector, radiometrically calibrated into a TOARF value in range [0, 1] in each Landsat band 1 to 6, is

$$If\;NDVI\;<\;0.5\;and\;NIR\;\left(\;=\;Landsat\;band\;4\right)\geq 0.15\;then\;do\;something\;else\;do\;otherwise.$$

In this spectral decision rule, the normalized difference vegetation index, NDVI = (NIR—Red)/(NIR + Red), where NIR = Landsat band 4 and Red = Landsat band 3, is a well-known spectral index, whose unbounded version is the band ratio NIR/Red. Noteworthy, band ratios are scalar spectral indexes widely employed in the SPECL decision tree, see Table 4 in the Part 1, and in the CCRS decision tree shown in Figures 17 of the Part 1 (Luo et al., 2008). Any scalar spectral index, either normalized band difference or band ratio, is conceptually equivalent to the slope of a tangent to the spectral signature in one point. This spectral slope is a MS shape descriptor independent of the MS intensity, that is, infinite functions with different intensity values can feature the same tangent value in one point. Although appealing due to its conceptual and numerical simplicity (Liang, 2004), any scalar spectral index is unable per se to represent either the multivariate shape information or the multivariate intensity information component of an MS signature (Baraldi, 2017). Intuitively a scalar spectral index causes a dramatic N-to-1 loss in spectral resolution by reducing an N-channel MS signature to a univariate (scalar) value, corresponding in the (2D) image-plane to a panchromatic (one-channel) image. No photointerpreter whose objective is a one-class LC classification, for example, vegetation detection, would typically consider a panchromatic image made of a univariate spectral index, for example, NDVI, either as informative as an MS image or informative enough to be mapped onto a binary map, for example, vegetation/non-vegetation, where a crisp thresholding criterion is expected to be successful enough to accomplish binary target/no-target discrimination with high accuracy at large spatial extent, different from toy problems. In general, no univariate or multivariate spectral index is representative of the multivariate shape and multivariate intensity information components of an MS manifold; see Figure 2. This obvious, but not trivial observation explains why, in spectral pattern recognition applications, lossy scalar spectral indexes are ever-increasing in number and variety in the endless search for yet-another scalar spectral index, supposedly more informative (Baraldi et al., 2010a, 2010b; Liang, 2004). In the SDC rule reported above, the first spectral term, NDVI < 0.5, constrains per se the multivariate shape of the target MS hyperpolyhedron independent of multivariate intensity; it is employed in logical combination with a second spectral term, where a MS intensity value is restrained as NIR ≥ 0.15, which constrains per se the multivariate intensity of the target MS hyperpolyhedron independent of multivariate shape. The conclusion is that, unlike the SIAM decision rule set, neither the SDC nor the SPECL nor the CCRS decision tree decomposes a target MS hyperpolyhedron, equivalent to a color name, into its multivariate shape and multivariate intensity information components to make each information component easier to be investigated by multivariate data analysis according to principles of modularity, regularity and hierarchy typical of scalable systems (Lipson, 2007). In each of its two independent sets of spectral rules for MS shape and MS intensity modelling SIAM pursues redundancy of spectral terms as a necessary condition to accomplish scalability to changes in the sensor spectral resolution specifications. Possible combinations of these two independent sets of spectral rules make the SIAM decision tree implementations, starting from that proposed in pseudo-code in Baraldi et al. (2006), capable of representing the multivariate shape and multivariate intensity information components of a target MS hyperpolyhedron, neither necessarily convex nor connected, as a converging combination of independent functions whose individual terms are input with 1- to *N*-variate variables, with *N* equal to the total number of spectral channels. Multivariate data statistics are known to be more informative than a sequence of univariate data statistics. For example, maximum likelihood data classification, accounting for multivariate data correlation and variance (covariance), is typically more accurate than parallelepiped data classification whose rectangular decision regions, equivalent to a concatenation of univariate data constraints, poorly fit multivariate data in the presence of bivariate cross-correlation (Lillesand & Kiefer, 1979). In the RS common practice, thanks to its spectral redundancy of multivariate data statistics, the “master” 7-band Landsat-like SIAM (L-SIAM) decision tree can be down-scaled to cope with “slave” MS imaging sensors whose spectral resolution is inferior to but overlaps with Landsat’s; see Table 1 in the Part 1 of this paper (Baraldi et al., 2010a, 2010b).

Moving from this decision-tree models comparison, these authors concluded that the SIAM’s peculiar design in modeling MS hyperpolyhedra and the SIAM implementation complexity/redundancy, superior to that of its alternative decision trees in terms of number of rules and number of terms (premises) per rule, appeared sufficient to justify the SIAM claims of, first, a finer spectral quantization capability and, second, a superior spectral scalability to changes in sensor specifications in comparison with its alternative SDC, SPECL, and CCRS decision tree implementations. Based on these conclusions, an off-the-shelf SIAM application software was selected and considered worth of a GEO-CEOS stage 4 *Val* in compliance with the GEO-CEOS QA4EO *Val* requirements; refer to previous Chapter 1.

To pursue a GEO-CEOS stage 4 *Val* of the SIAM computer program, the 30 m resolution USGS NLCD 2006 map was selected as reference LC map at the CONUS spatial extent. When this experimental work was conducted the USGS NLCD 2006 map was the most recent release of the U.S. NLCD map series developed by the USGS EDC (Vogelmann et al., 1998, 2001; Wickham et al., 2010, 2013; Xian & Homer, 2010; EPA, 2007; Homer et al., 2004); see Figure 4. By now, the U.S. NLCD map series comprises the USGS NLCD 1992, 2001, 2001 Version 2.0, 2006 (released in 2011) and 2011 (released in 2015) editions. The timeliness from EO image collection to NLCD product delivery, which includes information layers such as tree cover fraction and impervious fraction, has steadily decreased from the about 5 years of the initial NLCD product. Made available for public access in a provisional version in February 2011, the USGS NLCD 2006 map was “based primarily on the unsupervised classification” of Landsat-5 Thematic Mapper (TM) and “Landsat-7 Enhanced Thematic Mapper (ETM)+ images acquired in circa 2006” (Xian & Homer, 2010). It has been released as a 30 m resolution raster product in the Albers Equal Area projection, which is the cartographic projection reference standard for continental scale cartography produced by U.S. agencies. Its legend consists of 16 LC classes defined according to the Level II LC classification system; refer to Table 1 (EPA, 2007). Validation of the USGS NLCD 2006 map provided an overall accuracy (OA) of 78%, which increased to 84% when the 16 LC classes were aggregated into 9 LC classes (Stehman, Wickham, Wade, & Smith, 2008; Wickham et al., 2010, 2013). Noteworthy, these 9 LC classes are conceptually equivalent to an “augmented” 3-level 9-class FAO LCCS-DP taxonomy; refer to previous Chapter 1. The validated USGS NLCD 2006 map’s OA values of 78% and 84% with, respectively, a 16 and a 9 LC class legend can be considered state of the art. For example, these OA estimates are superior to OAs featured by national-scale maps recently generated by pixel-based random forest classifiers from monthly WELD composites, whose OA is 65%–67% using 22 detailed classes and 72%–74% using 12 aggregated national classes (Wessels et al., 2016). In general, renowned experts in Geographical Information Science (GIScience) suggest that “the widely used target accuracy of 85% may often be inappropriate and that the approach to accuracy assessment adopted commonly in RS can be pessimistically biased” (Foody, 2006, 2016).

Based on these observations, we considered the USGS NLCD 2006 map’s official OA estimate of 84% realistic and state of the art at the U.S. national scale when the 3-level 9-class “augmented” FAO LCCS-DP legend is adopted. We concluded that the reference USGS NLCD 2006 map was eligible for use in a GEO-CEOS Stage 4 *Val* of the SIAM application software whose output color map legend had to be reconciled with the “augmented” 3-level 9-class FAO LCCS-DP taxonomy of the reference USGS NLCD 2006 map and of a target ESA EO Level 2 product; refer to previous Chapter 1. When a test SIAM-WELD map and a reference USGS NLCD map share the same 30 m spatial resolution and spatial extent, then they can be compared wall-to-wall without sampling. Because no conventional sampling-theory procedure is employed (Lunetta & Elvidge, 1999), a wall-to-wall OA(Test SIAM-WELD; Reference NLCD) estimate ∈ [0, 100%] is provided with a confidence interval (degree of uncertainty in measurement), ± δ ≥ 0, considered mandatory by the GEO-CEOS QA4EO *Val* guidelines, equal to ± δ = 0%.

From a statistic standpoint, the aforementioned experimental work specifications imply the following. Let us identify with OA(Test SIAM-WELD; “Ultimate” GroundTruth) ∈ [0, 100%] = 100%—Mismatch(Test SIAM-WELD; “Ultimate” GroundTruth) the OA of an EO data-derived SIAM test map with respect to an “ultimate” (ideal) ground truth and with OA(Test SIAM-WELD; Reference NLCD) ± 0% = 100%—Mismatch(Test SIAM-WELD; Reference NLCD) ± 0% the overall degree of agreement provided with its confidence interval of a test SIAM-WELD map compared wall-to-wall without sampling with a reference USGS NLCD map at the same spatial resolution and spatial extent. It is known that (Stehman et al., 2008; Wickham et al., 2010, 2013)

OA(Reference NLCD 2006, “augmented” 3-level 9-class FAO LCCS-DP; “Ultimate” GroundTruth 2006, “augmented” 3-level 9-class FAO LCCS-DP) = 84% = 100% - Mismatch(Reference NLCD 2006, “augmented” 3-level 9-class FAO LCCS-DP; “Ultimate” GroundTruth 2006, “augmented” 3-level 9-class FAO LCCS-DP) = 100% - 16%.

Similarly (Stehman et al., 2008; Wickham et al., 2010, 2013),

OA(Reference NLCD 2006, NLCD 16 classes; “Ultimate” GroundTruth 2006, NLCD 16 classes) = 78% = 100% - Mismatch(Reference NLCD 2006, NLCD 16 classes; “Ultimate” GroundTruth 2006, NLCD 16 classes) = 100% - 22%.

Based on the superposition principle, see Figure 5, it is possible to write

OA(Test SIAM-WELD; “Ultimate” GroundTruth) ∈ [0, 100%] = {OA(Reference NLCD; “Ultimate” GroundTruth) ± Mismatch(Test SIAM-WELD; Reference NLCD) ± 0%} ∈ [Worst Case, Best Case], where Worst Case = max{0%, Lower Bound} and Best Case = min{100%, Upper Bound}, with Lower Bound ≤ Upper Bound ∈ [0%, 100%].

When the “Ultimate” GroundTruth adopts an “augmented” 9-class LCCS-DP legend, then the aforementioned Lower and Upper Bounds become (Stehman et al., 2008; Wickham et al., 2010, 2013)

Lower Bound = [OA(Reference NLCD 2006, “augmented” 3-level 9-class FAO LCCS-DP; “Ultimate” GroundTruth 2006, “augmented” 3-level 9-class FAO LCCS-DP) – Mismatch(Test SIAM-WELD; Reference NLCD 2006, “augmented” 3-level 9-class FAO LCCS-DP) ± 0%] = [100% – Mismatch(Reference NLCD 2006, “augmented” 3-level 9-class FAO LCCS-DP; “Ultimate” GroundTruth 2006, “augmented” 3-level 9-class FAO LCCS-DP) – (100% - OA(Test SIAM-WELD; Reference NLCD 2006, “augmented” 3-level 9-class FAO LCCS-DP) ± 0%)] = [OA(Test SIAM-WELD; Reference NLCD 2006, “augmented” 3-level 9-class FAO LCCS-DP) ± 0% - Mismatch(Reference NLCD 2006, “augmented” 3-level 9-class FAO LCCS-DP; “Ultimate” GroundTruth 2006, “augmented” 3-level 9-class FAO LCCS-DP)] = [OA(Test SIAM-WELD; Reference NLCD 2006, “augmented” 3-level 9-class FAO LCCS-DP) ± 0% - 16%],

and (Stehman et al., 2008; Wickham et al., 2010, 2013)

Upper Bound = [OA(Reference NLCD 2006, “augmented” 3-level 9-class FAO LCCS-DP; “Ultimate” GroundTruth 2006, “augmented” 3-level 9-class FAO LCCS-DP) + Mismatch(Test SIAM-WELD; Reference NLCD 2006, “augmented” 3-level 9-class FAO LCCS-DP) ± 0%] = [84% + (100% - OA(Test SIAM-WELD; Reference NLCD 2006, “augmented” 3-level 9-class FAO LCCS-DP) ± 0%)] = [184% - OA(Test SIAM-WELD; Reference NLCD 2006, “augmented” 3-level 9-class FAO LCCS-DP) ± 0%].

To recapitulate, when the “Ultimate” GroundTruth adopts an “augmented” 3-level 9-class FAO LCCS-DP legend, it is expected that (Stehman et al., 2008; Wickham et al., 2010, 2013)

OA(Test SIAM-WELD; “Ultimate” GroundTruth 2006, “augmented” 3-level 9-class FAO LCCS-DP) ∈ [max{0%, Lower Bound}, min{100%, Upper Bound}] = [max{0%, OA(Test SIAM-WELD; Reference NLCD 2006, “augmented” 3-level 9-class FAO LCCS-DP) ± 0% - 16%}, min{100%, 184% - OA(Test SIAM-WELD; Reference NLCD 2006, “augmented” 3-level 9-class FAO LCCS-DP) ± 0%}]. (1)

Similarly, when the “Ultimate” GroundTruth adopts a 16-class NLCD legend, then it is expected that (Stehman et al., 2008; Wickham et al., 2010, 2013)

OA(Test SIAM-WELD; “Ultimate” GroundTruth 2006, NLCD 16 classes) ∈ [max{0%, Lower Bound}, min{100%, Upper Bound}] = [max{0%, OA(Test SIAM-WELD; Reference NLCD 2006, NLCD 16 classes) ± 0% - 22%}, min{100%, 178% - OA(Test SIAM-WELD; Reference NLCD 2006, NLCD 16 classes) ± 0%}]. (2)

Equations (1) and (2) are useful because, first, they highlight the undisputable fact that per se the reference USGS NLCD 2006 map is not a “ground truth” for the test SIAM-WELD map, but only a reference baseline for comparison purposes. Second, they support the validity of this experimental project by showing that a summary statistic OA(Test SIAM-WELD; “Ultimate” GroundTruth 2006) can be inferred from an estimated OA(Test SIAM-WELD; Reference NLCD 2006) ± 0% known that OA(Reference NLCD 2006; “Ultimate” GroundTruth 2006) is equal to 84% or 78% when the NLCD 2006 map and the “Ultimate” GroundTruth adopt an “augmented” 3-level 9-class FAO LCCS-DP legend or the 16-class NLCD legend, respectively.

Supported by NASA and distributed by the USGS EDC (WELD, 2016), the annual WELD image composites for years 2006, 2007, 2008, and 2009 were selected as a large-scale EO image time-series radiometrically calibrated into TOARF values as required by a GEO-CEOS stage 4 *Val* of the SIAM application software in comparison with the reference USGS NLCD 2006 map; see Figure 4. Each annual WELD image composite consists of approximately 8,000 Landsat-5/7 image acquisitions per year over the CONUS, starting from year 2003 to year 2012. The current WELD processing workflow requires as input Landsat sensor series L1T images with cloud cover ≤ 20%. The WELD composite of the CONUS encompasses 501 fixed location tiles defined in the Albers Equal Area projection. Each tile is 5000 × 5000 pixels in size, equal to 150 × 150 km (Homer et al., 2004). The Landsat sensor series L1T image geolocation error in the CONUS, including areas with substantial terrain relief, is less than 30 m (< 1 pixel) (Lee, Storey, Choate, & Hayes, 2004). The most recent Landsat data radiometric *Cal* expertise is employed in the WELD workflow to ensure harmonization and interoperability of multi-sensor Landsat image time-series, with a 5% absolute reflective band *Cal* uncertainty (Markham & Helder, 2012), in agreement with the GEO-CEOS QA4EO *Cal/Val* requirements (GEO-CEOS, 2010). Figure 6 shows the annual WELD 2006 image composite over the CONUS, where TOARF values are depicted in true colors and linearly stretched for visualization purposes, with the WELD tiling scheme overlaid in white.

To account for typical non-stationary geospatial statistics, an inter-map statistical comparison on a stratified (masked) basis should be accomplished at a local spatial extent, where strata convey some geospatial criteria of land surface information invariance. The USGS NLCD 2006 reference map was partitioned into Level III ecoregions of North America collected from the EPA (EPA, 2013). There are 86 ecoregions across the CONUS, each ecoregion featuring similar ecological and climatic characteristics (Griffith & Omernik, 2009). Distributed as vector data, the EPA Level III ecoregions were rasterized to 30 m resolution in the Albers Equal Area projection. Figure 4 shows the USGS NLCD 2006 map with boundaries of ecoregions overlaid in black.

## 3. Methods

A wall-to-wall comparison without sampling between the test SIAM-WELD map time-series and the reference USGS NLCD 2006 map, sharing the same 30 m spatial resolution at the CONUS spatial extent, but whose legends A = VocabularyOfColorNames (see Table 2) and B = LegendOfObjectClassNames (see Table 1) do not coincide and must be harmonized, was designed and implemented for a GEO-CEOS stage 4 *Val* purpose. These working hypotheses differ from thematic map accuracy assessment protocols adopted by the large majority of the RS community, typically based on an either random (probabilistic) or non-random sampling in combination with a square and sorted confusion matrix, CMTRX. A CMTRX is defined as a special case of a two-way contingency table (bivariate frequency table), BIVRFTAB = FrequencyCount(A × B), where A × B is a 2-fold Cartesian product between two univariate categorical variables A and B of the same population and where A ≠ B in general (Kuzera & Pontius, 2008; Pontius & Connors, 2006; Pontius & Millones, 2011). In particular, a CMTRX is square and sorted because the test and reference categorical variables A and B of the same population are required to be the same, to let the main diagonal guide the interpretation process; refer to the Part 1, Chapter 2.

**Table 2.** List of the 19 spectral macro-categories generated from the aggregation by the independent human expert of the SIAM’s 48 spectral categories originally detected at the intermediate level of color quantization. The “*Water or Shadow*” (WA) spectral macro-category results from the aggregation of six original SIAM categories, the “*Snow*” (SN) spectral macro-category from two and the spectral macro-category “*Others*” (O) from the aggregation of 24 original spectral categories covering disturbances typically minimized or removed in an annual composite (clouds, smoke plumes, fire fronts, etc.) as well as the original spectral category “*Unknowns*.” Hence, (19–3) + 6 + 2 + 24 = 48, which is the SIAM’s intermediate color discretization level. In the proposed names of spectral macro-categories, acronym Near Infra-Red (NIR) indicates Landsat TM/ETM+ band 4 (0.9 μm) and acronym Medium Infra-Red (MIR) indicates Landsat TM/ETM+ band 5 (1.6 μm). The right column instantiates a possible binary relationship R: A ⇒ C ⊆ A × C from set A = 19-class SIAM legend to set C = 2-level 4-class Dichotomous Phase (DP) taxonomy of the Food and Agriculture Organization of the United Nations (FAO)—Land Cover Classification System (LCCS) (Di Gregorio & Jansen, 2000), refer to Figure 1

SIAM, Intermediate discretization level (featuring 48 spectral categories) reassembled into 19 spectral macro-categories				LCCS-DP, level 1: A = Veg, B = Non-veg, and level 2: 1 = Terrestrial, 2 = Aquatic
ID	Abbreviation	OR-Aggregations	Spectral macro-category name	ID
1	sV_HC	1	Strong evidence vegetation, high canopy cover	A1—Vegetated terrestrial
2	aV_HC	1	Average evidence vegetation, high canopy cover	A1
3	wV_HC	1	Weak evidence vegetation, high canopy cover	A1
4	sV_MC	1	Strong evidence vegetation, medium canopy cover	A1
5	aV_MC	1	Average evidence vegetation, medium canopy cover	A1
6	sV_LC	1	Strong evidence vegetation, low canopy cover	A1
7	aV_LC	1	Average evidence vegetation, low canopy cover	A1
8	wbV_MLC	1	Weak evidence bright vegetation, medium or low canopy cover	A1
9	wdV_MLC	1	Weak evidence dark vegetation, medium or low canopy cover	A1/A2—Vegetated aquatic
10	sbS_1	1	Strong evidence bright soil AND NIR ≤ MIR	B3—Non-vegetated terrestrial
11	sbS_2	1	Strong evidence bright soil AND NIR > MIR	B3
12	smS_1	1	Strong evidence medium soil AND NIR ≤ MIR	B3
13	smS_2	1	Strong evidence medium soil AND NIR > MIR	B3
14	sdS	1	Strong evidence dark soil	B3
15	aS	1	Average evidence soil	B3
16	wS	1	Weak evidence soil	B3
17	SN	2	Snow	B4—Non-vegetated aquatic
18	WA	6	Water or Shadow	B4
19	O	24	Others	B—Non-vegetated
	**TOT.**	48		

In Baraldi, Boschetti, and Humber (2014), a crisp thematic map assessment protocol was proposed based on: (i) a probability sampling strategy, (ii) a pair of test and reference thematic map legends A and B that may differ, (iii) a crisp overlapping area matrix, OAMTRX = FrequencyCount(A × B), defined as a BIVRFTAB instantiation estimated from a geospatial population with or without sampling, (Beauchemin & Thomson, 1997; Ortiz & Oliver, 2006), whose spatial unit x is (0D) pixel, (iv) a set of thematic quantitative quality indicators (Q^2^Is), TQ^2^Is, extracted from the OAMTRX and (v) a set of spatial Q^2^Is (SQ^2^Is) extracted from sub-symbolic image-objects (image-segments) in the multi-level map domain, where image-objects are either (0D) pixels, (1D) lines or (2D) polygons according to the OGC nomenclature (OGC, 2015). Whereas the construction of an OAMTRX is straightforward and non-controversial when categorical labels of sampling units are crisp (hard), the method to construct an OAMTRX when categorical labels are soft (fuzzy) is not obvious at all; for example, refer to (Kuzera & Pontius, 2008). Hence, those authors focused on crisp OAMTRX instances, exclusively.

To accomplish our present working hypotheses, the crisp thematic map probability sampling protocol proposed in (Baraldi et al., 2014) was modified as follows.

- The original hybrid eight-step guideline proposed in the Part 1, Chapter 4 was adopted to streamline the inherently subjective selection by human experts of a binary relationship R: A = VocabularyOfColorNames ⇒ B = LegendOfObjectClassNames ⊆ A × B that guides the interpretation process of a crisp OAMTRX = FrequencyCount(A × B) = FrequencyCount(VocabularyOfColorNames × LegendOfObjectClassNames); see Table 3 in the Part 1 of this paper.
- Given a binary relationship R: A = VocabularyOfColorNames ⇒ B = LegendOfObjectClassNames to guide the interpretation process of a crisp OAMTRX = FrequencyCount(A × B) ≠ R: A ⇒ B ⊆ A × B, a novel formulation CVPAI2(R: A ⇒ B ⊆ A × B) was adopted as a relaxed version of the CVPAI1 formulation proposed in (Baraldi et al., 2014); refer to the Part 1, Chapter 5 and to Figure 18 in the Part 1 of this paper.
- Traditional 30 m resolution Landsat image classifiers are pixel-based because MS color information tends to dominate spatial information in 30 m resolution MS imagery acquired from space where; for example, individual man-made objects, such as individual buildings, roads, or agricultural fields, are typically hard to distinguish. Hence, in the 30 m resolution WELD composites, the most informative planar entity is (0D) pixel, rather than image-object, either (1D) line or (2D) polygon (OGC, 2015). As a consequence, for the sake of simplicity, in the present thematic map comparison image-object-based SQ^2^Is were omitted. Rather, the following pixel-based TQ^2^Is were estimated from the crisp OAMTRX = FrequencyCount(A × B) estimated wall-to-wall with spatial unit x equal to pixel.
  - An OA(OAMTRX = FrequencyCount(A × B)) ± 0% was computed in line with (Baraldi et al., 2014; Pontius & Millones, 2011; Stehman & Czaplewski, 1998). This OA estimate is guided by the binary relationship R: A = VocabularyOfColorNames ⇒ B = LegendOfObjectClassNames identified and community-agreed upon in advance; refer to this text above. In an OAMTRX = FrequencyCount(A × B) estimated from a wall-to-wall inter-map comparison, where no sample data is investigated, any adopted TQ^2^I features a degree of uncertainty in measurement equal to ± 0%; for example, see Equation (1).
  - User’s and producer’s accuracies, computed in (Baraldi et al., 2014; Pontius & Millones, 2011; Stehman & Czaplewski, 1998), were replaced by class-conditional probabilities, *p*(*r* | *t*) of reference class *r* given test class *t* and, vice versa, *p*(*t* | *r*) of test class *t* given reference class *r*, with *r* = *1*, …, *RC*, and *t* = 1, …, *TC*, where *RC* = |B| = b = ObjectClassLegendCardinality and *TC* = |A| = a = ColorVocabularyCardinality are the total numbers of reference and test classes, respectively.

The proposed ensemble of TQ^2^I summary statistics, specifically, CVPAI2(R: A ⇒ B ⊆ A × B), OA(OAMTRX = FrequencyCount(A × B)) and class-conditional probabilities(OAMTRX), is an original minimally dependent and maximally informative (mDMI) set (Si Liu, Hairong Liu, Latecki, Xu, & Lu, 2011; Peng, Long, & Ding, 2005) of outcome Q^2^Is (O-Q^2^Is), to be jointly maximized according to the Pareto formal analysis of multi-objective optimization problems (Boschetti, Flasse, & Brivio, 2004); refer to the Part 1, Chapter 1.

## 4. Validation session

According to the GEO-CEOS *Val* guidelines (GEO-CEOS, 2010; GEO-CEOS WGCV, 2015), *Val* is the process of assessing, by independent means, the quality of an information processing system by means of an mDMI set (Si Liu et al., 2011; Peng et al., 2005) of community-agreed outcome and process (OP) Q^2^Is (OP- Q^2^Is), each one provided with a degree of uncertainty in measurement, ± δ, with δ ≥ 0%.

In the present study, the following definition is adopted: an information processing system can be considered in operating mode (ready-to-go) if it scores “high” in all of its OP-Q^2^I estimates; refer to the Part 1, Chapter 1.

To comply with the GEO-CEOS stage 4 *Val* requirements (GEO-CEOS WGCV, 2015), refer to previous Chapter 1, the SIAM-WELD data mapping process and outcome were validated by a human expert independent of the present authors (refer to Acknowledgments). This independent human expert accomplished the following tasks. (I) Run without user interaction an off-the-shelf SIAM application upon the 30 m resolution annual WELD 2006 to 2009 image composites of the CONUS. (II) Overlap wall-to-wall the test SIAM-WELD annual map time-series with the reference USGS NLCD 2006 map to generate instances of an OAMTRX = FrequencyCount(A × B) (Baraldi et al., 2014). (III) Estimate an mDMI set of OP-Q^2^Is, defined as follows, in agreement with the Part 1, Chapter 1 (Baraldi & Boschetti, 2012a, 2012b; Baraldi & Humber, 2015; Baraldi et al., 2013).

- Product effectiveness. Proposed outcome Q^2^Is (O-Q^2^Is) were the TQ^2^Is presented in Chapter 3: (a) CVPAI2(R: A ⇒ B ⊆ A × B; (b) OA(OAMTRX = FrequencyCount(A × B)), and (c) class-conditional probabilities *p*(*r* | *t*) and p(*t* | *r*) with test class *t* = 1, …, *TC =* |A| = ColorVocabularyCardinality and reference class *r* = *1*, …, *RC =* |B| = ObjectClassLegendCardinality.
- Process efficiency. Proposed process Q^2^Is (P-Q^2^Is) were: (a) computation time and (b) run-time memory occupation.
- Process degree of automation, monotonically decreasing with the number of system’s free-parameters to be user defined.
- Process robustness to changes in the input dataset. For post-classification change/no-change detection (Lunetta & Elvidge, 1999), the SIAM-WELD 2006 to 2009 maps were compared with one another when one year apart.
- Process robustness to changes in input parameters, if any.
- Process scalability, to keep up with changes in users’ needs and sensor specifications.
- Product timeliness, defined as the time between data acquisition and product generation.
- Product costs in manpower and computer power.

For the sake of paper simplicity, the following decisions were undertaken.

- Two-of-three SIAM-WELD 2006 output color maps, specifically, the one featuring 96 color names, equivalent to a fine color granularity, and the one featuring 48 color names, equivalent to an intermediate color granularity, were compared with the 16-class NLCD 2006 map, while the SIAM-WELD 2006 color map featuring 18 color names, equivalent to a coarse color granularity, was ignored, see Table 1 in the Part 1. This implied the following.
  - In the case of a SIAM-WELD 2006 map featuring 96 color names at the SIAM fine color discretization level, an OAMTRX = FrequencyCount(A × B) instance consisted of a test set A = 96 spectral categories as rows and a reference set B = 16 NLCD classes as columns. Because of its excessive size, equal to 96 × 16 cells, this OAMTRX instance cannot be shown in a technical paper. However, it is made available on an anonymous ftp site (SIAM-WELD-NLCD FTP, 2016), and its TQ^2^I summary statistics are reported in the present paper.
  - In the case of a SIAM-WELD 2006 map featuring 48 color names at the SIAM intermediate color discretization level, an OAMTRX = FrequencyCount(A × B) instance consisted of a test set A = 48 spectral categories as rows and a reference set B = 16 NLCD classes as columns. Because of its excessive size, equal to 48 × 16 cells, this OAMTRX instance cannot be shown in a technical paper. Hence, the SIAM ensemble of 48 basic color (BC) names at intermediate color granularity was perceptually (“subjectively”) reassembled into 19 spectral macro-categories, refer to Table 2 (Benavente, Vanrell, & Baldrich, 2008; Berlin & Kay, 1969; Gevers, Gijsenij, Van De Weijer, & Geusebroek, 2012; Griffin, 2006), by the independent human expert who adopted in support the hybrid guideline for binary relationship detection proposed in the Part 1, Chapter 4. This reduced set of 19 spectral macro-categories was constrained to be mutually exclusive and totally exhaustive, in line with the Congalton and Green’s requirements of a classification scheme (Congalton & Green, 1999). Such a grouping of BC names into parent spectral macro-categories scrutinized and agreed upon by a human expert pertains to the inherently equivocal (subjective) domain of *information-as-data-interpretation*; refer to the Part 1, Chapter 4 (Capurro & Hjørland, 2003). Among the 19 spectral macro-categories reassembled by the independent human expert, 16 macro-categories coincided exactly with one BC name in the SIAM set of 48 BC names at intermediate granularity. One-of-19 spectral macro-category, named “*Others*” by the independent human expert, grouped 25-of-48 BC names detected by SIAM at intermediate color granularity. Among these 25-of-48 BC names, one is identified by SIAM as category “*Unknowns*” (outliers), while the remaining 24 BC names are all related to spectral signatures equivalent to “noisy” data (no terrain data), such as spectral signatures typical of LC classes cloud, smoke plume, active fire, and so on, which are typically minimized in an annual WELD image composite according to the known WELD multi-temporal pixel selection policies; refer to previous Chapter 2. As a consequence of grouping the SIAM’s 48 BC names into 19 spectral macro-categories, a simplified OAMTRX instance of reduced size was generated as OAMTRX = FrequencyCount(A^ × B), where a test set A^ = 19 spectral macro-categories was adopted as rows and a reference set B = 16 NLCD classes was adopted as columns. Thanks to its size, reduced to 19 × 16 cells, this OAMTRX instance can be shown, in combination with its estimated TQ^2^I values, in the present paper.
- In agreement with the previous paragraph, the annual SIAM-WELD 2006 to 2009 maps at the SIAM intermediate color discretization level of 48 color names were all reassembled into 19 spectral macro-categories; see Table 2.

### 4.1. Verification of the co-registration requirements for pixel-based inter-map comparison

In the requirements specification of RS projects dealing with per-pixel post-classification change/no-change detection, the required RS image co-registration error is typically < 1 pixel. For example, in (Lunetta & Elvidge, 1999), it is recommended that the root-mean-square (RMS) co-registration error between any pair of two-date imagery should not exceed 0.5 pixels.

In (Dai & Khorram, 1998), simulated misregistration effects are investigated upon multi-temporal Landsat images of North Carolina across four study areas representative of land cover types: forest land, agricultural land, bare soil, and urban/residential area. In these experiments, a registration accuracy < 1/5 of a pixel is considered necessary to achieve a land cover change detection error < 10%. This conclusion is more severe than the one-pixel co-registration constraint typically adopted in most change detection applications.

The annual WELD composites and the USGS NLCD 2006 thematic map were derived from the same sensory dataset of Landsat L1T images acquired by the USGS EDC. It means that the SIAM-WELD 2006 pre-classification maps and the USGS NLCD 2006 reference map were derived from the same sensory dataset. Hence, it is reasonable to assume that the co-registration error between these data-derived maps is negligible.

### 4.2. Inter-annual SIAM-WELD map comparisons for years 2006 to 2009

The consistency across time and space of the annual SIAM-WELD 2006 to 2009 map time-series featuring a map legend of 19 spectral macro-categories was investigated. Based on a priori knowledge of the multi-temporal pixel-based selection criteria adopted by the USGS EDC for the generation of annual WELD composites (refer to Chapter 2) and of the LC/LC change (LCC) dynamics in the real-world CONUS, a small percentage of LCC counts was expected to be detected one year apart at the CONUS spatial extent.

Table 3 shows class-conditional percentages collected at the CONUS spatial extent across the annual WELD image composite time-series for each of the 19 SIAM-WELD spectral macro-categories. The green-as-“*Vegetation*” spectral macro-categories are predominant (refer to the total vegetation statistic reported in Table 3), with an average 79% of the CONUS pixels, followed by MS color names such as brown-as-“*Bare soils or built-up*” (19% on average), followed by the remaining spectral macro-categories that, altogether, account for about 2%. The standard deviation through time of the occurrence of each SIAM-WELD spectral macro-category at the CONUS spatial extent is lower than 1%, with the exception of two vegetation spectral macro-categories (specifically, aV_HC and aV_MC) where a larger variance can be attributed mostly to phenology. If a vegetation-through-time spectral variability due to changes in phenology affects the annual WELD composites then the data-derived SIAM-WELD color quantization maps will be affected by changes in phenology too. This diagnosis was verified as follows. Because of the limited availability of cloud-free Landsat observations at a generic pixel location per year, the Julian day of the year of the observation selected at a given location (pixel) of the annual WELD image composite changes through years (Roy et al., 2010). This is illustrated in Figure 7 where, at any fixed location across a target “ground-truth” area of deciduous forest in a pair of monthly August-November WELD composites, the SIAM spectral labels change significantly, but consistently with the phenological season. The same consideration holds when changes in phenology affect the annual WELD composites. This can explain the “high” intra-vegetation spectral variability observed by the SIAM vegetation-related spectral macro-categories aV_HC and aV_MC in the tested time-series of annual WELD composites for years 2006 to 2009.

Non-stationary spatial phenomena occurring at the CONUS spatial extent in the geospatial physical world can be oversighted by global statistics. To be better captured, spatial non-stationarities require more local statistics, such as class-conditional global statistics described in Table 3.

According to previous Chapter 3, for every pair of one annual SIAM-WELD test map with legend A = 19 spectral macro-categories for year 2006 to year 2009 overlapped at the CONUS spatial extent with a reference USGS NLCD 2006 map with legend B = NLCD 16 classes, the pair of summary statistics CVPAI2(R: A ⇒ B ⊆ A × B) ∈ [0, 1.0] and OA(OAMTRX = FrequencyCount(A × B)) ∈ [0, 1.0] should be maximized jointly. Shown as gray entry-pair cells in Table 4, a binary relationship R: A ⇒ B was identified by the independent human expert, who adopted the hybrid eight-step guideline for identification of a categorical variable-pair relationship proposed in the Part 1, Chapter 4. The binary relationship R: A ⇒ B, selected by the human expert and shown in Table 4, provides a CVPAI2(R: A ⇒ B) = 0.6689, while the OA(OAMTRX = FrequencyCount(A × B) = Table 4) = OA(Test SIAM-WELD 2006, 19 spectral macro-categories; Reference NLCD 2006, NLCD 16 classes) = 96.88% ± 0%. With a binary relationship R: A ⇒ B kept fixed, where CVPAI2(R: A ⇒ B) = 0.6689, the OA(OAMTRX) estimate became equal to 97.02%, 96.69%, and 96.75% for the annual SIAM-WELD map of year 2007 to year 2009 compared with the reference USGS NLCD 2006 map.

### 4.3. Comparison of the SIAM-WELD 2006 and NLCD 2006 thematic maps

The pair of SIAM-WELD 2006 test maps at intermediate and fine color quantization levels, featuring 48 and 96 BC names respectively, were compared wall-to-wall with the USGS NLCD 2006 reference map as described below.

#### 4.3.1. Test case A

The SIAM-WELD 2006 map of the CONUS at the intermediate color discretization level reassembled into 19 spectral macro-categories is shown in Figure 8. The OAMTRX = FrequencyCount(A × B) instance generated from the overlap between the test SIAM-WELD 2006 map with legend A = 19 spectral macro-categories at the CONUS spatial extent with a reference USGS NLCD 2006 map with legend B = NLCD 16 classes is shown in Table 4, where each cell reports a joint probability value *p*(SIAM-WELD_t_, NLCD_r_), *r* = 1, …, *RC *= |B| = 16, refer to Table 2, and *t* = 1, …, *TC *= |A| = 19, refer to Table 1. Gray entry-pair cells identify the binary relationship R: A ⇒ B ⊆ A × B ≠ OAMTRX = FrequencyCount(A × B) chosen by the independent human expert to guide the OAMTRX interpretation process. The distribution of these “correct” entry-pairs shows that every NLCD class overlaps with several discrete color types, with the exceptions of two SIAM-NLCD entry-pairs, specifically, entry-pair [SIAM spectral macro-category, NLCD class] = [MS white-as-”*Snow*” (SN, see Table 2), NLCD class “*Perennial ice/snow*” (PIS, see Table 1)] and entry-pair [SIAM spectral macro-category, NLCD class] = [MS blue-as-“*Water or Shadow*” (WA, see Table 2), NLCD class “*Open water*” (OW, see Table 1)], which are both characterized by a 1–1 matching relation. According to their specific definitions in natural language (refer to Table 1), anthropic NLCD classes, such as “*Developed, Open Space*” (DOS), “*Developed, Low Intensity*” (DLI), “*Developed, Medium Intensity*” (DMI) and “*Developed, High intensity*” (DHI), are a mixture of vegetated surfaces, impervious surfaces and bare soil, in agreement with the popular vegetation-impervious surface-soil model for urban ecosystem analysis (Ridd, 1995). In agreement with their definitions in human language, these NLCD classes overlap exclusively with the SIAM spectral macro-categories related to vegetation or bare soil. The USGS NLCD class “*Barren Land*” (BL, see Table 1) overlaps with all of the SIAM spectral macro-categories related to bare soil. Noteworthy, according to Table 4, the USGS NLCD class BL covers only 1.21% of the CONUS total surface. This is due to the USGS NLCD 2006 definition of class BL (Rock/Sand/Clay), very restrictive with regard to the presence of vegetation, which has to account for less than 15% of total cover. The USGS NLCD definition of class BL means that the USGS NLCD classes “*Shrub/Scrub*” (SS) and “*Grassland/Herbaceous*” (GH, refer to Table 1) may feature a vegetated cover which accounts for 15% of total cover or more. The USGS NLCD forest classes “*Deciduous forest*” (DF), “*Evergreen Forest*” (EF) and “*Mixed forest*” (MF, refer to Table 1) overlap with the SIAM’s high and medium canopy cover-related spectral macro-categories. The USGS NLCD vegetation classes “*Shrub/Scrub*” (SS) and “*Grassland/Herbaceous*” (GH, refer to Table 1) overlap with the SIAM-WELD 2006 medium and low canopy cover-related spectral macro-categories, but, in case of dry or sparse vegetation, also with some of the SIAM-WELD 2006 spectral macro-categories related to bare soil, namely, sbS_1, SmS_1, and aS (refer to Table 2). The overlap between the reference USGS NLCD 2006 vegetation classes SS and GH and the test SIAM-WELD 2006 bare soil spectral macro-categories sbS_1, SmS_1, and aS is the only case of comprehensive (systematic) “semantic mismatch” recorded across the wall-to-wall SIAM-WELD 2006 and NLCD 2006 thematic map pair comparison. Hence, it is worth a deeper analysis in comparison with an “ultimate” ground truth. Reported above in this chapter, the USGS NLCD 2006 definition of class “*Barren Land*” (BL, see Table 1) means that the USGS NLCD vegetation classes SS and GH may feature a vegetated cover that accounts for 15% of total cover or more. Two consequence of these three NLCD class definitions are that, whereas the USGS NLCD class BL covers only 1.21% of the CONUS total surface, the USGS NLCD vegetation classes SS and GH map the near totality of desert areas across the CONUS. Hence, there is a systematic “semantic mismatch” between the USGS NLCD 2006 vegetation classes SS and GH and the SIAM-WELD 2006 bare soil spectral macro-categories across nearly all desert areas of the CONUS. Figure 9 shows real-world examples of geographic locations mapped as vegetation classes “*Scrub/Shrub*” (SS) or “*Grassland/Herbaceous*” (GH) in the USGS NLCD 2006 map (refer to Table 1), while they are mapped predominantly as the bare soil spectral categories sbS_1, SmS_1, and aS in the SIAM-WELD 2006 map (refer to Table 2). For more comments about this systematic case of “conceptual mismatch” between the test SIAM-WELD and reference USGS NLCD 2006 maps, refer to Figure 12.

Additional inter-map overlaps highlighted by Table 4 reveal that the USGS NLCD class “*Pasture/Hay*” (PH, see Table 1) occurs together with high and medium canopy cover-related BC names in the SIAM-WELD map. The USGS NLCD class “*Cultivated crops*” (CC, see Table 1) matches with both SIAM’s spectral macro-categories MS green-as-“*Vegetation*” and MS brown-as-“*Bare soil or built-up*.” Finally, the USGS NLCD classes of wetland, specifically, “*Woody Wetlands*”, WW, and “*Emergent Herbaceous Wetland*,” EHW, see Table 1, overlap with the SIAM’s vegetated spectral macro-categories or with spectral macro-category MS blue-as-”*Water or Shadow*” (WA, refer to Table 2).

As reported in previous Chapter 4.2, in the OAMTRX = FrequencyCount(A × B) instance shown in Table 4, gray entry-pair cells were identified as “correct” by the independent human expert, based on the hybrid eight-step guideline proposed in the Part 1, Chapter 4. They identify the binary relationship, R: A ⇒ B ⊆ A × B ≠ OAMTRX = FrequencyCount(A × B), suitable for guiding the interpretation process in the OAMTRX at hand. In the OMATRX instance shown in Table 4, the mDMI set of O-Q^2^Is to be jointly maximized comprises summary statistics OA(OAMTRX = FrequencyCount(A × B)) = OA(Test SIAM-WELD 2006, 19 spectral macro-categories; Reference NLCD 2006, NLCD 16 classes) = 96.88% ± 0% with CVPAI2(R: A ⇒ B ⊆ A × C) = 0.6689. As a consequence, according to Equation (2),

OA(OAMTRX = FrequencyCount(A × B)) = OA(Test SIAM-WELD 2006, 19 spectral macro-categories; “Ultimate” GroundTruth 2006, NLCD 16 classes) ∈ [max{0%, Lower Bound}, min{100%, Upper Bound}] = [max{0%, OA(Test SIAM-WELD 2006, 19 spectral macro-categories; Reference NLCD 2006, NLCD 16 classes) ± 0% - 22%}, min{100%, 178% - OA(Test SIAM-WELD 2006, 19 spectral macro-categories; Reference NLCD 2006, NLCD 16 classes) ± 0%}] = [max{0%, 96.88% ± 0% - 22%}, min{100%, 178% - 96.88% ± 0%}] = [74.88%, 81.12%],

with

CVPAI2(R: A = SIAM vocabulary of 19 BC names ⇒ B = NLCD legend of 16 LC class names) =0.6689. (3)

Hence, the semantic information gap, to be minimized, from input sub-symbolic sensory data to output symbolic NLCD classes, left to be filled (disambiguated) by further stages in the hierarchical EO-IUS pipeline, where spatial information is masked by first-stage color names, see Figure 3, is equal to (1—CVPAI2) = 0.3311; refer to the Part 1, Chapter 5.

When disagreements between the two reference and test maps were back-projected onto the WELD 2006 image domain, these specific WELD sites were photointerpreted by the independent human expert to provide an additional independent source of thematic evidence for GEO-CEOS stage 4 *Val* of the annual SIAM-WELD 2006 test map in BC names. The large majority of the CONUS areas where the USGS NLCD vegetation classes overlap with the SIAM spectral macro-category MS blue-as-”*Water or Shadow*” (WA, refer to Table 2) or, vice versa, where the SIAM vegetation spectral macro-categories overlap with the USGS NLCD reference class “*Open Water*” (OW, refer to Table 1) were identified by the independent human photointerpreter as riparian zones. In practice, these riparian zones were labeled by the annual SIAM-WELD 2006 and NLCD 2006 maps in two different conditions of their annual surface status. Also in this case, the SIAM-WELD labeling appears consistent with the human photointerpretation of the annual WELD composite, irrespective of the semantic disagreement between this SIAM-WELD labeling and the reference USGS NLCD 2006 map.

Based on evidence collected by the independent photointerpreter with regard to systematic “conceptual mismatches” between the test SIAM-WELD 2006 map and the reference USGS NLCD 2006 map across nearly all desert areas and nearly all riparian zones of the CONUS, validated conclusions were twofold. First, according to Equation (1), where the reference USGS NLCD 2006 map is acknowledged to be no “ground truth” for the annual SIAM-WELD 2006 test map, but only a reference baseline for comparison purposes, “conceptual mismatches” between the test SIAM-WELD 2006 map and the reference USGS NLCD 2006 map should not be misconceived as mapping errors by the test SIAM-WELD 2006 map with respect to an “ultimate” ground truth. Second, according to Equation (3), summary statistic OA(OAMTRX = FrequencyCount(A × B) = OA(Test SIAM-WELD 2006, 19 spectral macro-categories; “Ultimate” GroundTruth 2006, NLCD 16 classes) was inferred to belong to range [74.88%, 81.12%], to be assessed in combination with a CVPAI2(R: A ⇒ B) value in range [0, 1], estimated equal to 0.6689.

It is important to recall here that for any given two-way frequency table OAMTRX = FrequencyCount(A × B) generated from two categorical variables A and B of the same population, where A ≠ B in general, the OAMTRX’s pair of O-Q^2^Is forming an mDMI set of quality indexes to be jointly maximized is OA(OAMTRX = FrequencyCount(A × B)) ∈ [0, 1] and CVPAI2(R: A ⇒ B ⊆ A × B ≠ OAMTRX = FrequencyCount(A × B)) ∈ [0, 1], where the latter, estimated from the binary relationship guiding the interpretation process of the OAMTRX at hand, is independent of the OA(OAMTRX) estimated value. Only if A = B then OAMTRX = (square and sorted) CMTRX whose main diagonal guides the interpretation process, CVPAI2(R: A ⇒ B) = 1 and OA(OAMTRX) ∈ [0, 1] becomes the sole O-Q^2^I informative per se about the degree of match between bivariate occurrences A and B.

#### 4.3.2. Test case B

To reveal the inherent ill-posedness of “conceptual matching” between two categorical variables A and B investigated by Ahlqvist (2005) (refer to the Part 1, Chapter 4), one co-author of this paper, different from the independent human expert (refer to Acknowledgments), conducted a second inherently equivocal selection of “correct” entry-pairs in a binary relationship, R: A ⇒ B ⊆ A × B, to guide the interpretation process of the OAMTRX = FrequencyCount(A × B) instance shown in Table 4. This second experiment provided an mDMI pair of O-Q^2^Is equal to OA(OAMTRX = FrequencyCount(A × B)) = 97.28% ± 0% and CVPAI2(R: A ⇒ B) = 0.6731. They are both superior to (better than) the pair of summary statistics OA(OAMTRX = FrequencyCount(A × B)) = 96.88% ± 0% and CVPAI2(R: A ⇒ B) = 0.6689 provided by the independent human expert in the test case A. This alternative binary relationship R: A ⇒ B looks sparser and therefore less intuitive to understand than that shown as gray entry-pair cells in Table 4. Hence, it is not shown in this paper, although it is made available via anonymous ftp (SIAM-WELD-NLCD FTP, 2016). When these summary statistics replace variables in Equation (2), we obtain

OA(OAMTRX = FrequencyCount(A × B)) = OA(Test SIAM-WELD 2006, 19 spectral macro-categories; “Ultimate” GroundTruth 2006, NLCD 16 classes) ∈ [max{0%, Lower Bound}, min{100%, Upper Bound}] = [max{0%, OA(Test SIAM-WELD 2006, 19 spectral macro-categories; Reference NLCD 2006, NLCD 16 classes) ± 0% - 22%}, min{100%, 178% - OA(Test SIAM-WELD 2006, 19 spectral macro-categories; Reference NLCD 2006, NLCD 16 classes) ± 0%}] =[max{0%, 97.28% ± 0% - 22%}, min{100%, 178% - 97.28% ± 0%}] =[75.28%, 80.72%],

with

CVPAI2(R: A = SIAM vocabulary of 19 BC names ⇒ B = NLCD legend of 16 LC class names) = 0.6731. (4)

Hence, the semantic information gap, to be minimized, from input sub-symbolic sensory data to output symbolic NLCD classes, left to be filled (disambiguated) by further stages in the hierarchical EO-IUS pipeline, where spatial information is masked by first-stage color names, see Figure 3, is equal to (1—CVPAI2) = 0. 3269; also refer to the Part 1, Chapter 5.

#### 4.3.3. Test case C

The wall-to-wall overlap between the test SIAM-WELD 2006 map, whose legend A = 96 BC names belonging to the SIAM fine color discretization level (refer to Table 1 in the Part 1 of this paper), and the reference USGS NLCD 2006 map, with legend B = 16 LC classes, generated another OAMTRX = FrequencyCount(A × B) instance, 96 × 16 cells in size, too large to be shown in a technical paper, but made available via anonymous ftp (SIAM-WELD-NLCD FTP, 2016). Once again, the hybrid inference procedure described in the Part 1, Chapter 4 was employed by the independent human expert to select “correct” entry-pairs in the binary relationship R: A ⇒ B ⊆ A × B eligible for guiding the interpretation process of this OAMTRX instance. Estimated mDMI set of O-Q^2^Is became OA(OAMTRX = FrequencyCount(A × B)) = 95.41% ± 0% and CVPAI2(R: A ⇒ B ⊆ A × B) = 0.5809. When these summary statistics replace variables in Equation (2), we obtained

OA(OAMTRX = FrequencyCount(A × B)) = OA(Test SIAM-WELD 2006, 96 BC names; “Ultimate” GroundTruth 2006, NLCD 16 classes) ∈ [max{0%, Lower Bound}, min{100%, Upper Bound}] = [max{0%, OA(Test SIAM-WELD 2006, 96 spectral categories; Reference NLCD 2006, NLCD 16 classes) ± 0% - 22%}, min{100%, 178% - OA(Test SIAM-WELD 2006, 96 spectral macro-categories; Reference NLCD 2006, NLCD 16 classes) ± 0%}] = [max{0%, 95.41% ± 0% - 22%}, min{100%, 178% - 95.41% ± 0%}] = [73.41%, 82.59%],

with

CVPAI2(R: A = SIAM vocabulary of 96 BC names ⇒ B = NLCD legend of 16 LC class names) = 0. 5809. (5)

Hence, the semantic information gap, to be minimized, from input sub-symbolic sensory data to output symbolic NLCD classes, left to be filled (disambiguated) by further stages in the hierarchical EO-IUS pipeline, where spatial information is masked by first-stage color names, see Figure 3, is equal to (1—CVPAI2 = 0. 4191); also refer to the Part 1, Chapter 5.

#### 4.3.4. Test case D

When the USGS NLCD 2006 classification taxonomy became less discriminative (coarser) because reassembled from its original 16 LC class names into either 9 LC class names (Stehman et al., 2008; Wickham et al., 2010, 2013), refer to previous Chapter 2, or 4 LC class names, see Table 1, constrained by inter-level parent-child relationships in agreement with the FAO LCCS-DP taxonomy, see Figure 1, then the following inequality holds true.

OA(Reference NLCD 2006, NLCD 16 classes; “Ultimate” GroundTruth 2006, NLCD 16 classes) = 78% (Wickham et al. 2010; Wickham et al. 2013; Stehman et al. 2008) ≤ OA(Reference NLCD 2006, “augmented” 3-level 9-class FAO LCCS-DP; “Ultimate” GroundTruth 2006, “augmented” 3-level 9-class FAO LCCS-DP) = 84% (Wickham et al. 2010; Wickham et al. 2013; Stehman et al. 2008) ≤OA(Reference NLCD 2006, 2-level 4-class FAO LCCS-DP taxonomy; “Ultimate” GroundTruth 2006, 2-level 4-class FAO LCCS-DP taxonomy) = XX%, hence, XX% ≥ 84%, (6)

where XX% is an unknown variable expected to remain unspecified because we have no chance to access the “Ultimate” GroundTruth 2006 dataset, adopted in past works to validate the USGS NLCD 2006 map (Stehman et al., 2008; Wickham et al., 2010, 2013), to reassemble its original “augmented” 3-level 9-class FAO LCCS-DP taxonomy into a 2-level 4-class FAO LCCS-DP taxonomy consisting of LC classes (see Figure 1):

- A1 = Primarily Vegetated Terrestrial Areas = Cultivated Areas (A11) or (Semi) Natural Vegetation (A12).
- A2 = Primarily Vegetated Aquatic or Regularly Flooded Areas = Cultivated Aquatic Areas (A23) or (Semi) Natural Aquatic Vegetation (A24).
- B3 = Primarily Non-vegetated Terrestrial Areas = Artificial Surfaces (B35) or Bare Areas (B36).
- B4 = Primarily Non-vegetated Aquatic or Regularly Flooded Areas = Artificial (B47) or Natural Waterbodies, Snow and Ice (B48).

In agreement with Equations (1), (2), and (6), we could write

OA(Test SIAM-WELD 2006, 19 spectral macro-categories; “Ultimate” GroundTruth 2006, 2-level 4-class FAO LCCS-DP taxonomy) ∈ [max{0%, Lower Bound}, min{100%, Upper Bound}] = [max{0%, OA(Test SIAM-WELD 2006, 19 spectral macro-categories; Reference NLCD 2006, 2-level 4-class FAO LCCS-DP taxonomy) ± 0% - (100% - XX%)}, min{100%, 100% + XX% - OA(Test SIAM-WELD 2006, 19 spectral macro-categories; Reference NLCD 2006, 2-level 4-class FAO LCCS-DP taxonomy) ± 0%}], where unknown variable XX% = Equation (6) = OA(Reference NLCD 2006, 2-level 4-class FAO LCCS-DP taxonomy; “Ultimate” GroundTruth 2006, 2-level 4-class FAO LCCS-DP taxonomy) ≥ 84%. (7)

As shown in the test case A, according to the USGS NLCD taxonomy definitions (refer to Table 1), LC classes “*Developed, Open Space*” (DOS), “*Developed, Low Intensity*” (DLI), “*Developed, Medium Intensity*” (DMI) and “*Developed, High intensity*” (DHI) describe a spatial mixture of vegetated surfaces, impervious surfaces and bare soil types, in agreement with the popular vegetation-impervious surface-soil model for urban ecosystem analysis (Ridd, 1995). It means that a logical OR combination of the USGS NLCD classes DOS or DLI or DMI or DHI mainly matches with 2-of-4 FAO LCCS-DP 2^nd^-level classes, either B3 or A1. Experts in the domain of world ontologies and in the harmonization of LC class taxonomies, the present authors concluded it is not possible to define without ambiguity each of the four LC classes in the 2-level 4-class FAO LCCS-DP taxonomy as a mutually exclusive and totally exhaustive parent-child relationship starting from the 16 LC classes in the USGS NLCD taxonomy. Nevertheless, to harmonize these two thematic map legends, we arbitrarily selected a sub-optimal binary relationship from set A = NLCD 16 LC class taxonomy to set B = 2-level 4-class FAO LCCS-DP taxonomy, constrained as a mutually exclusive and totally exhaustive parent-child relationship, reported in Table 5. Implemented grouping rules are summarized below.

- A1 = Primarily Vegetated Terrestrial Areas = Cultivated Areas or (Semi) Natural Vegetation ≈ NLCD 16-classes DF (41) or EF (42) or MF (43) or SS (52) or GH (71) or PH (81) or CC (82). Actually, this OR-combination of NLCD classes is an expected mixture of the FAO LCCS-DP 2^nd^-level classes A1 and B3 as first- and second-best match, respectively.
- A2 = Primarily Vegetated Aquatic or Regularly Flooded Areas = Cultivated Aquatic Areas or (Semi) Natural Aquatic Vegetation ≈ NLCD 16-classes WV (90) or EHW (95).
- B3 = Primarily Non-vegetated Terrestrial Areas = Artificial Surfaces or Bare Areas ≈ NLCD 16-classes DOS (21) or DLI (22) or DMI (23) or DHI (24) or BL (31). Actually, this OR-combination of NLCD classes is an expected mixture of the FAO LCCS-DP 2^nd^-level classes B3 and A1 as first- and second-best match, respectively.
- B4 = Primarily Non-vegetated Aquatic or Regularly Flooded Areas = Artificial or Natural Waterbodies, Snow and Ice ≈ NLCD 16-classes OW (11) or PIS (12).

Following the aforementioned “arbitrary” (subjective) aggregation of an NLCD 16-class legend into a 2-level 4-class FAO LCCS-DP legend, equivalent to an inherently equivocal (qualitative) *information-as-data-interpretation* task, we accomplished the following.

- A binary relationship R: A ⇒ C ⊆ A × C from set A = SIAM legend of 19 spectral macro-categories, identified by the independent human expert, to set C = 2-level 4-class FAO LCCS-DP legend, identified by the present authors, was (subjectively) identified by the present authors according to the hybrid eight-step strategy for categorical variable-pair relationship identification proposed in the Part 1, Chapter 4. Depicted as gray entry-pair cells in Table 5, this binary relationship is eligible for guiding the interpretation process of an OAMTRX = FrequencyCount(A × C).
- An OAMTRX = FrequencyCount(A × C) was generated by the wall-to-wall overlap between the test SIAM-WELD map with legend A = 19 spectral macro-categories as rows and the reference USGS NLCD map whose original 16-class legend was grouped into legend C = 2-level 4-class FAO LCCS-DP taxonomy as columns, see Table 5.

The mDMI set of O-Q^2^Is estimated from Table 5 were OA(OAMTRX = FrequencyCount(A × C)) = 93.09% ± 0% and CVPAI2(R: A ⇒ C ⊆ A × C) = 0.7486. When these summary statistics replace variables in Equation (7), we obtained

OA(Test SIAM-WELD 2006, 19 spectral macro-categories; “Ultimate” GroundTruth 2006, 2-level 4-class FAO LCCS-DP taxonomy) ∈ [max{0%, Lower Bound}, min{100%, Upper Bound}] = [max{0%, 93.09% ± 0% - (100% - XX%)}, min{100%, 100% + XX% - 93.09% ± 0%}] = [XX% - 6.91%, XX% + 6.91%], (8)

where

unknown variable XX% = Equation (6) = OA(Reference NLCD 2006, 2-level 4-class FAO LCCS-DP taxonomy; “Ultimate” GroundTruth 2006, 2-level 4-class FAO LCCS-DP taxonomy) ≥ 84%,

with

CVPAI2(R: A = SIAM vocabulary of 19 spectral macro-categories ⇒ C = 2-level 4-class FAO LCCS-DP taxonomy) = 0.7486.

Hence, the semantic information gap, to be minimized, from input sub-symbolic sensory data to output symbolic classes belonging to the 2-level 4-class FAO LCCS-DP legend, left to be filled (disambiguated) by further stages in the hierarchical EO-IUS pipeline, where spatial information is masked by first-stage color names, see Figure 3, is equal to (1—CVPAI2) = 0. 2514; refer to the Part 1, Chapter 5.

### 4.4. Probabilities of the SIAM-WELD test labels conditioned by the USGS NLCD reference labels and vice versa

Table 4 shows the OAMTRX instance generated from the wall-to-wall overlap of the annual SIAM-WELD 2006 test map featuring 19 spectral macro-categories, see Table 2 and Figure 8, with the reference USGS NLCD 2006 map featuring 16 LC classes, see Table 1 and Figure 4. The division of each probability cell of Table 4 by its column-sum generates the class-conditional probability *p*(SIAM-WELD*_t_ |* NLCD_r_) of the SIAM-WELD 2006 test spectral category *t*, with *t* = 1, …, *TC* = 19, given the USGS NLCD 2006 reference class *r*, with *r* = 1, …, *RC* = 16, refer to Figure 10 and Table 6, where Table 6 is a summarized text version of Figure 10. To prove their plausibility, conditional probabilities *p*(SIAM-WELD*_t_ |* NLCD_r_), *t* = 1, …, *TC, r* = 1, …, *RC*, should agree with theoretical expectations stemming from human experience. For instance, it was expected that the USGS NLCD 2006 reference classes “*Deciduous Forest*” (DF), “*Evergreen Forest*” (EF) and “*Mixed Forest*” (MF), refer to Table 1, overlap with vegetated spectral categories in the test SIAM-WELD 2006 map, while the USGS NLCD reference class “*Developed, High Intensity*” (DHI, see Table 1) was expected to be mostly matched by bare soil-related spectral macro-categories in the test SIAM-WELD 2006 map. Overall, these prior knowledge-based expectations about specific class-conditional probabilities appear satisfied by both Figure 10 and Table 6.

In the RS common practice, once a generic user has generated at no cost in manpower and computer power, that is, in near real time and without user-machine interaction, a SIAM color map from an unknown EO image, what this user wishes to do is to infer from the EO image a set of LC classes (say, “*Forest*”), conditioned by the detected SIAM’s BC names (say, MS green-as-“*Vegetation*”). To accomplish this spectral category-conditional inference, class-conditional probabilities *p*(SIAM-WELD*_t_ |* NLCD_r_), *t* = 1, …, *TC, r* = 1, …, *RC*, shown in Table 6, are not useful. Rather, this generic user can found helpful to know the conditional probabilities of an NLCD 2006 reference class *r*, with *r* = 1, …, *RC* = 16, given the SIAM-WELD 2006 spectral category *t*, with *t* = 1, …, *TC* = 19. These are the class-conditional probabilities *p*(NLCD_r_ | SIAM-WELD*_t_*), *t* = 1, …, *TC, r* = 1, …, *RC*, generated by dividing each probability cell of Table 4 by its row-sum. They are shown in Figure 11 and summarized in text form in Table 7. Very intuitive to understand, Table 7 clearly highlights the two main semantic inconsistencies found between the reference USGS NLCD 2006 map and the test SIAM-WELD 2006 map already reported in previous Chapter 4.3. First, the SIAM vegetation-related spectral macro-category wV_HC (“*Weak evidence vegetation with high canopy cover*”, refer to Table 2) is best matched by the reference NLDC class “*Open Water*” (OW, refer to Table 2). Because this semantic mismatch occurs almost exclusively in the CONUS areas recognized by the independent human expert as riparian zones typically depicted as mixed pixels at 30 m resolution, then the 30 m resolution SIAM labeling can be considered reasonable, if we consider that the crisp SIAM implementation is not expected to accomplish pixel unmixing. Second, the USGS NLCD 2006 reference class “*Shrub/Scrub*” (SS, refer to Table 2) appears to be the best match for several of the SIAM bare soil-related spectral macro-categories. Figure 9 shows examples of geographic locations where this semantic mismatch occurs. In these locations, 30 m resolution pixels are typically affected by mixed spectral contributions the crisp SIAM implementation is not expected to unmix.

### 4.5. Stratification by ecoregions

According to a simplistic interpretation of the central limit theorem, the sum of a large number of independent random variables tends to form a Gaussian distribution, where independent “local” data distributions (like basis functions) become indistinguishable from the whole. For example, in human vision, the neural computations are inherently spatially local in the (2D) image-domain; next, a global spatial average is superimposed on the local computational processes. In general, non-stationary local features do not survive the averaging process, that is, the precise position of each local contribution is no longer perceived after the averaging process (Victor, 1994). Because the WELD composite of the CONUS is about 10 billion pixels in size, summary statistics of the SIAM mapping quality at the CONUS spatial extent are inadequate to demonstrate the local-scale capability of the SIAM expert system to correctly map EO images, characterized by non-stationary local statistics. To investigate the SIAM mapping capability at local spatial extent, the test SIAM-WELD 2006 map with legend A of 19 BC names and the reference USGS NLCD 2006 map with legend B of 16 LC class names, where set A ≠ set B in cardinality and semantics, were stratified using the 86 EPA Level III ecoregions of the CONUS (see Figure 4) and an individual OAMTRX = FrequencyCount(A × B) was generated per ecoregion. All 86 ecoregion-specific OAMTRX instances are available as supplemental online material (SIAM-WELD-NLCD FTP, 2016). As one example of an inter-map comparison at the ecoregion spatial scale of analysis, let us consider the SIAM-WELD 2006 and NLCD 2006 maps of the Wyoming Basin ecoregion, which is predominantly desert, see Figure 12 where the ecoregion boundary is highlighted in red. Table 8 reports the corresponding OAMTRX instance. Table 8 shows that the predominantly desertic Wyoming Basin ecoregion is predominantly classified as the LC classes “*Scrub/Shrub*” (SS) and “*Grassland/Herbaceous*” (GH) in the reference USGS NLCD 2006 map (refer to Table 1) and as bare soil-related spectral categories (sbS_1, SmS_1, aS) in the test SIAM-WELD 2006 map (refer to Table 2). This semantic disagreement was already observed in previous Chapter 4.3; also refer to Figure 9.

**Table 3.** Spectral category-specific percentage of occurrences in the SIAM-WELD 2006/2007/2008/2009 test maps at the intermediate level of color quantization, where 48 basic color names were aggregated into 19 spectral macro-categories by an independent human expert. Adopted acronyms for the SIAM’s 19 spectral macro-categories: refer to Table 2

Spectral category	2006	2007	2008	2009	Mean	Std Dev
sV_HC	33.11%	32.56%	33.79%	34.06%	33.38%	0.68%
aV_HC	19.94%	23.31%	20.02%	20.86%	21.03%	1.57%
wV_HC	0.18%	0.17%	0.17%	0.19%	0.18%	0.01%
sV_MC	0.00%	0.00%	0.00%	0.00%	0.00%	0.00%
aV_MC	20.05%	18.79%	18.07%	17.93%	18.71%	0.97%
sV_LC	0.00%	0.00%	0.00%	0.00%	0.00%	0.00%
aV_LC	0.40%	0.18%	0.31%	0.22%	0.28%	0.10%
wbV_MLC	4.12%	3.60%	3.73%	3.30%	3.69%	0.34%
wdV_MLC	1.48%	1.37%	1.19%	1.53%	1.39%	0.15%
*Total vegetation*	*79.28%*	*79.98%*	*77.29%*	*78.10%*	*78.66%*	*1.20%*
sbS_1	5.00%	5.44%	6.28%	5.39%	5.53%	0.54%
sbS_2	0.09%	0.13%	0.08%	0.12%	0.11%	0.02%
smS_1	4.65%	3.51%	5.38%	4.78%	4.58%	0.78%
smS_2	0.19%	0.16%	0.24%	0.20%	0.20%	0.03%
sdS	0.25%	0.28%	0.25%	0.29%	0.27%	0.02%
aS	8.04%	8.18%	8.24%	8.70%	8.29%	0.29%
wS	0.02%	0.01%	0.01%	0.01%	0.01%	0.00%
*Total soils*	*18.24%*	*17.71%*	*20.48%*	*19.49%*	*18.98%*	*1.25%*
SN	0.01%	0.01%	0.02%	0.01%	0.01%	0.01%
WA	1.28%	1.28%	1.25%	1.27%	1.27%	0.02%
O	1.19%	1.02%	0.96%	1.13%	1.07%	0.10%

**Table 4.** Non-square OAMTRX = FrequencyCount(A × B) instance generated from a wall-to-wall overlap between the annual SIAM-WELD 2006 test map of the CONUS with legend A = 19 spectral macro-categories and the reference USGS NLCD 2006 map with legend B = 16 LC class names. Gray entry-pair cells identify the binary relationship R: A ⇒ B ⊆ A × B chosen by the independent human expert to guide the interpretation process of the OAMTRX = FrequencyCount(A × B). Statistically independent O-Q2Is, to be jointly maximized, are CVPAI2(R: A ⇒ B) ∈ [0, 1] (where value 1 means perfect harmonization between the two input sets A and B) = 0.6689 and OA(OAMTRX = FrequencyCount(A × B)) = 96.88%. Adopted acronyms for reference LC classes and test spectral macro-categories are described in Tables 1 and 2, respectively

2006 OAMTRX, probabilities (%). Rows: SIAM™-WELD 2006, 19 spectral categories; Columns: NLCD 2006, 16 land cover classes.																		
	NLCD code	11	12	21	22	23	24	31	41	42	43	52	71	81	82	90	95	
	NLCD class	OW	PIS	DOS	DLI	DMI	DHI	BL	DF	EF	MF	SS	GH	PH	CC	WW	EHW	
	FAO LCCD-DP1&2	B4	B4	B3 -> A1	B3 -> A1	B3 -> A1	B3 -> A1	B3	A1	A1	A1	A1 -> B3	A1 -> B3	A1	A1	A2	A2	
**SIAM™ Intermediate Granularity, 19 Spectral Categories.**	sV_HC	0.07%	0.00%	1.00%	0.13%	0.01%	0.00%	0.02%	9.51%	4.29%	1.73%	1.10%	0.77%	3.04%	8.35%	2.76%	0.31%	**33.11%**
	aV_HC	0.25%	0.00%	1.31%	0.82%	0.20%	0.02%	0.04%	1.38%	4.75%	0.33%	1.67%	1.66%	2.32%	3.71%	0.99%	0.50%	**19.94%**
	wV_HC	0.04%	0.00%	0.00%	0.01%	0.01%	0.00%	0.00%	0.00%	0.04%	0.00%	0.02%	0.01%	0.00%	0.01%	0.00%	0.02%	**0.18%**
	sV_MC	0.00%	0.00%	0.00%	0.00%	0.00%	0.00%	0.00%	0.00%	0.00%	0.00%	0.00%	0.00%	0.00%	0.00%	0.00%	0.00%	**0.00%**
	aV_MC	0.04%	0.00%	0.70%	0.28%	0.09%	0.01%	0.05%	0.48%	2.31%	0.06%	4.93%	6.66%	1.47%	2.55%	0.19%	0.23%	**20.05%**
	sV_LC	0.00%	0.00%	0.00%	0.00%	0.00%	0.00%	0.00%	0.00%	0.00%	0.00%	0.00%	0.00%	0.00%	0.00%	0.00%	0.00%	**0.00%**
	aV_LC	0.00%	0.00%	0.01%	0.00%	0.00%	0.00%	0.00%	0.00%	0.01%	0.00%	0.07%	0.27%	0.02%	0.02%	0.00%	0.00%	**0.40%**
	wbV_MLC	0.01%	0.00%	0.07%	0.07%	0.09%	0.03%	0.04%	0.00%	0.31%	0.00%	2.54%	0.79%	0.02%	0.13%	0.01%	0.01%	**4.12%**
	wdV_MLC	0.02%	0.00%	0.02%	0.03%	0.06%	0.02%	0.03%	0.01%	0.38%	0.00%	0.71%	0.12%	0.01%	0.05%	0.00%	0.03%	**1.48%**
	sbS_1	0.01%	0.00%	0.09%	0.04%	0.04%	0.02%	0.57%	0.00%	0.01%	0.00%	2.88%	0.90%	0.03%	0.38%	0.01%	0.01%	**5.00%**
	sbS_2	0.00%	0.00%	0.00%	0.00%	0.00%	0.01%	0.03%	0.00%	0.00%	0.00%	0.05%	0.00%	0.00%	0.00%	0.00%	0.00%	**0.09%**
	smS_1	0.01%	0.00%	0.05%	0.01%	0.00%	0.00%	0.04%	0.01%	0.04%	0.00%	2.09%	1.94%	0.03%	0.43%	0.00%	0.01%	**4.65%**
	smS_2	0.00%	0.00%	0.00%	0.00%	0.00%	0.00%	0.05%	0.00%	0.00%	0.00%	0.12%	0.00%	0.00%	0.00%	0.00%	0.00%	**0.19%**
	sdS	0.01%	0.00%	0.00%	0.00%	0.01%	0.01%	0.03%	0.00%	0.01%	0.00%	0.16%	0.01%	0.00%	0.00%	0.00%	0.00%	**0.25%**
	aS	0.01%	0.00%	0.08%	0.04%	0.04%	0.05%	0.17%	0.01%	0.12%	0.00%	5.47%	1.76%	0.02%	0.26%	0.01%	0.01%	**8.04%**
	wS	0.00%	0.00%	0.00%	0.00%	0.00%	0.00%	0.00%	0.00%	0.00%	0.00%	0.00%	0.01%	0.00%	0.00%	0.00%	0.00%	**0.02%**
	SN	0.00%	0.00%	0.00%	0.00%	0.00%	0.00%	0.00%	0.00%	0.00%	0.00%	0.00%	0.00%	0.00%	0.00%	0.00%	0.00%	**0.01%**
	WA	1.10%	0.00%	0.00%	0.00%	0.01%	0.01%	0.04%	0.00%	0.00%	0.00%	0.03%	0.01%	0.00%	0.01%	0.00%	0.05%	**1.28%**
	O	0.14%	0.00%	0.04%	0.04%	0.03%	0.01%	0.09%	0.01%	0.08%	0.00%	0.34%	0.11%	0.03%	0.23%	0.01%	0.02%	**1.19%**
		**1.71%**	**0.02%**	**3.36%**	**1.48%**	**0.59%**	**0.20%**	**1.21%**	**11.41%**	**12.35%**	**2.13%**	**22.19%**	**15.03%**	**6.99%**	**16.12%**	**3.99%**	**1.21%**	**100.00%**

**Table 5.** Non-square OAMTRX = FrequencyCount(A × C) instance generated from a wall-to-wall overlap between the annual SIAM-WELD 2006 test map of the CONUS with legend A = 19 spectral macro-categories and the reference USGS NLCD 2006 map with legend C = 16 LC class names grouped by the present authors into a 2-level 4-class FAO LCCS-DP taxonomy, see Figure 1, as proposed in Table 1. Gray entry-pair cells identify the binary relationship R: A ⇒ C ⊆ A × C chosen by the present authors to guide the interpretation process of the OAMTRX = FrequencyCount(A × C). Statistically independent O-Q^2^Is, to be jointly maximized, are OA(OAMTRX = FrequencyCount(A × C)) = 93.09% with CVPAI2(R: A ⇒ C ⊆ A × C) ∈ [0, 1] (where value 1 means perfect harmonization between the two input sets A and C) = 0.7486. Adopted acronyms for reference LC classes and test spectral macro-categories are described in Tables 1 and 2, respectively

	NLCD CODE (class acronym), 16 classes	41 (DF), 42 (EF), 43 (MF), 52 (SS) <-> B3, 71 (GH) <-> B3, 81 (PH), 82 (CC)	90 (WV), 95 (EHW)	21 (DOS) <-> A1, 22 (DLI) <-> A1, 23 (DMI) <-> A1, 24 (DHI) <-> A1, 31 (BL)	11 (OW), 12 (PIS)	
	FAO LCCS-DP1&2 Code, 4 classes	≈ A1	≈ A2	≈ B3	≈ B4	
	FAO LCCS-DP1&2 Class name, 4 classes	Veg terstrl	Veg aqutc	Non-veg terstrl	Non-veg aqutc	Sum per row
**SIAM™ Intermediate Granularity, 19 Spectral Categories.**	sV_HC	28.80%	3.07%	1.17%	0.07%	33.11%
	aV_HC	15.82%	1.49%	2.38%	0.25%	19.94%
	wV_HC	0.08%	0.03%	0.03%	0.04%	0.18%
	sV_MC	0.00%	0.00%	0.00%	0.00%	0.00%
	aV_MC	18.45%	0.42%	1.13%	0.05%	20.05%
	sV_LC	0.00%	0.00%	0.00%	0.00%	0.00%
	aV_LC	0.39%	0.00%	0.01%	0.00%	0.40%
	wbV_MLC	3.80%	0.02%	0.30%	0.01%	4.12%
	wdV_MLC	1.27%	0.04%	0.15%	0.02%	1.48%
	sbS_1	4.21%	0.01%	0.76%	0.01%	5.00%
	sbS_2	0.06%	0.00%	0.03%	0.00%	0.09%
	smS_1	4.53%	0.01%	0.10%	0.01%	4.65%
	smS_2	0.13%	0.00%	0.06%	0.00%	0.19%
	sdS	0.18%	0.00%	0.06%	0.01%	0.25%
	aS	7.63%	0.02%	0.38%	0.01%	8.04%
	wS	0.02%	0.00%	0.00%	0.00%	0.02%
	SN	0.00%	0.00%	0.00%	0.00%	0.01%
	WA	0.06%	0.05%	0.07%	1.11%	1.28%
	O	0.80%	0.03%	0.21%	0.14%	1.19%
	Sum per column	**86.23%**	**5.20%**	**6.84%**	**1.73%**

**Table 6.** Class-conditional probability p(SIAM-WELD*_t_* | NLCD *_r_*), *t* = 1, …, *TC *= |A| = 19 color names, *r* = 1, … *RC *= |B| = 16 LC class names. For each NLCD 2006 reference map’s LC class, the five best-matching SIAM-WELD 2006 test map’s spectral macro-categories, belonging to the finite set A of 19 spectral macro-categories, are shown as SIAM1 to SIAM5

NLCD class	SIAM1	p(SIAM1 | NLCD)	SIAM2	p(SIAM2 | NLCD)	SIAM3	p(SIAM3 | NLCD)	SIAM4	p(SIAM4 | NLCD)	SIAM5	p(SIAM5 | NLCD)
Open water	WA	0.64	aV_HC	0.15	O	0.08	sV_HC	0.04	wV_HC	0.03
Ice/Snow	SN	0.22	O	0.16	WA	0.10	aV_MC	0.10	aS	0.08
Developed, Open	aV_HC	0.39	sV_HC	0.30	aV_MC	0.21	sbS_1	0.03	aS	0.02
Developed, Low	aV_HC	0.55	aV_MC	0.19	sV_HC	0.09	wbV_MLC	0.05	sbS_1	0.03
Developed, Medium	aV_HC	0.33	wbV_MLC	0.15	aV_MC	0.15	wdV_MLC	0.10	aS	0.07
Developed, High	aS	0.24	wbV_MLC	0.16	wdV_MLC	0.11	sbS_1	0.11	aV_HC	0.08
Rock/Sand/Clay	sbS_1	0.48	aS	0.14	O	0.07	aV_MC	0.04	smS_2	0.04
Deciduous Forest	sV_HC	0.83	aV_HC	0.12	aV_MC	0.04	O	0.00	wdV_MLC	0.00
Evergreen Forest	aV_HC	0.38	sV_HC	0.35	aV_MC	0.19	wdV_MLC	0.03	wbV_MLC	0.03
Mixed Forest	sV_HC	0.81	aV_HC	0.16	aV_MC	0.03	O	0.00	wbV_MLC	0.00
Scrub/Shrub	aS	0.25	aV_MC	0.22	sbS_1	0.13	wbV_MLC	0.11	smS_1	0.09
Grassland/Herbaceous	aV_MC	0.44	smS_1	0.13	aS	0.12	aV_HC	0.11	sbS_1	0.06
Pasture/Hay	sV_HC	0.43	aV_HC	0.33	aV_MC	0.21	sbS_1	0.00	smS_1	0.00
Cultivated Crops	sV_HC	0.52	aV_HC	0.23	aV_MC	0.16	smS_1	0.03	sbS_1	0.02
Woody Wetlands	sV_HC	0.69	aV_HC	0.25	aV_MC	0.05	wbV_MLC	0.00	O	0.00
Herbaceous Wetland	aV_HC	0.41	sV_HC	0.26	aV_MC	0.19	WA	0.04	wdV_MLC	0.03

**Table 7.** Class-conditional probability p(NLCD*_r_* | SIAM-WELD*_t_*), *t* = 1, …, *TC *= |A| = 19 color names, *r* = 1, … *RC *= |B| = 16 LC class names. For each SIAM-WELD 2006 test map’s spectral macro-category, the five best-matching NLCD 2006 reference map’s LC classes, belonging to the finite set B of 16 LC classes, are shown as NLCD1 to NLCD5

SIAM	NLCD1	p(NLCD1 | SIAM)	NLCD2	p(NLCD2 | SIAM)	NLCD3	p(NLCD3 | SIAM)	NLCD4	p(NLCD4 | SIAM)	NLCD5	p(NLCD15| SIAM)
sV_HC	Deciduous Forest	0.29	Cultivated Crops	0.25	Evergreen Forest	0.13	Pasture/Hay	0.09	Woody Wetlands	0.08
aV_HC	Evergreen Forest	0.24	Cultivated Crops	0.19	Pasture/Hay	0.12	Shrub/Scrub	0.08	Grassland/Herbaceous	0.08
wV_HC	Open Water	0.25	Evergreen Forest	0.20	Herbaceous Wetlands	0.12	Shrub/Scrub	0.12	Developed, Medium	0.08
sV_MC	Deciduous Forest	0.33	Cultivated Crops	0.17	Pasture/Hay	0.15	Grassland/Herbaceous	0.08	Woody Wetlands	0.06
aV_MC	Grassland/Herbaceous	0.33	Shrub/Scrub	0.25	Cultivated Crops	0.13	Evergreen Forest	0.12	Pasture/Hay	0.07
sV_LC	Shrub/Scrub	0.04	Evergreen Forest	0.03	Grassland/Herbaceous	0.01	Woody Wetlands	0.01	Cultivated Crops	0.01
aV_LC	Grassland/Herbaceous	0.68	Shrub/Scrub	0.18	Cultivated Crops	0.05	Pasture/Hay	0.04	Evergreen Forest	0.02
wbV_MLC	Shrub/Scrub	0.62	Grassland/Herbaceous	0.19	Evergreen Forest	0.08	Cultivated Crops	0.03	Developed, Medium	0.02
wdV_MLC	Shrub/Scrub	0.48	Evergreen Forest	0.26	Grassland/Herbaceous	0.08	Developed, Medium	0.04	Cultivated Crops	0.03
sbS_1	Shrub/Scrub	0.58	Grassland/Herbaceous	0.18	Rock/Sandy/Clay	0.11	Cultivated Crops	0.08	Developed, Open	0.02
sbS_2	Shrub/Scrub	0.58	Rock/Sandy/Clay	0.27	Developed, High	0.06	Developed, Medium	0.03	Grassland/Herbaceous	0.02
smS_1	Shrub/Scrub	0.45	Grassland/Herbaceous	0.42	Cultivated Crops	0.09	Developed, Open	0.01	Rock/Sandy/Clay	0.01
smS_2	Shrub/Scrub	0.66	Rock/Sandy/Clay	0.25	Grassland/Herbaceous	0.03	Developed, Low	0.01	Developed, Medium	0.01
sdS	Shrub/Scrub	0.63	Rock/Sandy/Clay	0.13	Developed, High	0.05	Evergreen Forest	0.04	Grassland/Herbaceous	0.04
aS	Shrub/Scrub	0.68	Grassland/Herbaceous	0.22	Cultivated Crops	0.03	Rock/Sandy/Clay	0.02	Evergreen Forest	0.01
wS	Grassland/Herbaceous	0.71	Shrub/Scrub	0.14	Cultivated Crops	0.06	Pasture/Hay	0.03	Evergreen Forest	0.02
SN	Ice/Snow	0.47	Rock/Sandy/Clay	0.35	Grassland/Herbaceous	0.06	Shrub/Scrub	0.03	Cultivated Crops	0.03
WA	Open Water	0.86	Herbaceous Wetlands	0.04	Rock/Sandy/Clay	0.03	Shrub/Scrub	0.03	Grassland/Herbaceous	0.01
O	Shrub/Scrub	0.28	Cultivated Crops	0.20	Open Water	0.12	Grassland/Herbaceous	0.09	Rock/Sandy/Clay	0.07

**Table 8.** OAMTRX instance generated from a wall-to-wall overlap over the Wyoming Basin Ecoregion between the test SIAM-WELD 2006 map with legend A = 19 spectral macro-categories and the reference USGS NLCD 2006 map with legend B = 16 LC class names. Gray squares identify the binary relationship R: A ⇒ B ⊆ A × B chosen by the independent human expert to guide the interpretation process of the OAMTRX = FrequencyCount(A × B), same as in Table 4. The Wyoming Basin Ecoregion is predominantly desertic. It is classified as LC class “*Scrub/Shrub*” (SS) or LC class “*Grassland/Herbaceous*” (GH) in the USGS NLCD 2006 reference map (refer to Table 1), and predominantly as spectral macro-categories of bare soil (sbS_1, SmS_1, aS) in the SIAM-WELD 2006 test map (refer to Table 2). This phenomenon of large-scale “conceptual mismatch” between the USGS NLCD 2006 and SIAM-WELD 2006 thematic map pair is discussed thoroughly in Chapter 4.3

Wyoming Basin Ecoregion, 2006 OAMTRX. Probabilities (%). Rows: SIAM™-WELD 2006, 19 spectral categories; Columns: NLCD 2006, 16 land cover classes.																		
	NLCD code	11	12	21	22	23	24	31	41	42	43	52	71	81	82	90	95	
	NLCD class	OW	PIS	DOS	DLI	DMI	DHI	BL	DF	EF	MF	SS	GH	PH	CC	WW	EHW	
	FAO LCCD-DP1&2	B4	B4	B3 -> A1	B3 -> A1	B3 -> A1	B3 -> A1	B3	A1	A1	A1	A1 -> B3	A1 -> B3	A1	A1	A2	A2	
**SIAM™ Intermediate Granularity, 19 Spectral Categories.**	sV_HC	0.00%	0.00%	0.03%	0.01%	0.00%	0.00%	0.00%	0.03%	0.02%	0.01%	0.06%	0.02%	1.04%	0.23%	0.09%	0.11%	**1.65%**
	aV_HC	0.02%	0.00%	0.05%	0.03%	0.01%	0.00%	0.00%	0.07%	0.43%	0.02%	0.41%	0.13%	1.05%	0.16%	0.38%	0.35%	**3.11%**
	wV_HC	0.01%	0.00%	0.00%	0.00%	0.00%	0.00%	0.00%	0.00%	0.01%	0.00%	0.01%	0.00%	0.00%	0.00%	0.01%	0.00%	**0.05%**
	sV_MC	0.00%	0.00%	0.00%	0.00%	0.00%	0.00%	0.00%	0.00%	0.00%	0.00%	0.00%	0.00%	0.00%	0.00%	0.00%	0.00%	**0.00%**
	aV_MC	0.01%	0.00%	0.06%	0.02%	0.00%	0.00%	0.00%	0.08%	0.68%	0.01%	3.47%	1.21%	0.60%	0.06%	0.23%	0.42%	**6.85%**
	sV_LC	0.00%	0.00%	0.00%	0.00%	0.00%	0.00%	0.00%	0.00%	0.00%	0.00%	0.00%	0.00%	0.00%	0.00%	0.00%	0.00%	**0.00%**
	aV_LC	0.00%	0.00%	0.00%	0.00%	0.00%	0.00%	0.00%	0.00%	0.00%	0.00%	0.06%	0.01%	0.00%	0.00%	0.00%	0.00%	**0.07%**
	wbV_MLC	0.01%	0.00%	0.04%	0.03%	0.01%	0.00%	0.00%	0.00%	0.18%	0.00%	2.35%	0.55%	0.04%	0.02%	0.05%	0.07%	**3.35%**
	wdV_MLC	0.01%	0.00%	0.01%	0.00%	0.00%	0.00%	0.00%	0.00%	0.14%	0.00%	0.57%	0.05%	0.01%	0.00%	0.01%	0.01%	**0.81%**
	sbS_1	0.01%	0.00%	0.12%	0.04%	0.01%	0.00%	0.69%	0.00%	0.01%	0.00%	16.90%	5.66%	0.09%	0.04%	0.04%	0.11%	**23.72%**
	sbS_2	0.00%	0.00%	0.00%	0.00%	0.00%	0.00%	0.00%	0.00%	0.00%	0.00%	0.02%	0.01%	0.00%	0.00%	0.00%	0.00%	**0.03%**
	smS_1	0.00%	0.00%	0.12%	0.01%	0.00%	0.00%	0.03%	0.00%	0.06%	0.00%	32.71%	4.11%	0.04%	0.01%	0.02%	0.09%	**37.20%**
	smS_2	0.00%	0.00%	0.00%	0.00%	0.00%	0.00%	0.01%	0.00%	0.00%	0.00%	0.04%	0.02%	0.00%	0.00%	0.00%	0.00%	**0.07%**
	sdS	0.01%	0.00%	0.00%	0.00%	0.00%	0.00%	0.00%	0.00%	0.00%	0.00%	0.08%	0.01%	0.00%	0.00%	0.00%	0.00%	**0.10%**
	aS	0.02%	0.00%	0.17%	0.07%	0.01%	0.00%	0.08%	0.00%	0.13%	0.00%	17.86%	3.25%	0.05%	0.02%	0.03%	0.10%	**21.79%**
	wS	0.00%	0.00%	0.00%	0.00%	0.00%	0.00%	0.00%	0.00%	0.00%	0.00%	0.00%	0.00%	0.00%	0.00%	0.00%	0.00%	**0.00%**
	SN	0.00%	0.00%	0.00%	0.00%	0.00%	0.00%	0.00%	0.00%	0.00%	0.00%	0.00%	0.00%	0.00%	0.00%	0.00%	0.00%	**0.00%**
	WA	0.54%	0.00%	0.00%	0.00%	0.00%	0.00%	0.01%	0.00%	0.00%	0.00%	0.06%	0.01%	0.00%	0.00%	0.01%	0.01%	**0.64%**
	O	0.02%	0.00%	0.01%	0.01%	0.00%	0.00%	0.01%	0.00%	0.01%	0.00%	0.31%	0.06%	0.05%	0.03%	0.02%	0.03%	**0.56%**
		**0.65%**	**0.00%**	**0.61%**	**0.22%**	**0.04%**	**0.01%**	**0.83%**	**0.18%**	**1.67%**	**0.04%**	**74.91%**	**15.10%**	**2.97%**	**0.57%**	**0.89%**	**1.30%**	**100.00%**

Figure 13 provides a synthetic representation of the full dataset of 86 ecoregion-specific OAMTRX instances available as supplemental online material (SIAM-WELD-NLCD FTP, 2016). It shows for each of the 16 reference NLDC classes, with index *r* = 1, …, *RC* = 16, the box-and-whisker diagram of the USGS NLCD-class-conditional probabilities *p*(SIAM-WELD*_er, t_ |* NLCD*_er, r_*), with *t* = 1, …, *TC *= 19, collected across the 86 ecoregions, each ecoregion identified with an index *er* = 1, …, *ER* = 86. In each of the *TC* = 19 boxes of an NLCD class-specific boxplot, the median (shown as a horizontal line within the box) represents the general trend of the distribution and the dispersion around it describes the distribution variability across ecosystems. A small dispersion around the median value indicates a reference-to-test class mapping whose occurrence is nearly constant across ecosystems, while a large dispersion around the median indicates that occurrences of this inter-map relationship change significantly across ecosystems.

### 4.6. mDMI set of OP-Q^2^I values estimated by independent means in a GEO-CEOS stage 4 Val of the SIAM process and product

Described in the introduction to Chapter 4 (Duke, 2016), an mDMI set of OP-Q^2^Is was estimated by the independent human expert (refer to Acknowledgments) in compliance with a GEO-CEOS stage 4 *Val* of the SIAM product and process, input with a 30 m resolution annual WELD 2006 to 2009 image composite time-series of the CONUS. These *Val* results are summarized below.

- Process degree of automation. In line with theoretical expectations about expert systems (refer to previous Chapter 1), the SIAM computer program required neither user-defined parameters nor reference samples to run. Hence, its ease of use cannot be surpassed by any alternative inference approach.
- Outcome effectiveness. An mDMI set of O-Q^2^Is (Si Liu et al., 2011; Peng et al., 2005), comprising OA(OAMTRX = FrequencyCount(A × B)), CVPAI2(R: A ⇒ B ⊆ A × B), class-conditional probabilities *p*(*r* | *t*) of reference class *r* = *1*, …, *RC = *|B|, given test class *t* = 1, …, *TC *= |A|, and class-conditional probabilities *p*(*t* | *r*), with *r* = *1*, …, *RC = *|B|, *t* = 1, …, *TC *= |A|, was estimated in the four test cases described in previous Chapter 4.3 to Chapter 4.5.
- Process efficiency: run-time memory occupation and computation time. About run-time memory occupation, the SIAM computer program adopts a tile streaming implementation, where the dynamic memory (random access memory, RAM) maximum occupation is a known function of the tile size to be fixed in advance, irrespective of the image size. In these experiments the RAM maximum occupation was set equal to 800 MB, which can be considered a “small” RAM value. About computation time: when run on a Dell Power Edge 710 server with dual Intel Xeon @ 2.70 GHz processor with 64 GB of RAM and a 64-bit Linux operating system, the SIAM software application required less than 45 s to generate its complete set of per-image output products from a 7-band Landsat-7 ETM+ WELD tile of 5000 × 5000 pixels, which means about 8 h to map an annual WELD composite of the CONUS. In our data mapping workflow, such an output rate was not inferior to the input rate of an annual WELD composite being implemented or delivered to end-users. Hence, the SIAM computation time was considered equivalent to near real time, where the SIAM computational complexity increases linearly with image size.
- Process robustness to changes in the input dataset. The SIAM mapping consistency of the annual WELD composites from year 2006 to 2009 was estimated to be “high” at the CONUS spatial extent; refer to Chapter 4.2 to Chapter 4.5.
- Process robustness to changes in input parameters, if any. Because SIAM requires no user-defined parameter to run, its robustness to changes in input parameters cannot be surpassed by alternative approaches.
- Process maintainability/scalability/re-usability, to keep up with changes in users’ needs and sensor specifications. The multi-source SIAM physical model can be applied to any existing or future planned spaceborne/airborne MS imaging sensor provided with a radiometric calibration metadata file; refer to the existing literature (Baraldi et al., 2010a, 2010b, 2010c; Baraldi & Humber, 2015) and to previous Chapter 2.
- Outcome timeliness, defined as the time span between data acquisition and product generation. Because it is prior knowledge based and near real-time, the SIAM application reduces timeliness from image acquisition to color map generation to almost zero, equal to computation time, which increaseas linearly with image size.
- Outcome costs, monotonically increasing with manpower and computer power. Because it is prior knowledge based, therefore automated, and near real time in a standard laptop computer, the SIAM costs are almost negligible.

## 5. Discussion

Table 3 shows that the 30 m resolution annual SIAM-WELD map time-series for years 2006 to 2009 at the CONUS spatial extent featuring a SIAM intermediate color discretization legend of 48 BC names reassembled into 19 spectral macro-categories by the independent human expert (refer to previous Chapter 3) is characterized by a standard deviation of the annual frequency counts collected for each spectral macro-category lower than 1%, with the exception of two vegetation-related spectral macro-categories, specifically, aV_HC and aV_MC (see Table 2). These two larger variations in spectral category-specific annual frequency counts at the CONUS spatial extent can be attributed mostly to vegetation phenology. This was proved in previous Chapter 4.2: changes in phenology affect the monthly WELD and annual WELD image composites and, as a consequence, the data-derived SIAM-WELD maps. These numerical results agree with the a priori knowledge of RS experts about the CONUS surface dynamics, whose inter-annual LCC summary statistics are expected to score low. The conclusion is that observations stemming from the annual SIAM-WELD map time-series with a legend of 19 spectral macro-categories comply with the domain knowledge of RS experts about the LC and LCC dynamics in the geophysical domain of the CONUS.

The interpretation process of the OAMTRX = FrequencyCount(A × B) shown in Table 4, generated from the wall-to-wall overlap between the test SIAM-WELD 2006 map featuring a set A = VocabularyOfColorNames = 19 spectral macro-categories and the reference USGS NLCD 2006 map with a set B = LegendOfObjectClassNames = 16 LC classes, is guided by the inter-dictionary binary relationship R: A ⇒ B ⊆ A × B, whose entry-pair cells, shown in gray, were selected as “correct” by the independent human expert (refer to Acknowledgments) according to the hybrid eight-step guideline for identification of a categorical variable-pair relationship proposed in the Part 1, Chapter 4. Table 4 reveals one single systematic case of “conceptual mismatch” between the USGS NLCD 2006 reference vegetation classes “*Scrub/Shrub*” (SS) or “*Grassland/Herbaceous*” (GH, refer to Table 1) and the SIAM-WELD 2006 bare soil-related spectral macro-categories sbS_1, SmS_1, and aS (refer to Table 2). These inter-map semantic mismatches occur in geographical locations where the CONUS landscapes look like those shown in Figure 9. When these land surface types are observed from space with a Landsat-like spatial resolution of 30 m, a one-pixel surface area of 900 m^2^ becomes a spectral mixture of sparse vegetation, rangeland, cheatgrass, dry long grass and/or short grass as foreground, with a background of sand, clay and/or rocks, especially if the percentage of vegetation cover can be slightly above the 15% of total cover required by the USGS NLCD definitions of classes SS and GH (refer to Table 2). In these mixed pixels at 30 m resolution, the spectral detection of the vegetated component is impossible for a hard (crisp) classifier, while it would be more manageable by a fuzzy classifier (Baraldi, 2011). In these experiments, since the SIAM expert system is run in crisp mode (refer to Chapter 3), then no pixel unmixing strategy can be applied to diminish or avoid the observed case of “semantic mismatch.” The conclusion is that the “conceptual mismatch” between the USGS NLCD 2006 reference vegetation classes SS and GH and the SIAM-WELD 2006 bare soil-related spectral categories is a possible example of systematic disagreement between the test and reference thematic maps featuring the same spatial resolution whose occurrence should be carefully scrutinized by RS experts in comparison with an “ultimate” ground truth; see Figure 9.

A different strategy to aesthetically (rather than formally) remove the aforementioned inter-dictionary “conceptual mismatch” would be to change color names in the SIAM color map legend, without changing the SIAM decision tree for MS reflectance space hyperpolyhedralization. In other words, based on thematic evidence collected on an a posteriori basis from the USGS NLCD 2006 reference map, it would be possible to change color names attached to the SIAM-WELD 2006 map legend and consider that, at the Landsat spectral and spatial resolution of an annual WELD composite of the CONUS, the SIAM spectral categories sbS_1, SmS_1, and aS are more likely to map the USGS NLCD reference vegetation classes “*Shrub/Scrub*” (SS) or “*Grassland/herbaceous*” (GH) than bare soil surface types.

Starting from the same OAMTRX = FrequencyCount(A × B) shown in Table 4, two independent selections by two different RS experts of a binary relationship R: A ⇒ B ⊆ A × B suitable for guiding the interpretation process of the OAMTRX instance at hand provided two alternative mDMI pairs of O-Q^2^I values to be jointly maximized, namely, an OA_1(OAMTRX = FrequencyCount(A × B)) = 96.88% ± 0% with a CVPSI2_1(R: A ⇒ B ⊆ A × B) = 0.6689 in test case A and an OA_2(OAMTRX = FrequencyCount(A × B)) = 97.28% ± 0% with a CVPSI2_2(R: A ⇒ B ⊆ A × B) = 0.6731 in test case B. These alternative O-Q^2^I pairs highlight the inherent ill-posedness of any inter-dictionary conceptual harmonization, although a specific protocol to reduce heuristic decisions by human experts in the identification of a binary relationship R: A ⇒ B ⊆ A × B was proposed in the Part 1, Chapter 4. According to a Pareto multi-objective optimization principle, the latter O-Q^2^I value pair should be preferred to the former. This choice proves that the OA of the test SIAM-WELD 2006 map compared with the reference NLDC 2006 map scores “very high,” with a semantic information gap from sub-symbolic sensory data to symbolic NLCD classes left to be filled (disambiguated) by further stages in the hierarchical EO-IUS pipeline, where spatial information is masked by first-stage color names, see Figure 3, equal to (1—CVPSI2) = 0.3269.

At the fine discretization level of the SIAM-WELD 2006 test map, featuring a legend A = 96 BC names, another inter-map wall-to-wall overlap with the USGS NLCD 2006 reference map, whose legend B = 16 LC classes, provided an mDMI pair of O-Q^2^I values equal to OA(OAMTRX = FrequencyCount(A × B)) = 95.41% ± 0% and CVPAI2(R: A ⇒ B ⊆ A × B) = 0.5809 in test case C. When compared to the two pairs of O-Q^2^I values collected from the test case A and the test case B, this third O-Q^2^I value pair proves that a finer hyperpolyhedralization of the MS reflectance space for color naming is not necessarily more convenient to cope with by human experts in the stratification of an LC classification problem according to a spectral and spatial convergence-of-evidence approach, refer to Equation (3) in the Part 1 (Hunt & Tyrrell, 2012).

When an approximated binary relationship R: B ⇒ C ⊆ B × C was identified from set B = NLCD 16-class legend to set C = 2-level 4-class FAO LCCS-DP legend, see Figure 1, a binary relationship R: A ⇒ C ⊆ A × C was defined from set A = SIAM 19-class legend as rows to set C = 2-level 4-class FAO LCCS-DP legend as columns (reassembled from original columns of the 16-class legend B) and an OAMTRX = FrequencyCount(A × C) was generated by the wall-to-wall overlap between the test SIAM-WELD map with legend A and the reference USGS NLCD map with 16-class legend grouped into the 4-class legend C as reported in Table 5 (test case D), then O-Q^2^I values were OA(OAMTRX = FrequencyCount(A × C)) = 93.09% ± 0% with CVPAI2(R: A ⇒ C) = 0.7486. From these results, we could infer the following.

OA(OAMTRX = FrequencyCount(A × C)) = OA(Test SIAM-WELD 2006, 19 spectral macro-categories; “Ultimate” GroundTruth 2006, 2-level 4-class FAO LCCS-DP taxonomy) ∈ [XX% - 6.91%, XX% + 6.91%], where

unknown variable XX% = Equation (6) = OA(Reference NLCD 2006, 2-level 4-class FAO LCCS-DP taxonomy; “Ultimate” GroundTruth 2006, 2-level 4-class FAO LCCS-DP taxonomy) ≥ 84%,

with a semantic information gap, to be minimized, from input sub-symbolic sensory data to output symbolic classes belonging to the 2-level 4-class FAO LCCS-DP legend, left to be filled (disambiguated) by further stages in the hierarchical EO-IUS pipeline, where spatial information is masked by first-stage color names, see Figure 3, equal to (1 – CVPAI2) = 0. 2514, refer to the Part 1, Chapter 5.

This inference supports the thesis investigated by the present experimental work, where the off-the-shelf SIAM lightweight computer program for prior knowledge-based MS reflectance space hyperpolyhedralization into BC names was considered eligible for systematic ESA EO Level 2 SCM product generation, with an SCM legend consistent with the “augmented” 9-class 3-level FAO LCCS-DP taxonomy; more specifically, the SIAM color maps are consistent with the 2-level 4-class FAO LCCS-DP taxonomy; see Figure 1 (Baraldi, Tiede, Sudmanns, Belgiu, & Lang, 2016, 2017).

To complete the interpretation of the OAMTRX instance shown in Table 4, two histograms of class-conditional probabilities, shown in Figures 10 and 11 respectively, together with their summarized text versions, shown as Tables 6 and 7 respectively, were generated from the OAMTRX of interest. Figure 10 and Table 6 reveal that any test SIAM-WELD 2006 spectral category conditioned by one NLCD 2006 reference class appears consistent with the USGS NLCD class definition (refer to Table 1) and with an a priori domain knowledge of RS experts about the geophysical CONUS domain, spatially sampled at 30 m resolution. Analogously, Figure 11 and Table 7 show that any NLCD 2006 reference class conditioned by one SIAM-WELD 2006 spectral category appears consistent with the spectral properties of the SIAM color type and with an a priori domain knowledge of RS experts about the geophysical CONUS domain, depicted at 30 m resolution. To conclude, class-conditional probabilities generated from Table 4 appear reasonable and confirm the statistical plausibility of the OAMTRX instance shown in Table 4 as a whole.

Figure 13 shows that if, for example, the boxplot of the USGS NLCD 2006 reference class “*Developed, Open Space*” (DOS) is compared to that of reference class “*Developed, Low Intensity*” (DLI), “*Developed, Medium Intensity*” (DMI) and “*Developed, High Intensity*” (DHI, refer to Table 1), then a monotonic decrease of the class-conditional probability of the SIAM-WELD vegetation-related spectral categories conditioned by the USGS NLCD reference class and collected at a regional spatial extent corresponding to a population of 86 ecoregions is observed in parallel with a monotonic increase of the class-conditional probability of the SIAM-WELD bare soil-related spectral categories. This is perfectly consistent with the a priori domain knowledge of RS experts about the spatial and spectral properties of urban and industrial areas in the CONUS, in agreement with the popular vegetation-impervious surface-soil model for urban ecosystem analysis (Ridd, 1995). In addition, these boxplots confirm that, at the local spatial extent of individual ecoregions, the USGS NLCD 2006 reference classes “*Deciduous Forest*” (DF), “*Evergreen Forest*” (EF) and “*Mixed Forest*” (MF, refer to Table 1) are almost entirely (> 90%) covered by the SIAM-WELD vegetation-related spectral categories, in agreement with theoretical expectations about the SIAM-WELD test map. In line with preliminary outcomes discussed in previous Chapter 4.3 and in Figure 12, boxplots shown in Figure 13 confirm that the USGS NLCD 2006 reference classes “*Scrub/Shrub*” (SS) and “*Grassland/Herbaceous*” (GH) have a strong heterogeneity of matches with the SIAM-WELD 2006 spectral categories collected at the ecoregion spatial extent. This is tantamount to saying that spectral signatures of these NLCD classes feature a strong variability when collected at regional scale; also refer to Figure 9. More properties of the USGS NLCD 2006 class-specific box diagrams collected at the local spatial extent of ecoregions appear reasonable, based on a priori human knowledge of the geophysical CONUS domain at the ecoregion spatial extent. For example, first, the USGS NLCD 2006 reference classes “*Pasture/Hay*” (PH) and “*Cultivated Crops*” (CC, refer to Table 1) are largely matched across ecoregions by the SIAM-WELD vegetation-related spectral categories. Second, the USGS NLCD reference class “*Perennial Ice/Snow*” (PIS, refer to Table 1) is best matched across ecoregions by the SIAM-WELD spectral category MS white-as-“*Snow*” (SN, refer to Table 2). Third, across ecoregions, the USGS NLCD 2006 reference class “*Open water*” (OW, refer to Table 1) is best matched by the SIAM-WELD spectral category MS blue-as-“*Water or Shadow*” (WA, refer to Table 2). To summarize, collected at the local extent of ecoregions to account for non-stationary spatial properties, boxplots shown in Figure 13a are considered statistically and semantically consistent with the definitions of the two legends adopted by the test and reference maps, they agree with a priori domain knowledge of RS experts about the LC and LCC dynamics in the geophysical CONUS domain and appear consistent with global (non-stratified by ecoregions) statistics collected at the CONUS spatial extent, as reported in previous Chapter 4.2 to Chapter 4.4.

## 6. Conclusions

To pursue the GEO-CEOS visionary goal of a GEOSS implementation plan for years 2005–2015 not yet accomplished by the RS community, this interdisciplinary work aimed at filling an analytic and pragmatic information gap from EO big data to systematic ESA EO Level 2 product generation at the ground segment, never achieved to date by any EO data provider and postulated as necessary not sufficient precondition to GEOSS development. For the sake of readability, this paper was split into two, the preliminary Part 1 - Theory and the present Part 2 - Validation.

Provided with a relevant survey value, the Part 1 of this paper reviewed a long history of prior knowledge-based MS reflectance space partitioners for static color naming developed by the RS community for use in hybrid (combined deductive and inductive) EO-IUSs for EO image enhancement and classification tasks in operating mode, but never validated in compliance with the GEO-CEOS QA4EO *Cal/Val* requirements. Original contributions of the Part 1 include, first, an analytic expression of a “naïve” Bayes classifier proposed as a biologically plausible hybrid CV system suitable for convergence of color and spatial evidence. Second, a hybrid eight-step protocol was proposed to infer a binary relationship, R: A ⇒ B ⊆ A × B, from categorical variable A to categorical variable B estimated from the same population. This eight-step protocol is of practical use because identification of a binary relationship R: A ⇒ B is mandatory to guide the interpretation process of a bivariate frequency table, BIVRFTAB = FrequencyCount(A × B), where A ≠ B in general. Third, in compliance with the GEO-CEOS QA4EO *Val* guidelines, two original and alternative formulations, CVPAI2(R: A ⇒ B) ∈ [0, 1] and CVPAI3(R: A ⇒ B) ∈ [0, 1], were proposed as a categorical variable-pair normalized degree of association (harmonization, matching) in a binary relationship, R: A ⇒ B ⊆ A × B, from categorical variable A to categorical variable B estimated from the same population, where A ≠ B in general. When CVPAI2 or CVPAI3 is maximum, equal to 1, then the two categorical variables A and B are considered fully harmonized (reconciled). If A ≠ B, an mDMI set of O-Q^2^Is for a two-way frequency table BIVRFTAB = FrequencyCount(A × B) comprises OA(BIVRFTAB = FrequencyCount(A × B)) ∈ [0, 1] and CVPAI2(R: A ⇒ B) ∈ [0, 1] to be jointly maximized. Only if A = B then BIVRFTAB is equal to a well-known square and sorted CMTRX, intuitive to use because its main diagonal guides the interpretation process and where CVPAI2(R: A ⇒ B) = CVPAI3(R: A ⇒ B) = 1.

In the present Part 2, from an ensemble of expert systems for color naming found in the RS literature, an off-the-shelf SIAM lightweight computer program was selected as potential candidate for systematic ESA EO Level 2 SCM product generation in operating mode, to be submitted to a GEO-CEOS stage 4 *Val* by independent means, at large spatial extent and multiple time samples, in agreement with the GEO-CEOS QA4EO *Cal/Val* guidelines. The selected input data set was the 30 m resolution annual WELD image composite time-series in TOARF values from year 2006 to 2009 at the CONUS spatial extent. The selected reference map was the 30 m resolution USGS NLCD 2006 map of the U.S., whose legend consists of 16 LC classes. The original methodological contribution of the present Part 2 consists of a novel protocol for wall-to-wall inter-map comparison without sampling, where the test and reference thematic maps feature the same spatial resolution and spatial extent, but whose legends A and B are not the same and must be harmonized. These working hypotheses are fully complementary to those of traditional protocols for map accuracy assessment based on random sampling and a pair of coincident test and reference map legends.

Conclusions of the present Part 2 are twofold. First, in agreement with the definition of an information processing system in operating mode proposed in this work, the off-the-shelf SIAM software executable submitted to a GEO-CEOS stage 4 *Val* can be considered in operating mode because its whole set of OP-Q^2^I estimates scored “high.” Second, the off-the-shelf SIAM lightweight computer program in operating mode can be considered suitable for systematic generation of an ESA EO Level 2 SCM product instantiation whose legend agrees with the standard 2-level 4-class FAO LCCS-DP taxonomy (first DP level: vegetation vs non-vegetation, second DP level: aquatic vs terrestrial), preliminary to an “augmented” 3-level 9-class FAO LCCS-DP taxonomy, defined as a standard 3-level 8-class FAO LCCD-DP legend, see Figure 1, augmented with class “Others,” which includes quality layers cloud and cloud-shadow. At the CONUS spatial extent, the experimental portion of the present Part 2 inferred that OA(OAMTRX = FrequencyCount(A × C)) = OA(Test SIAM-WELD 2006, A = 19 spectral macro-categories; “Ultimate” GroundTruth 2006, C = 2-level 4-class FAO LCCS-DP taxonomy) ∈ [XX%—6.91%, XX% + 6.91%,], where unknown variable XX% = OA(Reference NLCD 2006, 2-level 4-class FAO LCCS-DP taxonomy; “Ultimate” GroundTruth 2006, 2-level 4-class FAO LCCS-DP taxonomy) ≥ 84%, with CVPAI2(R: A ⇒ C) = 0.7486. Hence, the semantic information gap, to be minimized, from input sensory data to the output 2-level 4-class FAO LCCS-DP legend left to be filled (disambiguated) by further stages in the EO-IUS pipeline following the SIAM first stage, see Figure 3, is equal to (1—CVPAI2) = 0.2514 ∈ [0, 1]. This is tantamount to saying that, although spatial information dominates color information in vision (Baraldi, 2017; Matsuyama & Hwang, 1990), the spatial context-insensitive SIAM expert system for color naming, whose legend A was a vocabulary of 19 color names equivalent to hyperpolyhedra in a MS reflectance space, when input with the annual WELD 2006 image composite of the CONUS and when compared with the reference USGS NLCD 2006 map, whose original legend B consisting of 16 LC class names was reassembled into a dictionary C of four LC classes belonging to a standard 2-level 4-class FAO LCCS-DP taxonomy, carried out a CVPAI2(R: A ⇒ C) estimate equal to 74.86% of the classification (discrimination) work required to disentangle the four LC classes of a standard 2-level 4-class FAO LCCS-DP taxonomy, with an OA(OAMTRX = FrequencyCount(A × C)) = [XX%—6.91%, XX% + 6.91%], where variable XX% ≥ 84%.

Ongoing developments of this work aim at systematic generation from multi-source MS imagery of an ESA EO Level 2 SCM product whose legend is the “augmented” 3-level 9-class FAO LCCS-DP taxonomy, which includes quality layers cloud and cloud-shadow, as a viable alternative to the non-standard ESA EO Level 2 SCM legend adopted by the SEN2COR software toolbox distributed by ESA to be run on user side. Preliminary OP-Q^2^Is collected from a prototypical implementation of the hybrid (combined physical model-based and statistical model-based) feedback EO-IUS architecture sketched in Figure 3, where convergence of color and spatial evidence is pursued, were considered encouraging (Baraldi, 2015, 2017; Baraldi et al., 2016, 2017; Tiede, Baraldi, Sudmanns, Belgiu, & Lang, 2016). Degrees of novelty of the hybrid EO-IUS under development for systematic ESA EO Level 2 product generation include, first, a novel 2D wavelet-based spatial filter bank for automated image-contour detection and image segmentation (raw primal sketch, in the Marr terminology), consistent with human visual perception, such as the Mach bands illusion (Baraldi, 2017). Second, a “universal” hybrid spatial context-sensitive cloud/cloud-shadow detector in single-date MS imagery, eligible for use with past, present, and future optical imaging sensors provided with metadata *Cal* parameters (Baraldi, 2015, 2017 2015), alternative to existing cloud/cloud-shadow detectors, including that implemented in the SEN2COR software toolbox. Third, an original mDMI set of scale-invariant planar shape indexes (Baraldi, 2017; Baraldi & Soares, 2017), which includes a novel implementation of a straightness-of-boundaries indicator (Nagao & Matsuyama, 1980), particularly useful to discriminate managed (man-made, anthropic) LC classes from natural or semi-natural surface types, such as in the 3^rd^-level FAO LCCS-DP taxonomy, where LC class A11 (Cultivated and Managed Terrestrial Vegetated Areas) must be discriminated from LC class A12 (Natural and Semi-Natural Terrestrial Vegetation) and where LC class B35 (Artificial Surfaces and Associated Areas) must be separated from LC class B36 (Bare Areas); see Figure 1.

## Abbreviation

AI: Artificial Intelligence
ATCOR: Atmospheric/Topographic Correction commercial softare product
AVHRR: Advanced Very High Resolution Radiometer
BC: Basic Color
BIVRFTAB: Bivariate Frequency Table
Cal: Calibration
Cal/Val: Calibration and Validation
CCRS: Canada Centre for Remote Sensing
CEOS: Committee on Earth Observation Satellites
CLC: CORINE Land Cover (taxonomy)
CMTRX: Confusion Matrix
CNES: Centre national d’études spatiales
CONUS: Conterminous U.S.
CORINE: Coordination of Information on the Environment
CV: Computer Vision
CVPAI: Categorical Variable Pair Association (matching) Index (in range [0, 1])
DCNN: Deep Convolutional Neural Network
DLR: Deutsches Zentrum für Luft- und Raumfahrt (German Aerospace Center)
DN: Digital Number
DP: Dichotomous Phase (in the FAO LCCS taxonomy)
DRIP: Data-Rich, Information-Poor (scenario)
EDC: EROS Data Center
EO-IU: EO Image Understanding
EO-IUS: EO-IU System
EPA: Environmental Protection Agency
EROS: Earth Resources Observation Systems
ESA: European Space Agency
FAO: Food and Agriculture Organization
GEO: Intergovernmental Group on Earth Observations
GEOSS: Global EO System of Systems
GIGO: Garbage In, Garbage Out principle of error propagation
GIS: Geographic Information System
GIScience: Geographic Information Science
GUI: Graphic User Interface
HS: Hyper-Spectral
IGBP: International Global Biosphere Programme
IUS: Image Understanding System
LC: Land Cover
LCC: Land Cover Change
LCCS: Land Cover Classification System (taxonomy)
LCLU: Land Cover Land Use
LEDAPS: Landsat Ecosystem Disturbance Adaptive Processing System
mDMI: Minimally Dependent and Maximally Informative (set of quality indicators)
MHP: Modular Hierarchical Phase (in the FAO LCCS taxonomy)
MIR: Medium InfraRed
MODIS: Moderate Resolution Imaging Spectroradiometer
MS: Multi-Spectral
NASA: National Aeronautics and Space Administration
NIR: Near InfraRed
NLCD: National Land Cover Data
NOAA: National Oceanic and Atmospheric Administration
OA: Overall Accuracy
OAMTRX: Overlapping Area Matrix
OBIA: Object-Based Image Analysis
ODGIS: Ontology-Driven GIS
OGC: Open Geospatial Consortium
OP: Outcome (product) and Process
O-Q2Is: Outcome Quantitative Quality Index
OP-Q^2^I: Outcome and Process Quantitative Quality Index
P-Q2Is: Product Quantitative Quality Index
QA: Quality Assurance
QA4EO: Quality Accuracy Framework for Earth Observation
Q^2^I: Quantitative Quality Indicator
R&D: Research and Development
RGB: monitor-typical Red-Green-Blue data cube
RMSE: Root Mean Square Error
RS: Remote Sensing
SCM: Scene Classification Map
SDC: Single-Date Classification
SEN2COR: Sentinel 2 (atmospheric) Correction Prototype Processor
SQ^2^I: Spatial Quantitative Quality Index
SIAM™: Satellite Image Automatic Mapper™
STRATCOR: Stratified Topographic Correction
SS: Super-Spectral
SURF: Surface Reflectance
TIR: Thermal InfraRed
TM (superscript): (non-registered) Trademark
TOA: Top-Of-Atmosphere
TOARF: TOA Reflectance
TQ^2^I: Thematic Quantitative Quality Index
UML: Unified Modeling Language
USGS: US Geological Survey
Val: Validation
VQ: Vector Quantization
WELD: Web-Enabled Landsat Data set
WGCV: Working Group on Calibration and Validation

## Figures and Tables

**Figure:**
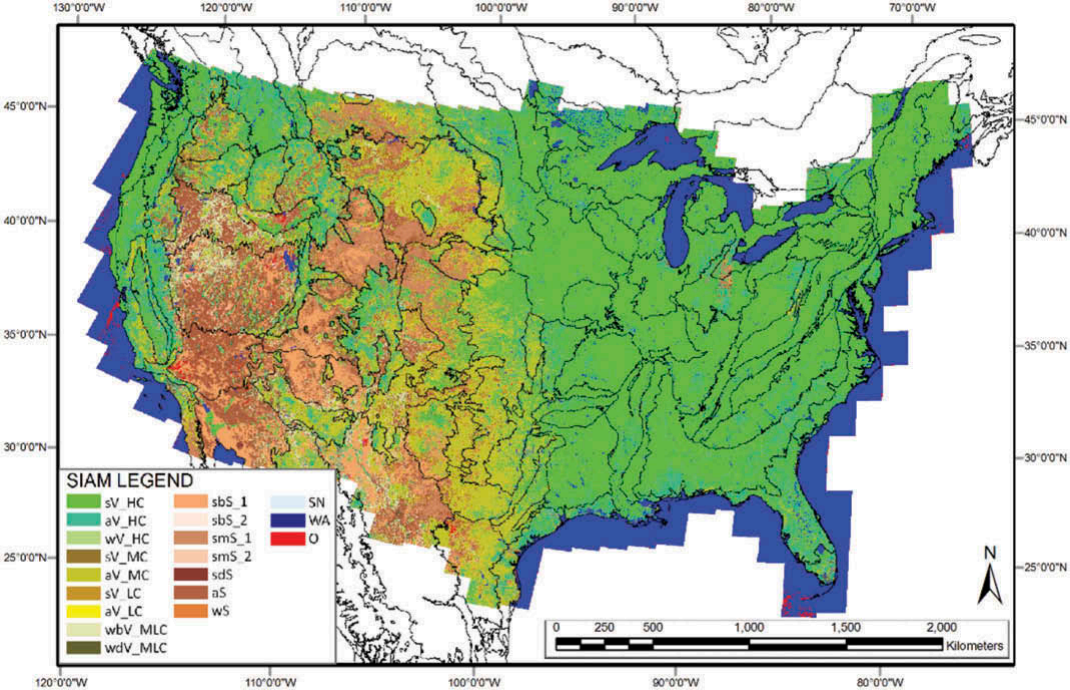


## Cover Image

Source: Satellite Image Automatic Mapper (SIAM) multi-level map generated automatically, without human-machine interaction, and in near real-time, specifically, in linear time complexity with image size, from the 30 m resolution annual Web-Enabled Landsat Data (WELD) image composite for the year 2006 of the conterminous U.S. (CONUS), radiometrically calibrated into top-of-atmosphere reflectance (TOARF) values. The multi-level map’s legend, shown at bottom left, is the SIAM intermediate discretization level, consisting of 48 basic color (BC) names reassembled into 19 spectral macro-categories by an independent human expert, according to a proposed hybrid (combined deductive and inductive) eight-step protocol for identification of a categorical variable-pair binary relationship, from a vocabulary of BC names to a dictionary of land cover (LC) class names. No discrete and finite vocabulary of color names, such as SIAM’s, equivalent to a set of mutually exclusive and totally exhaustive (hyper)polyhedra, neither necessarily convex nor connected, in a MS reflectance (hyper)space, should ever be confused with a symbolic (semantic) taxonomy of LC class names in the 4D geospace-time scene-domain. Black lines across the SIAM-WELD 2006 color map represent the boundaries of the 86 Environmental Protection Agency (EPA) Level III ecoregions of the CONUS, suitable for regional-scale statistical stratification required to intercept geospatial non-stationary statistics, typically lost when a global spatial average, e.g., at continental spatial extent, is superimposed on the local computational processes.

## References

[CIT0001] Ackerman S. A., Strabala K. I., Menzel W. P., Frey R. A., Moeller C. C., & Gumley L. E. (1998). Discriminating clear sky from clouds with MODIS. *Journal of Geophysical Research*, 103(32), 141–52. doi:10.1029/1998JD200032

[CIT0002] Ahlqvist O. (2005). Using uncertain conceptual spaces to translate between land cover categories. *International Journal of Geographical Information Science*, 19, 831–857.

[CIT0003] Arvor D., Madiela B. D., & Corpetti T. (2016). Semantic pre-classification of vegetation gradient based on linearly unmixed Landsat time series. Geoscience and Remote Sensing Symposium (IGARSS), IEEE International (pp. 4422–4425).

[CIT0004] Baraldi A. (2011). Fuzzification of a crisp near-real-time operational automatic spectral-rule-based decision-tree preliminary classifier of multisource multispectral remotely sensed images. *IEEE Transactions on Geoscience and Remote Sensing*, 49, 2113–2134. doi:10.1109/TGRS.2010.2091137

[CIT0005] Baraldi A. (2015). “Automatic Spatial Context-Sensitive Cloud/Cloud-Shadow Detection in Multi-Source Multi-Spectral Earth Observation Images – AutoCloud+,” Invitation to tender ESA/AO/1-8373/15/I-NB – “VAE: Next Generation EO-based Information Services”, DOI: 10.13140/RG.2.2.34162.71363 arXiv: 1701.04256. Retrieved Jan., 8, 2017 from https://arxiv.org/ftp/arxiv/papers/1701/1701.04256.pdf

[CIT0006] Baraldi A. (2017). Pre-processing, classification and semantic querying of large-scale earth observation spaceborne/airborne/terrestrial image databases: Process and product innovations”. Ph.D. dissertation in Agricultural and Food Sciences, University of Naples “Federico II”. *Department of Agricultural Sciences, Italy. Ph.D. defense: 16 May 2017*. doi:10.13140/RG.2.2.25510.52808 Retrieved 1, 30 2018, from https://www.researchgate.net/publication/317333100_Pre-processing_classification_and_semantic_querying_of_large-scale_Earth_observation_spaceborneairborneterrestrial_image_databases_Process_and_product_innovations

[CIT0007] Baraldi A., & Boschetti L. (2012a). Operational automatic remote sensing image understanding systems: Beyond Geographic Object-Based and Object-Oriented Image Analysis (GEOBIA/GEOOIA) – part 1: Introduction. *Remote Sensing*, 4, 2694–2735. doi:10.3390/rs4092694

[CIT0008] Baraldi A., & Boschetti L. (2012b). Operational automatic remote sensing image understanding systems: Beyond Geographic Object-Based and Object-Oriented Image Analysis (GEOBIA/GEOOIA) – part 2: Novel system architecture, information/knowledge representation, algorithm design and implementation. *Remote Sensing*, 4, 2768–2817.

[CIT0009] Baraldi A., Boschetti L., & Humber M. (2014). Probability sampling protocol for thematic and spatial quality assessments of classification maps generated from spaceborne/airborne very high resolution images. *IEEE Transactions on Geoscience and Remote Sensing*, 52(1), 701–760. doi:10.1109/TGRS.2013.2243739

[CIT0010] Baraldi A., Durieux L., Simonetti D., Conchedda G., Holecz F., & Blonda P. (2010a). Automatic spectral rule-based preliminary classification of radiometrically calibrated SPOT-4/-5/IRS, AVHRR/ MSG,AATSR, IKONOS/QuickBird/OrbView/GeoEye and DMC/SPOT-1/-2 imagery – part I: System design and implementation. *IEEE Transactions on Geoscience and Remote Sensing*, 48, 1299–1325. doi:10.1109/TGRS.2009.2032457

[CIT0011] Baraldi A., Durieux L., Simonetti D., Conchedda G., Holecz F., & Blonda P. (2010b). Automatic spectral rule-based preliminary classification of radiometrically calibrated SPOT-4/-5/IRS, AVHRR/ MSG, AATSR, IKONOS/QuickBird/OrbView/GeoEye and DMC/SPOT-1/-2 imagery – part II: Classification accuracy assessment. *IEEE Transactions on Geoscience and Remote Sensing*, 48, 1326–1354. doi:10.1109/TGRS.2009.2032064

[CIT0012] Baraldi A., Gironda M., & Simonetti D. (2010c). Operational three-stage stratified topographic correction of spaceborne multi-spectral imagery employing an automatic spectral rule-based decision-tree preliminary classifier. *IEEE Transactions Geosci Remote Sensing*, 48(1), 112–146. doi:10.1109/TGRS.2009.2028017

[CIT0013] Baraldi A., & Humber M. (2015). Quality assessment of pre-classification maps generated from spaceborne/airborne multi-spectral images by the Satellite Image Automatic Mapper™ and Atmospheric/Topographic Correction™-Spectral Classification software products: Part 1 – theory. *IEEE Journal of Selected Topics in Applied Earth Observations and Remote Sensing*, 8(3), 1307–1329.

[CIT0014] Baraldi A., Humber M., & Boschetti L. (2013). Quality assessment of pre-classification maps generated from spaceborne/airborne multi-spectral images by the Satellite Image Automatic Mapper™ and Atmospheric/Topographic Correction™-Spectral Classification software products: Part 2 – experimental results. *Remote Sensing*, 5, 5209–5264. doi:10.3390/rs5105209

[CIT0015] Baraldi A., Puzzolo V., Blonda P., Bruzzone L., & Tarantino C. (2006). Automatic spectral rule-based preliminary mapping of calibrated Landsat TM and ETM+ images. *IEEE Transactions on Geoscience and Remote Sensing*, 44, 2563–2586. doi:10.1109/TGRS.2006.874140

[CIT0016] Baraldi A., & Soares J. V. B. (2017). Multi-objective software suite of two-dimensional shape descriptors for object-based image analysis. *Subjects: Computer Vision and Pattern Recognition (cs.CV), arXiv:1701.01941*. Retrieved 18, 2017, from [Online] https://arxiv.org/ftp/arxiv/papers/1701/1701.01941.pdf

[CIT0017] Baraldi A., Tiede D., Sudmanns M., Belgiu M., & Lang S. (2016). *Automated near real-time earth observation level 2 product generation for semantic querying*. Enschede, The Netherlands: GEOBIA 2016, 14-16 Sept., University of Twente Faculty of Geo-Information and Earth Observation (ITC).

[CIT0018] Baraldi A., Tiede D., Sudmanns M., Belgiu M., & Lang S. (2017, 3 28–30). *Systematic ESA EO level 2 product generation as pre-condition to semantic content-based image retrieval and information/knowledge discovery in EO image databases* . 2017 Conf. on Big Data From Space, BiDS’17, Toulouse, France.

[CIT0019] Baraldi A., Wassenaar T., & Kay S. (2010d). Operational performance of an automatic preliminary spectral rule-based decision-tree classifier of spaceborne very high resolution optical images. *IEEE Transactions on Geoscience and Remote Sensing*, 48, 3482–3502. doi:10.1109/TGRS.2010.2046741

[CIT0020] Beauchemin M., & Thomson K. (1997). The evaluation of segmentation results and the overlapping area matrix. *International Journal of Remote Sens*, 18, 3895–3899. doi:10.1080/014311697216720

[CIT0021] Benavente R., Vanrell M., & Baldrich R. (2008). Parametric fuzzy sets for automatic color naming. *Journal of the Optical Society of America*, 25, 2582–2593. doi:10.1364/JOSAA.25.002582 18830336

[CIT0022] Berlin B., & Kay P. (1969). *Basic color terms: Their universality and evolution*. Berkeley, CA: University of California.

[CIT0023] Bernus P., & Noran O. (2017). Data rich – but information poor In Camarinha-Matos L., Afsarmanesh H., & Fornasiero R. (Eds.), *Collaboration in a data-rich world: PRO-VE 2017. IFIP advances in information and communication technology* (Vol. 506, pp. 206–214).

[CIT0024] Bishop C. M. (1995). *Neural networks for pattern recognition*. Oxford, UK: Clarendon.

[CIT0025] Bishop M. P., & Colby J. D. (2002). Anisotropic reflectance correction of SPOT-3 HRV imagery. *International Journal of Remote Sens*, 23(10), 2125–2131. doi:10.1080/01431160110097231

[CIT0026] Bishop M. P., Shroder J. F., & Colby J. D. (2003). Remote sensing and geomorphometry for studying relief production in high mountains. *Geomorphology*, 55(1–4), 345–361. doi:10.1016/S0169-555X(03)00149-1

[CIT0027] Boschetti L., Flasse S. P., & Brivio P. A. (2004). Analysis of the conflict between omission and commission in low spatial resolution dichotomic thematic products: The Pareto boundary. *Remote Sensing of Environment*, 91, 280–292. doi:10.1016/j.rse.2004.02.015

[CIT0028] Boschetti L., Roy D. P., Justice C. O., & Humber M. L. (2015). MODIS–Landsat fusion for large area 30 m burned area mapping. *Remote Sensing of Environment*, 161, 27–42. doi:10.1016/j.rse.2015.01.022

[CIT0029] Capurro R., & Hjørland B. (2003). The concept of information. *Annual Review of Information Science and Technology*, 37, 343–411. doi:10.1002/aris.1440370109

[CIT0030] Cherkassky V., & Mulier F. (1998). *Learning from data: Concepts, theory, and methods*. New York, NY: Wiley.

[CIT0031] CNES (2015). *Venμs satellite sensor level 2 product* . Retrieved 15, 2016, from https://venus.cnes.fr/en/VENUS/prod_l2.htm

[CIT0032] Congalton R. G, & Green K. (1999). Assessing the accuracy of remotely sensed data In *Boca raton, fl*. USA: Lewis Publishers.

[CIT0033] Dai X., & Khorram S. (1998). The effects of image misregistration on the accuracy of remotely sensed change detection. *IEEE Transactions on Geoscience and Remote Sensing*, 36, 1566–1577. doi:10.1109/36.718860

[CIT0034] Despini F., Teggi S., & Baraldi A. (2014, 9 22). Methods and metrics for the assessment of pan-sharpening algorithms In Bruzzone L., Benediktsson J. A., & Bovolo F. (Eds.), *SPIE proceedings, Vol. 9244: Image and signal processing for remote sensing XX*. Amsterdam, Netherlands.

[CIT0035] Deutsches Zentrum für Luft- und Raumfahrt e.V. (DLR) and VEGA Technologies (2011). *Sentinel-2 MSI – Level 2A products algorithm theoretical basis document. Document S2PAD-ATBD-0001*. European Space Agency.

[CIT0036] Di Gregorio A., & Jansen L. (2000). Land cover classification system (LCCS): Classification concepts and user manual.*FAO: Rome, Italy, FAO Corporate Document Repository*. Retrieved 210, 2015, from http://www.fao.org/DOCREP/003/X0596E/X0596e00.htm

[CIT0037] Dillencourt M. B., Samet H., & Tamminen M. (1992). A general approach to connected component labeling for arbitrary image representations. *Journal of Association for Computing Machinery*, 39, 253–280. doi:10.1145/128749.128750

[CIT0038] Dorigo W., Richter R., Baret F., Bamler R., & Wagner W. (2009). Enhanced automated canopy characterization from hyperspectral data by a novel two step radiative transfer model inversion approach. *Remote Sensing*, 1, 1139–1170. doi:10.3390/rs1041139

[CIT0039] Duke Center for Instructional Technology (2016). *Measurement: Process and outcome indicators* . Retrieved 620, 2016, from http://patientsafetyed.duhs.duke.edu/module_a/measurement/measurement.html

[CIT0040] Environmental Protection Agency (EPA) (2007). *“Definitions” in multi-resolution land characteristics consortium (MRLC)* . Retrieved 1113, 2013, from http://www.epa.gov/mrlc/definitions.html#2001

[CIT0041] Environmental Protection Agency (EPA) (2013). *Western ecology division* . Retrieved 1113, 2013, from http://www.epa.gov/wed/pages/ecoregions.htm

[CIT0042] European Space Agency (ESA) (2015). *Sentinel-2 User Handbook, Standard Document, Issue 1 Rev 2*.

[CIT0043] Foody G (2016). *“Observations on accuracy assessments of object-based image classifications,” GEOBIA 2016, 14-16 sept*. University of Twente, Faculty of Geo-Information and Earth Observation (ITC), Enschede, The Netherlands.

[CIT0044] Foody G. (2006). The evaluation and comparison of thematic maps derived from remote sensing.In Proc. of the 7th International Symposium on spatial accuracy in natural resources and environmental sciences Caetano M. (& Painho M., eds), 7-9 7, 2006, Lisbon. Instituto Geográfico Português, Lisbon, 18-31.

[CIT0045] GeoTerraImage (2015). *Provincial and national land cover 30 m* . Retrieved 922, 2015, from http://www.geoterraimage.com/productslandcover.php

[CIT0046] Gevers T., Gijsenij A., Van De Weijer J., & Geusebroek J. M. (2012). *Color in computer vision*. Hoboken, NJ: Wiley.

[CIT0047] Griffin L. D. (2006). Optimality of the basic color categories for classification. *Journal of the Royal Society, Interface*, 3, 71–85. doi:10.1098/rsif.2005.0076 PMC1618485 16849219

[CIT0048] Griffith G. E., & Omernik J. M. (2009). Ecoregions of the United States-Level III (EPA) In Cleveland C. J. (Ed.), *Encyclopedia of earth*. Washington, D.C.: Environmental Information Coalition, National Council for Science and the Environment.

[CIT0049] Group on Earth Observation (GEO) (2005). The global earth observation system of systems (GEOSS) 10-year implementation plan, adopted 16 February 2005. Retrieved 110, 2012, from http://www.earthobservations.org/docs/10-Year%20Implementation%20Plan.pdf

[CIT0050] Group on Earth Observation/Committee on Earth Observation Satellites (GEO/CEOS) (2010). A quality assurance framework for earth observation, version 4.0. Retrieved 1115, 2012, from http://qa4eo.org/docs/QA4EO_Principles_v4.0.pdf

[CIT0051] Group on Earth Observation/Committee on Earth Observation Satellites (GEO-CEOS) - Working Group on Calibration and Validation (WGCV) (2015). *Land product validation* (LPV). Retrieved 320, 2015, from http://lpvs.gsfc.nasa.gov/

[CIT0052] Hadamard J (1902). Sur les problemes aux deriveespartielles et leur signification physique. *Princet University Bulletin*, 13, 49–52.

[CIT0053] Homer C., Huang C. Q., Yang L. M., Wylie B., & Coan M. (2004). Development of a 2001 National Land-Cover Database for the United States. *Photogrammetric Engineering & Remote Sensing*, 70, 829–840. doi:10.14358/PERS.70.7.829

[CIT0054] Hunt N., & Tyrrell S. (2012). *Stratified sampling* . *Coventry University*. Retrieved 27, 2012from http://www.coventry.ac.uk/ec/~nhunt/meths/strati.html

[CIT0055] IBM (2016). *The four V’s of big data, IBM big data & analytics hub* .Retrieved 130, 2018, from http://www.ibmbigdatahub.com/infographic/four-vs-big-data

[CIT0056] Kuzera K., & Pontius R. G. (2008). Importance of matrix construction for multiple-resolution categorical map comparison. *GIScience & Remote Sensing*, 45, 249–274. doi:10.2747/1548-1603.45.3.249

[CIT0057] Lee D. S., Storey J. C., Choate M. J., & Hayes R. (2004). Four years of Landsat-7 on-orbit geometric calibration and performance. *IEEE Transactions on Geoscience and Remote Sensing*, 42, 2786–2795. doi:10.1109/TGRS.2004.836769

[CIT0058] Liang S. (2004). *Quantitative remote sensing of land surfaces*. Hoboken, NJ: Wiley.

[CIT0059] Lillesand T., & Kiefer R. (1979). *Remote sensing and image interpretation*. New York, NY: Wiley.

[CIT0060] Lipson H. (2007). Principles of modularity, regularity, and hierarchy for scalable systems. *Journal of Biological Physics and Chemistry*, 7, 125–128. doi:10.4024/40701.jbpc.07.04

[CIT0061] Liu S., Hairong Liu L. J., Latecki S. Y., Xu C., & Lu H. (2011). Size adaptive selection of most informative features. *Association for the Advancement of Artificial Intelligence*.

[CIT0062] Lück W., & Van Niekerk A. (2016). Evaluation of a rule-based compositing technique for Landsat-5 TM and Landsat-7 ETM+ images. *International Journal of Applied Earth Observation and Geoinformation*, 47, 1–14. doi:10.1016/j.jag.2015.11.019

[CIT0063] Lunetta R., & Elvidge D. (1999). *Remote sensing change detection: Environmental monitoring methods and applications*. London, UK: Taylor & Francis.

[CIT0064] Luo Y., Trishchenko A. P., & Khlopenkov K. V. (2008). Developing clear-sky, cloud and cloud shadow mask for producing clear-sky composites at 250-meter spatial resolution for the seven MODIS land bands over Canada and North America. *Remote Sensing of Environment*, 112, 4167–4185. doi:10.1016/j.rse.2008.06.010

[CIT0065] Markham B., & Helder D. (2012). *Forty-year calibrated record of earth-reflected radiance from Landsat: A review*. Paper 70 NASA Publications.

[CIT0066] Marr D. (1982). *Vision*. New York, NY: Freeman and C.

[CIT0067] Matsuyama T., & Hwang V. S. (1990). *SIGMA – a knowledge-based aerial image understanding system*. New York, NY: Plenum Press.

[CIT0068] Muirhead, K., & Malkawi, O. (1989, September 5–8). Proceedings Fourth AVHRR Data Users Meeting (pp. 31–34). Rottenburg, Germany.

[CIT0069] Nagao M., & Matsuyama T. (1980). *A structural analysis of complex aerial photographs*. New York, NY: Plenum.

[CIT0070] National Aeronautics and Space Administration (NASA) (2016). *Getting petabytes to people: How the EOSDIS facilitates earth observing data discovery and use. [Online]* . Retrieved 1229, 2016, from https://earthdata.nasa.gov/getting-petabytes-to-people-how-the-eosdis-facilitates-earth-observing-data-discovery-and-use

[CIT0071] Open Geospatial Consortium (OGC) Inc (2015). *OpenGIS® implementation standard for geographic information – simple feature access – part 1: Common architecture* . Retrieved 38, 2015, from http://www.opengeospatial.org/standards/is

[CIT0072] Ortiz A., & Oliver G. (2006). On the use of the overlapping area matrix for image segmentation evaluation: A survey and new performance measures. *Pattern Recognition Letters*, 27, 1916–1926. doi:10.1016/j.patrec.2006.05.002

[CIT0073] Peng H., Long F., & Ding C. (2005). Feature selection based on mutual information: Criteria of max-dependency, max-relevance, and min-redundancy. *IEEE Transactions Pattern Analysis Machine Intelligent*, 27, 1226–1238. doi:10.1109/TPAMI.2005.159 16119262

[CIT0074] Pontius R. G. Jr., & Connors J. (2006, 7 5–7). Expanding the conceptual, mathematical and practical methods for map comparison In Caetano M. & Painho M. (Eds). *Proceedings of the 7th international symposium on spatial accuracy assessment in natural resources and environmental sciences* (pp. 64–79). Lisbon: Instituto Geográfico Português.

[CIT0075] Pontius R. G. Jr., & Millones M. (2011). Death to Kappa: Birth of quantity disagreement and allocation disagreement for accuracy assessment. *International Journal of Remote Sensing*, 32(15), 4407–4429. doi:10.1080/01431161.2011.552923

[CIT0076] Riano D., Chuvieco E., Salas J., & Aguado I. (2003). Assessment of different topographic corrections in landsat-TM data for mapping vegetation types. *IEEE Transactions on Geoscience and Remote Sensing*, 41(5), 1056–1061. doi:10.1109/TGRS.2003.811693

[CIT0077] Richter R., & Schläpfer D. (2012a). *Atmospheric/topographic correction for satellite imagery – ATCOR-2/3 User Guide, Version 8.2 BETA* . Retrieved 412, 2013, from http://www.dlr.de/eoc/Portaldata/60/Resources/dokumente/5_tech_mod/atcor3_manual_2012.pdf

[CIT0078] Richter R., & Schläpfer D. (2012b). *Atmospheric/Topographic correction for airborne imagery – ATCOR-4 User Guide, Version 6.2 BETA, 2012* . Retrieved 412, 2013, from http://www.dlr.de/eoc/Portaldata/60/Resources/dokumente/5_tech_mod/atcor4_manual_2012.pdf

[CIT0079] Ridd M. K. (1995). Exploring a V-I-S (vegetation-impervious surface-soil) model for urban ecosystem analysis through remote sensing: Comparative anatomy for cities. *International Journal of Remote Sens*, 16(12), 2165–2185. doi:10.1080/01431169508954549

[CIT0080] Roy D., Ju J., Kline K., Scaramuzza P. L., Kovalskyy V., Hansen M., … Zhang C. S. (2010). Web-enabled Landsat data (WELD): Landsat ETM plus composited mosaics of the conterminous United States. *Remote Sensing of Environment*, 114, 35–49. doi:10.1016/j.rse.2009.08.011

[CIT0081] SIAM-WELD-NLCD FTP (2016). Retrieved 1213, 2016, from http://tinyurl.com/j4nzwzl

[CIT0082] Simonetti D., Simonetti E., Szantoi Z., Lupi A., & Eva H. D. (2015). First results from the phenology based synthesis classifier using Landsat-8 imagery. *IEEE Geoscience and Remote Sensing Letters*, 12(7), 1496–1500. doi:10.1109/LGRS.2015.2409982

[CIT0083] Sonka M., Hlavac V., & Boyle R. (1994). *Image processing, analysis and machine vision*. London, UK: Chapman & Hall.

[CIT0084] Stehman S. V., & Czaplewski R. L. (1998). Design and analysis for thematic map accuracy assessment: Fundamental principles. *Remote Sensing of Environment*, 64, 331–344. doi:10.1016/S0034-4257(98)00010-8

[CIT0085] Stehman S. V., Wickham J. D., Wade T. G., & Smith J. H. (2008). Designing a multi-objective, multi-support accuracy assessment of the 2001 National Land Cover Data (NLCD 2001) of the conterminous United States. *Photogrammetric Engineering & Remote Sensing*, 74, 1561–1571. doi:10.14358/PERS.74.12.1561

[CIT0086] Tiede D., Baraldi A., Sudmanns M., Belgiu M., & Lang S. (2016, 3 15–17). *ImageQuerying (IQ) – earth observation image content extraction & querying across time and space, submitted (oral presentation and poster session)* . ESA 2016 Conf. on Big Data From Space, BIDS ’16, Santa Cruz de Tenerife, Spain.

[CIT0087] Vermote E., & Saleous N. (2007). *LEDAPS surface reflectance product description – Version 2.0*. University of Maryland at College Park/Dept Geography and NASA/GSFC Code 614.5

[CIT0088] Victor J. (1994). Images, statistics, and textures: Implications of triple correlation uniqueness for texture statistics and the Julesz conjecture: Comment. *Journal of Optical Social American A*, 11(5), 1680–1684. doi:10.1364/JOSAA.11.001680

[CIT0089] Vogelmann J. E., Howard S. M., Yang L., Larson C. R., Wylie B. K., & Van Driel N. (2001). Completion of the 1990s National Land Cover Data set for the conterminous United States from Landsat Thematic Mapper data and ancillary data sources. *Photogrammetric Engineering & Remote Sensing*, 67, 650–662.

[CIT0090] Vogelmann J. E., Sohl T. L., Campbell P. V., & Shaw D. M. (1998). Regional land cover characterization using Landsat Thematic Mapper data and ancillary data sources. *Environmental Monitoring and Assessment*, 51, 415–428. doi:10.1023/A:1005996900217

[CIT0091] Web-Enabled Landsat Data (WELD) Tile FTP Retrieved 1212, 2016, from https://weld.cr.usgs.gov/

[CIT0092] Wessels K., Van Den Bergh F., Roy D., Salmon B., Steenkamp K., MacAlister B., … Jewitt D. (2016). Rapid land cover map updates using change detection and robust random forest classifiers. *Remote Sensing*, 8(888), 1–24. doi:10.3390/rs8110888

[CIT0093] Wickham J. D., Stehman S. V., Fry J. A., Smith J. H., & Homer C. G. (2010). Thematic accuracy of the USGS NLCD 2001 land cover for the conterminous United States. *Remote Sensing of Environment*, 114, 1286–1296. doi:10.1016/j.rse.2010.01.018

[CIT0094] Wickham J. D., Stehman S. V., Gass L., Dewitz J., Fry J. A., & Wade T. G. (2013). Accuracy assessment of NLCD 2006 land cover and impervious surface. *Remote Sensing of Environment*, 130, 294–304. doi:10.1016/j.rse.2012.12.001

[CIT0095] Xian G., & Homer C. (2010). Updating the 2001 National Land Cover Database impervious surface products to 2006 using Landsat imagery change detection methods. *Remote Sensing of Environment*, 114, 1676–1686. doi:10.1016/j.rse.2010.02.018

[CIT0096] Yang C., Huang Q., Li Z., Liu K., & Hu F. (2017). Big data and cloud computing: Innovation opportunities and challenges. *International Journal of Digital Earth*, 10(1), 13–53. doi:10.1080/17538947.2016.1239771

